# Molecular determinants of acrylamide neurotoxicity through covalent docking

**DOI:** 10.3389/fphar.2023.1125871

**Published:** 2023-03-02

**Authors:** Nicolas Pierre Friedrich Mueller, Paolo Carloni, Mercedes Alfonso-Prieto

**Affiliations:** ^1^ Institute for Advanced Simulations IAS-5, Institute of Neuroscience and Medicine INM-9, Computational Biomedicine, Forschungszentrum Jülich, Jülich, Germany; ^2^ Faculty of Mathematics and Natural Sciences, Heinrich Heine University Düsseldorf, Düsseldorf, Germany; ^3^ Department of Physics, RWTH Aachen University, Aachen, Germany; ^4^ Cécile and Oskar Vogt Institute for Brain Research, Medical Faculty, University Hospital Düsseldorf, Heinrich Heine University Düsseldorf, Düsseldorf, Germany

**Keywords:** acrylamide, covalent adduct, Michael addition reaction, neurotoxicicity, covalent docking

## Abstract

Acrylamide (ACR) is formed during food processing by Maillard reaction between sugars and proteins at high temperatures. It is also used in many industries, from water waste treatment to manufacture of paper, fabrics, dyes and cosmetics. Unfortunately, cumulative exposure to acrylamide, either from diet or at the workplace, may result in neurotoxicity. Such adverse effects arise from covalent adducts formed between acrylamide and cysteine residues of several neuronal proteins *via* a Michael addition reaction. The molecular determinants of acrylamide reactivity and its impact on protein function are not completely understood. Here we have compiled a list of acrylamide protein targets reported so far in the literature in connection with neurotoxicity and performed a systematic covalent docking study. Our results indicate that acrylamide binding to cysteine is favored in the presence of nearby positively charged amino acids, such as lysines and arginines. For proteins with more than one reactive Cys, docking scores were able to discriminate between the primary ACR modification site and secondary sites modified only at high ACR concentrations. Therefore, docking scores emerge as a potential filter to predict Cys reactivity against acrylamide. Inspection of the ACR-protein complex structures provides insights into the putative functional consequences of ACR modification, especially for non-enzyme proteins. Based on our study, covalent docking is a promising computational tool to predict other potential protein targets mediating acrylamide neurotoxicity.

## 1 Introduction

Acrylamide (CH_2_ = CH-C(O)NH_2_, PubChem CID 6579) is used in variety of industrial processes, including water waste treatment, manufacture of paper, fabrics, dyes or cosmetics (Swaen et al., 2007; Pennisi et al., 2013; Bušová et al., 2020). In addition, it is a by-product of the food industry, formed by Maillard reaction of reduced sugars and amino acids (Mottram et al., 2002) and present in food items processed at high temperatures (e.g. coffee, french fries and baked and roasted potatoes) (Reynolds, 2002; Guenther et al., 2007; Schouten et al., 2020). Due to the potential toxic effects of acrylamide in the human body (Semla et al., 2017; Kumar et al., 2018), in 2018 new European Union wide regulations entered into force (EU Commission, 2017) to prevent and/or reduce acrylamide formation in foodstuffs, e.g. during frying, baking or roasting.

Cumulative exposure to acrylamide, either from diet or at the workplace, may result in toxicity, especially at the level of the central nervous system. Animal and clinical studies suggest that acrylamide neurotoxicity could mimic the symptoms or even contribute to the etiology of neurodegenerative disorders like Parkinson’s disease (LoPachin and Gavin, 2012; Erkekoglu and Baydar, 2014; Li et al., 2015; Murray et al., 2020), as well as result in depression and anxiety-like behavioral effects (Faria et al., 2018, 2019; Raldúa et al., 2020). Three possible mechanisms have been proposed for acrylamide neurotoxicity: (i) inhibition of fast axonal transport, (ii) alteration of neurotransmitter levels, and (iii) direct inhibition of neurotransmission (LoPachin et al., 2007, 2008; Pruser and Flynn, 2011; LoPachin and Gavin, 2012; Erkekoglu and Baydar, 2014; Semla et al., 2017). In addition, acrylamide has been shown to indirectly increase oxidative stress by depleting the levels of the antioxidant glutathione (Catalgol et al., 2009; Kopanska et al., 2015; Raldúa et al., 2020).

**FIGURE 1:**
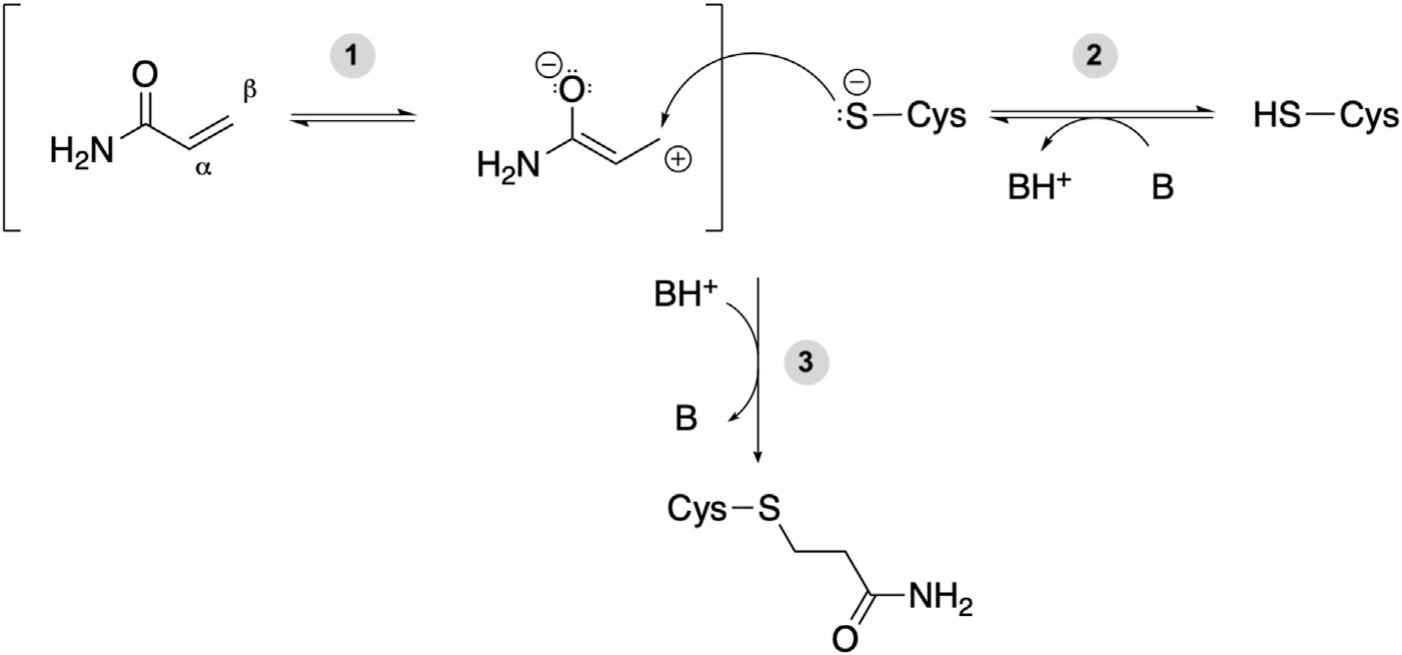
Michael addition reaction between acrylamide and a Cys residue of a target protein. B and BH^+^ represent a Brønsted-Lowry acid-base pair, either a protein residue or a water molecule. (1) The electron-withdrawing effect of the carbonyl group makes the *β*-carbon of acrylamide an electrophilic site. (2) The deprotonated cysteine side chain can act as nucleophile and react with a soft electrophile such as acrylamide. (3) Michael addition reaction between a protein thiolate and acrylamide yields a covalent adduct potentially affecting protein function.

Acrylamide (ACR) contains an *α*, *β*-unsaturated carbonyl that acts as an electrophile and thus is able to react with nucleophilic amino acids (Figure 1). The electron-withdrawing effect of the carbonyl group on the alkene makes the *β*-carbon the most electrophilic site (Figure 1, step 1). Based on the hard and soft acids and bases (HSAB) theory, a soft electrophile as ACR is expected to preferentially react with soft nucleophiles, such as the thiolate group of deprotonated cysteine residues (Koutsidis et al., 2009) (Figure 1, step 2). Although the intrinsic pK_a_ of the Cys side chain is 8.6 (and thus it is expected to be present as a thiol at physiological pH), several factors, such as hydrogen bonding or the presence of positively charged amino acids in the vicinity, can decrease its pK_a_ value. Therefore, certain protein microenviroments can favor the formation of a negatively charged thiolate (Roos et al., 2013). Michael addition reaction of such deprotonated Cys with acrylamide results in the formation of a covalent adduct (Figure 1, step 3). The amide group of the covalent adduct is able to act both as hydrogen bond acceptor (*via* the carbonyl group) and donor (through the amino group). Such H-bonds with nearby protein residues will help stabilize the covalent adduct and may result in alterations in protein function.

The molecular determinants of acrylamide reactivity with cysteines in proteins are not completely understood. Mass spectrometry-based proteomics analysis has identified proteins that can be modified upon acrylamide incubation (Barber et al., 2007; Feng and Lu, 2011; Nagashima et al., 2019; Zhao et al., 2019). However, such studies are often performed with high ACR concentrations, resulting in e.g. ACR reaction with N-terminal residues, whose modification is unlikely to alter significantly protein function and thus have toxic effects. *In vitro* and biochemical studies focusing on individual protein targets have pinpointed Cys residues whose modification by ACR may have an impact on protein function. However, such assays are not always readily available, especially for non-enzyme proteins. Screening of large protein databases for ACR targets and prediction of the most likely reactive sites in a given protein could be sped up using computational approaches. In this regard, the webservers Cy-preds (Soylu and Marino, 2015), Cpipe (Soylu and Marino, 2017) and pCysMod (Li et al., 2021) have been successfully used to predict Cys reactivity for disulfide bridge formation, metal binding, enzymatic catalysis and/or post-translational modifications. However, the underlying algorithms are not tailored to predict cysteines modified by ACR and do not provide information on the impact of the ACR covalent adduct on protein function. Hence, here we have assessed whether covalent docking could be used as a computational tool to characterize ACR reactivity and its (neuro)toxic effects at the molecular level. In particular, as a follow-up of previous computational works by some of us (Ferreira de Lima and Carloni, 2011; Papamokos et al., 2014), we compiled a list of acrylamide protein targets and performed a systematic molecular study using covalent docking. We focused on neuronal protein targets associated with neurotoxic symptoms of acrylamide, as well as acrylamide-modified proteins detectable in plasma and liver that can be used as biomarkers to monitor ACR exposure. Based on the analysis of the modeled acrylamide-protein complexes, we conclude that acrylamide modification is favored in the presence of nearby positively charged amino acids, such as lysine and arginine. Most likely, such microenvironment facilitates the Michael addition reaction and stabilizes the resulting adduct, consistently with previous proposals (Dennehy et al., 2006). For proteins with more than one reactive Cys residue, the obtained docking scores were able to discriminate between the primary ACR binding site and secondary sites modified only at high ACR concentrations. Therefore, docking scores emerge as a potential filter to predict Cys reactivity against acrylamide. Finally, inspection of the ACR-protein complex, combined with available experimental information, provided insights into the putative functional consequences of ACR modification, especially for non-enzyme proteins, for which *in vitro* or cellular assays assessing the impact of covalent adduct formation may not be readily available. Therefore, we expect that application of covalent docking to other proteins proposed to be targeted by ACR will help discern the most likely Cys reactive site and unravel the functional consequences of ACR adduct formation.

## 2 Methods

**FIGURE 2:**
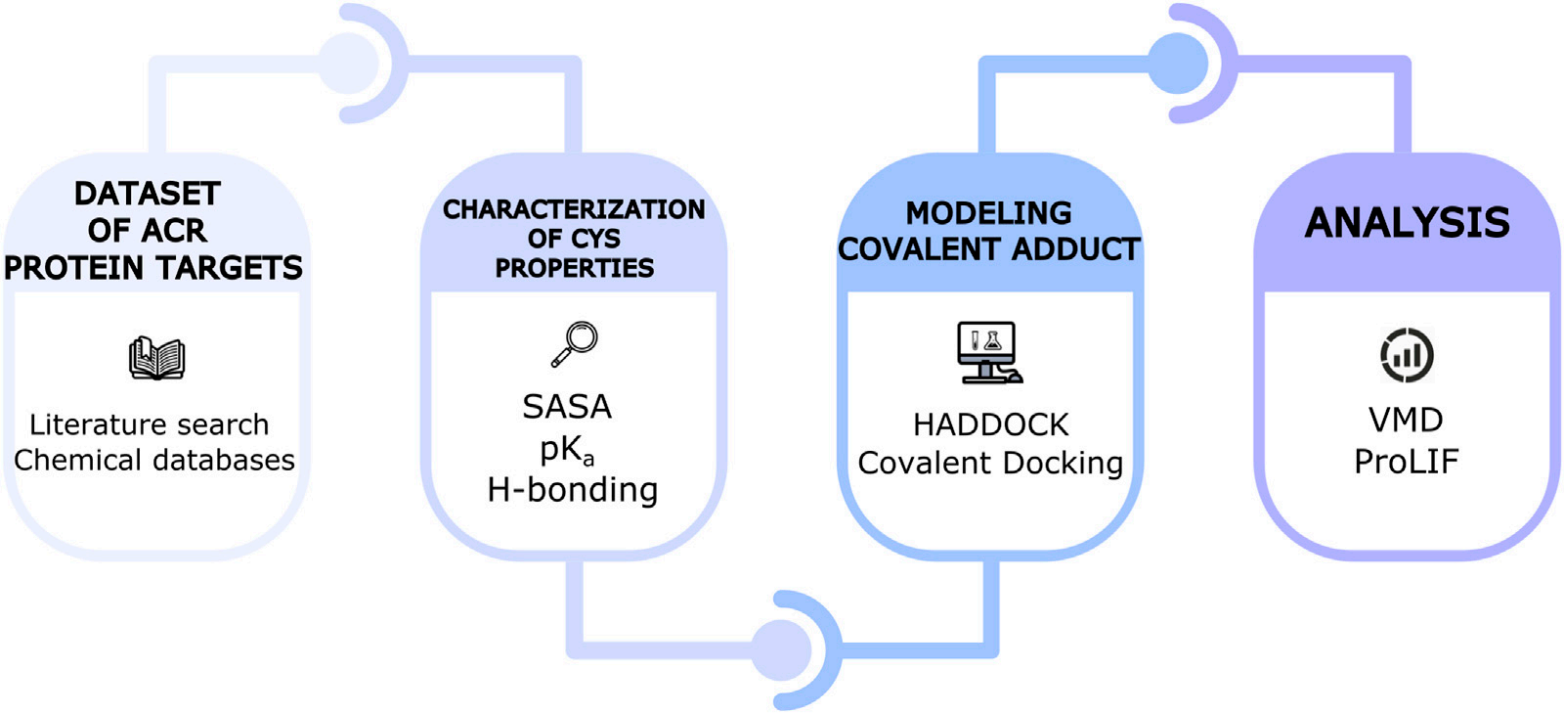
Schematic representation of the computational workflow used in this study.

Figure 2 shows the workflow of the present systematic study, which is further explained in the next sections.

### 2.1 Generation of the dataset of acrylamide protein targets

We first searched in the literature for human proteins targeted by acrylamide that have been associated to neurotoxic effects (Ferreira de Lima and Carloni, 2011; Papamokos et al., 2014) or used to biomonitor ACR exposure (Barber et al., 2001; Basile et al., 2008). In addition, we also included proteins listed as acrylamide targets in several chemical and toxicology databases, namely ChEMBL (Davies et al., 2015; Mendez et al., 2018) and T3DB (Lim et al., 2009; Wishart et al., 2015), respectively. Our dataset contains 19 proteins; the full list is given in Supplementary Tables S1–S2.

### 2.2 Characterization of cysteine properties

For eight proteins in our dataset, the location of the cysteine residue modified by acrylamide or by closely related electrophilic agents, such as N-ethylmaleimide (NEM), is known (LoPachin and Gavin, 2012). For the other 11 proteins, we employed physicochemical, conservation and functional data to rank the cysteines most likely to react with acrylamide (see Supplementary Material S1). For all the Cys residues present in dataset proteins, we calculated their solvent-accessible surface area (SASA, using the corresponding tool in VMD (Humphrey et al., 1996) with a probe of radius 1.4 Å), as well as predicted their pK_a_ values (using the H++ webserver (Anandakrishnan et al., 2012) with default settings, in particular assuming pH 7). Those Cys residues that are more solvent exposed and/or have more acidic pK_a_ values are expected to be more reactive (Marino and Gladyshev, 2011). The resulting SASA and pK_a_ values are listed in Supplementary Table S1. Moreover, we inspected UniProt (The UniProt Consortium, 2019) entries in search for functional annotations regarding candidate Cys residues for all proteins within the dataset. On one hand, Cys residues subject to redox post-translational modifications (e.g. sulfoxidation or S-nitrosylation) indicate reactive cysteines whose modification regulates protein function (Marino et al., 2013). On the other, Cys natural variants or Cys site-directed mutants that affect protein function can suggest a link between protein inactivation and acrylamide modification. The corresponding information is included in Supplementary Material S2. Complementary, we also considered whether the structural location of the candidate Cys is near a functionally relevant site of the protein (e.g. an enzyme active site); this information is listed in Supplementary Table S1. Further details are provided in the Supplementary Material S1.

### 2.3 Modeling of the covalent acrylamide-protein complexes

Acrylamide binding to cysteines of the proteins in Supplementary Table S1 was investigated with a covalent docking approach.

#### 2.3.1 Ligand and protein structures

The product of the corresponding Michael addition reaction, i.e. propionamide (Figure 1), was used as ligand. The respective 3D structure was obtained from PubChem (Sunghwan et al., 2019) (CID 6578).

The 3D structures of the human proteins in Supplementary Table S1 were taken from the Protein Data Bank (Berman et al., 2000; Burley et al., 2021). When more than one structure was available, the one at the highest resolution was chosen (see Supplementary Table S1). Protein structures with missing residues were retrieved from the SWISS-MODEL repository (Bienert et al., 2017) or generated with SWISS-MODEL (Waterhouse et al., 2018), by selecting templates structures with the same sequence as the targets. When experimental structures of the human protein were not available, we generated homology models (see Sections 3.2.4–3.2.7). Target-template sequence alignments were obtained with either BLAST (Camacho et al., 2009) or HHblits (Steinegger et al., 2019), as implemented in the SWISS-MODEL webserver. Templates with the highest sequence identity and the highest resolution were selected and models were generated with SWISS-MODEL (Waterhouse et al., 2018) (see Supplementary Table S1). Protein structures were processed with MolProbity (Chen et al., 2010; Williams et al., 2018) to add missing hydrogen atoms, assign histidine protonation states and perform His/Gln/Asn flips, if recommended. The reactive cysteines were modeled as already deprotonated (Foloppe et al., 2001), as expected for the Michael addition reaction to take place (see Figure 1, step 2). Therefore, our computational protocol does not take into account the energetic cost of Cys deprotonation, i.e. Δ*G =* ln*(10)* × *kT* × *(pK*_*a*_ − *pH)*. Moreover, we have assumed a default pH of 7, even though the protein targets in our dataset exhibit different optimal pH ranges (see Supplementary Table S3) and the Michael addition reaction is favored at basic pH (Lutolf et al., 2001; LoPachin et al., 2007; Nair et al., 2014). However, even if the Cys pK_a_ (calculated here with the H++ webserver at a default pH of 7) may predict population of the thiolate state smaller than the thiol one, reaction with acrylamide is expected to shift the acid-base equilibrium (step 2 in Figure 1) towards the deprotonated form.

#### 2.3.2 Covalent docking protocol

Covalent docking to the reactive cysteine(s) of each target protein was performed using Haddock (version 2.2.) (De Vries et al., 2010; van Zundert et al., 2016). We followed the standard covalent docking protocol of Haddock (HADDOCK developer team, 2018). Such protocol was initially tested for covalent inhibitors of cathepsin K (HADDOCK developer team, 2018) and here we have validated it using experimental protein structures containing Cys-ACR covalent adducts (see Section 1.2 in Supplementary Material S1). The covalent bond between Cys and the ligand is modeled by scaling down the van der Waals radius of the Cys sulfur atom 10-fold and introducing two distance restraints: (i) between the sulfur atom of the targeted cysteine and the reactive carbon atom of the ligand, set to 1.8 ± 0.1 Å (i.e. the average length of a single C-S bond) and (ii) between the cysteine C_*β*_ atom and the ligand carbon atom adjacent to the reactive carbon, set to 2.8 ± 0.1 Å (i.e. the same as between the C_*γ*_ and C_*ϵ*_ atoms of methionine, to model the proper angular geometry). The docking procedure (Kurkcuoglu et al., 2018; Koukos et al., 2019) consisted in the following three different stages: (1) A rigid body docking was performed with all geometrical parameters treated as fixed and allowing 180° rotations to generate 1,000 initial poses. After minimization, the best scored 200 poses were selected for further refinement. (2) A semi-flexible simulated annealing simulation (SA) in torsion angle space was applied to introduce gradually flexibility to the system. SA can be further divided into three steps. (2a) First, a rigid body simulated annealing was performed to optimize orientations of the interacting partners. (2b) Then, the system underwent 1000 molecular dynamics (MD) steps from 500K to 50K, with a 2 fs timestep, in which ligand and protein side chain movement was allowed. (2c) Finally, flexibility was introduced to both protein side chains and backbone, besides the ligand. 1000 MD steps (with a 2 fs timestep) were performed with a stepwise temperature decrement from 300K to 50K. It should be noted that flexibility was only applied to the ligand and protein residues within a range of 5 Å. (3) The final stage of the docking protocol was a refinement in explicit water. Namely, three MD-based steps (with a 2 fs timestep) were carried out: (3a) A heating phase of 100 MD steps from 100K to 200K and to 300K, (3b) 1250 MD steps at a constant temperature of 300K, and (3c) a cooling down phase of 500 MD steps to a final temperature of 100K. In stage (3), both ligand and protein were fully flexible, with the exception of protein backbone atoms. The HADDOCK score settings recommended for small molecule docking were used across the whole protocol (Kurkcuoglu et al., 2018; Koukos et al., 2019). The obtained 200 docking poses were clustered based on their positional protein-ligand interface root-mean-square deviation (iL-RMSD) with a cutoff of 1.0 Å. The Haddock score of each cluster was calculated as the average of the top four structures, as done by the Haddock webserver (De Vries et al., 2010; van Zundert et al., 2016), using the equation: *HADDOCK score = 1.0 Evdw + 0.1 Eelec + 1.0 Edesol + 0.1 Eair*, where *Evdw* and *Eelec* are the van der Waals and electrostatic intermolecular energies, respectively, *Edesolv* is the desolvation energy and *Eair* is the distance restraints energy; the weights of the different terms were parameterized for scoring of protein-ligand complexes (Kurkcuoglu et al., 2018; Koukos et al., 2019). Further analysis was performed for the top cluster (i.e. the one with the best average Haddock score) and, if present, also for other clusters with Haddock scores within the standard deviation of the top cluster. For proteins with more than one reactive cysteine, we performed independent dockings for each of the Cys residues. This approximation is valid provided that these cysteines are far enough apart that they (or their ACR covalent adducts) cannot interact with each other. However, in the case of one of the target proteins, creatine kinase (Supplementary Table S1), the two Cys are within 7.1 Å (C_*α*_-C_*α*_ distance) and thus we also considered the possibility that the two Cys could be targeted simultaneously by ACR (see Section 3.2.2). In this case, binding of two ligand molecules at the same time was modeled using the multibody docking approach (Karaca et al., 2010) implemented in HADDOCK. Namely, a so-called molecule interaction matrix is used to define partners that interact with each other. In particular, we defined the subsequent interacting pairs: protein-ACR molecule 1, protein-ACR molecule two and ACR molecule 1-ACR molecule 2.

### 2.4 Structure-based analysis of the modeled acrylamide-protein complexes

Hydrophobic interactions were investigated using VMD (Humphrey et al., 1996) (version 1.9.3.) and in-house scripts. Namely, such contacts were defined as interactions between either of the two carbon atoms of the ligand and “apolar” protein carbon atoms (i.e. with CHARMM-based point charges below 0.15 electrons) located within the distance cutoff of 4.0 Å. The hydrogen bond (HB) interactions with both the amide and the carbonyl group of the ligand were analyzed with ProLIF (Bouysset and Fiorucci, 2021) (version 1.0.0). The donor-acceptor distance cutoff was set to 4.1Å and the donor-hydrogen-acceptor angle tolerance to at least 100°. Each docking was analyzed separately, regardless of whether the reactive cysteines belong to the same protein or different protein targets. The protein-ligand interaction frequency is calculated as the percentage of poses belonging to the top (best scored) cluster that exhibit such interaction. When additional clusters with HADDOCK scores within the standard deviation of the top cluster are present, their poses were also included in the analysis, but a weighted average of the interaction frequencies was calculated, based on the size of each of the clusters analyzed. 2D representations of the protein-ligand interactions for each of the docking clusters considered were generated using ProLIF (Bouysset and Fiorucci, 2021) (version 1.0.0) and are shown in the Supplementary Material S1.

The covalent docking approach used here aims at predicting the most likely configuration or binding pose of the Cys-acrylamide adduct. However, the Michael addition reaction starts with the deprotonation of the reactive Cys. Hydrogen bonding to the Cys sulfur atom is crucial for thiolate formation and stabilization of the transition state of the subsequent reaction (Mazmanian et al., 2016). Moreover, the Michael addition reaction involves an enolate-type intermediate in which the ligand oxygen atom acquires negative charge (see step 2 in Figure 1) and thus hydrogen bonding or a positively charged microenvironment could stabilize this intermediate, facilitating adduct formation (Ha et al., 2011; Weber et al., 2013). Hence, we additionally analyzed protein residues either near the reactive Cys (in the initial X-ray structure of the protein target, i.e. before the Michael addition reaction occurs) or ligand (in the best structure of the top docking cluster, i.e. after covalent adduct formation). First, we checked H-bonded protein residues. These could act as potential proton acceptors to deprotonate the Cys sulfur atom or may stabilize the intermediate and/or product of the Michael addition reaction. Next, we visually inspected other nearby protein residues in the binding cavity that could have favorable, yet longer-range, electrostatic effects on thiolate or adduct formation. In particular, we focused on His, Asp, Glu, Arg and Lys. Histidine is one of the most interesting residues regarding acid-base properties, since its intrinsic pK_a_ of ∼6 is the closest value to the physiological pH of around 7, as well as to the intrinsic pK_a_ of Cys of ∼8.6. Hence, the imidazole side chain can be either singly or doubly protonated and thus serve as both proton acceptor and as positively charged residue stabilizing the thiolate formed upon Cys deprotonation. Aspartic and glutamic acids have lower intrinsic pK_a_ values (∼4.0 and ∼4.4); however, their pK_a_ can shift to higher values depending on their microenvironment. Hence, Asp and Glu can also be potentially responsible for Cys deprotonation in some cases. Instead, the positively charged Lys and Arg are expected to stabilize the negatively charged thiolate (or the enolate-type intermediate formed during the Michael addition reaction), either by forming a salt bridge or electrostatically. The results of this analysis of the Cys microenvironment are presented in Supplementary Table S2.

## 3 Results

### 3.1 Dataset of acrylamide protein targets

Our literature and chemical database search (see Section 2.1) rendered a total of 19 proteins modified by acrylamide that have been experimentally validated to mediate ACR (neuro)toxicity or to correlate with ACR exposure as biomarkers. The full list, together with additional protein and candidate Cys information, is given in Supplementary Tables S1-S3. The precise Cys modified by ACR (or closely related sulfhydryl agents, such as NEM) is known for eight of these protein targets and is indicated in bold in Supplementary Table S1. Noteworthily, incubation with high ACR concentrations and/or for longer times can result in more than one Cys being modified for some protein targets (e.g., glyceraldehyde-3-phosphate dehydrogenase and hemoglobin).

**FIGURE 3:**
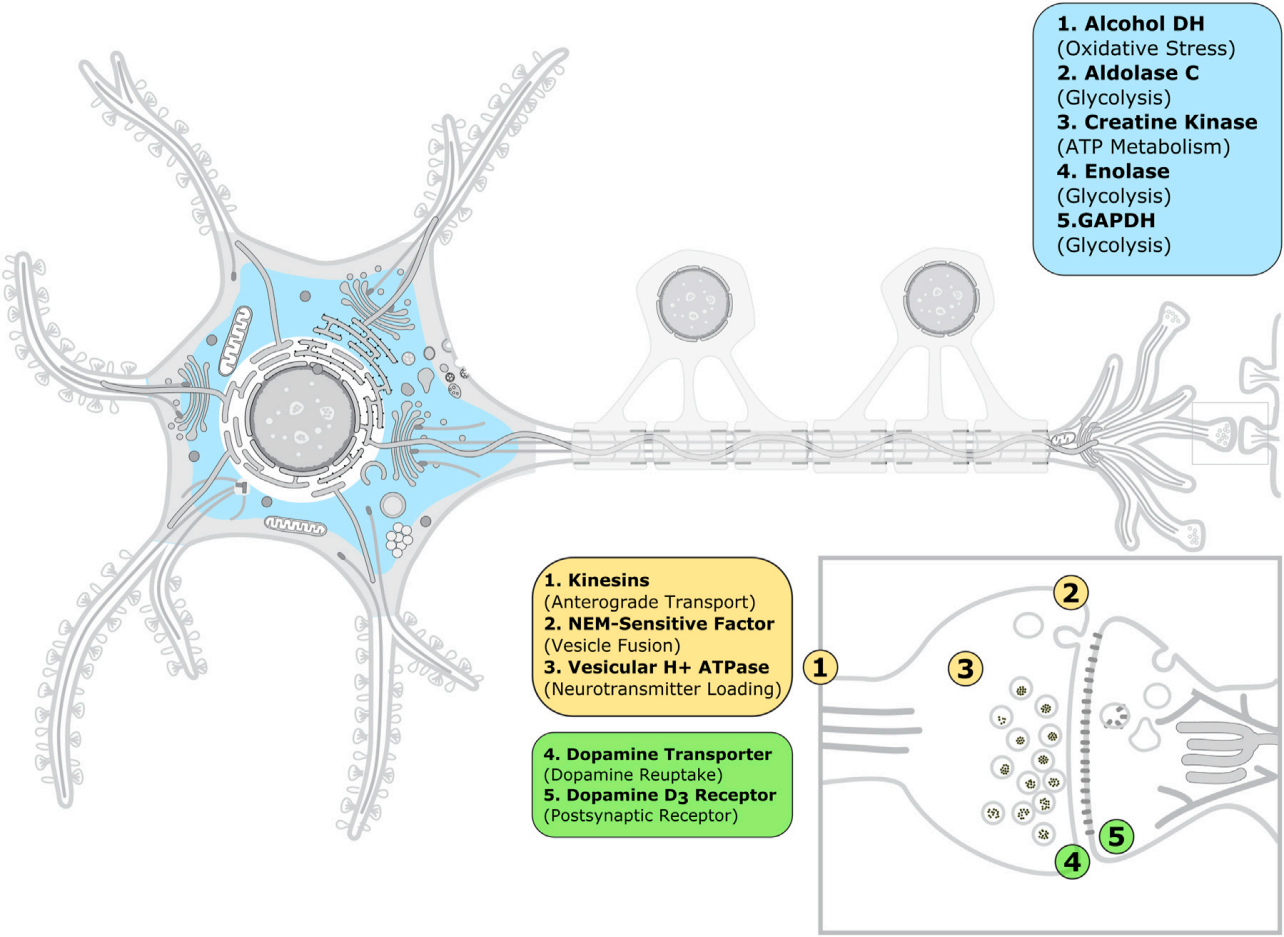
Subcellular location of the acrylamide protein targets associated to neurotoxicity. The image of the animal neuron cell and synapse was taken and adapted from SwissBioPics (Philippe et al., 2022) under a CC BY 4.0 license. The color code is as follows: blue, enzymes; yellow, ATPases; and green, membrane receptors and transporters. The enzymes shown can be present not only in the cytoplasm, but also the extracellular medium, with the exception of alcohol dehydrogenase.

Because experimental assays showing ACR-mediated inhibition are often performed *in vitro* using purified proteins, we analyzed next the functional classification and subcellular location of the proteins in our dataset in order to make a connection with the ACR toxic effects observed at the neuronal and systemic levels. Functionally, the ACR protein targets include enzymes (37%), ATPases (21%) and membrane receptors and transporters (16%), as well as plasma proteins (26%); see Supplementary Table S3; Supplementary Figure S3. We speculate that the predominance of enzymes and ATPases is due to the easier availability of purification protocols and functional assays to test ACR-mediated inhibition for these protein classes compared to membrane proteins. Given the diversity of protein classes targeted by ACR, we surmise that several subcellular mechanisms might contribute to acrylamide toxicity. At the neuronal level, ACR-mediated inhibition of cytosolic and extracellular enzymes (in blue in Figure 3) will cause metabolic imbalance and oxidative stress. In addition, ACR-mediated impaired function of synaptic proteins (ATPases and membrane proteins in yellow and green, respectively, in Figure 3) will result in inhibition of fast axonal transport (through kinesins), alteration of neurotransmitter levels (dopamine transporter, NEM-sensitive factor and vesicular proton ATPase) and direct inhibition of neurotransmission (dopamine D3 receptor). Therefore, our dataset contains synaptic proteins related to each of the three subcellular mechanisms proposed in reference (Erkekoglu and Baydar, 2014) to explain ACR neurotoxicity, even though the selection of proteins to include in our dataset was only based on experimental evidence of ACR modification. Among plasma proteins, the levels of ACR-modified albumin and hemoglobin have been used to monitor ACR exposure (Barber et al., 2001; Noort and Hulst, 2003; Basile et al., 2008).

The physiological relevance of ACR modification of the remaining eight proteins in our dataset is less clear. In addition to the genotoxic effects caused by direct reactivity of ACR and its metabolite glycidamide with DNA (Galesa et al., 2008; European Food Safety Authority (EFSA) et al., 2022), ACR-mediated inhibition of DNA topoisomerase and kinesins, as well as of estrogen receptor and sex hormone binding globulin, could further promote carcinogenicity and reproductive toxicity. In the case of the immunoglobulins in our dataset (Supplementary Table S1), we speculate that their ACR-mediated impairment may contribute to the observed ACR immunotoxicity and generation of reactive oxygen species (Kumar et al., 2018).

Interestingly, ACR modification of plasma proteins involves targets with only one free cysteine (for albumin) or cysteines usually involved in disulfide bonds (for sex hormone binding globulin and immunoglobulins, see Supplementary Tables S1-S2). In the latter case, reaction with ACR would require the existence of (at least a small) population of Cys in the free state, besides the disulfide bond-forming one. We surmise that ACR modification would then shift the equilibrium between the two redox states. In this regard, free thiols and chemical modification of disulfide bridges have been experimentally detected for immunoglobulins (Liu and May 2012).

### 3.2 Acrylamide protein targets with known reactive cysteine

The effects of ACR modification on the 19 proteins in our dataset were further investigated using covalent docking. Considering that some of the ACR protein targets have more than one potential reactive Cys (Supplementary Table S1), 34 covalent docking calculations were performed, following the protocol described in Section 2.3). Below we present the results for the eight ACR protein targets for which the reactive Cys is known (see Table 1), following the alphabetical order of the protein name. For the remaining eleven protein targets, detailed discussion can be found in the next section and in Supplementary Material S1. In all cases, we combined our computational results with previously published experimental data to surmise the possible functional consequences of ACR modification.

**TABLE 1:** Covalent docking results for the subset of acrylamide protein targets with experimentally known reactive cysteine. For each considered Cys, the Haddock score and size of the top docking cluster are shown. The latter corresponds to the number of docking poses belonging to the top cluster upon clustering of the total 200 poses.

Protein name		Reactive Cys	Score (a.u.)	Size
*Albumin*		C34	−32.3	63
*Creatine Kinase*		C74	−21.3	12
		C141	−31.7	63
		C146	−28.2	22
		C254	−23.3	34
		C283	−32.8	61
*Dopamine D3 Receptor*		C114	−28.6	130
*Dopamine Transporter*	*outward*	C342	−2.0	21
	*inward*	C135	−11.9	79
		C342	−15.1	87
*Glyceraldehyde-3-phosphate dehydrogenase*		C152	−12.3	163
		C156	46.1	1
		C247	9.9	14
*Hemoglobin*		C93	−13.9	74
		C104	−3.3	75
*NEM-sensitive factor*		C264	−73.4	85
*Vesicular proton ATPase*		C254	−0.1	110

#### 3.2.1 Human serum albumin (HSA)

**FIGURE 4:**
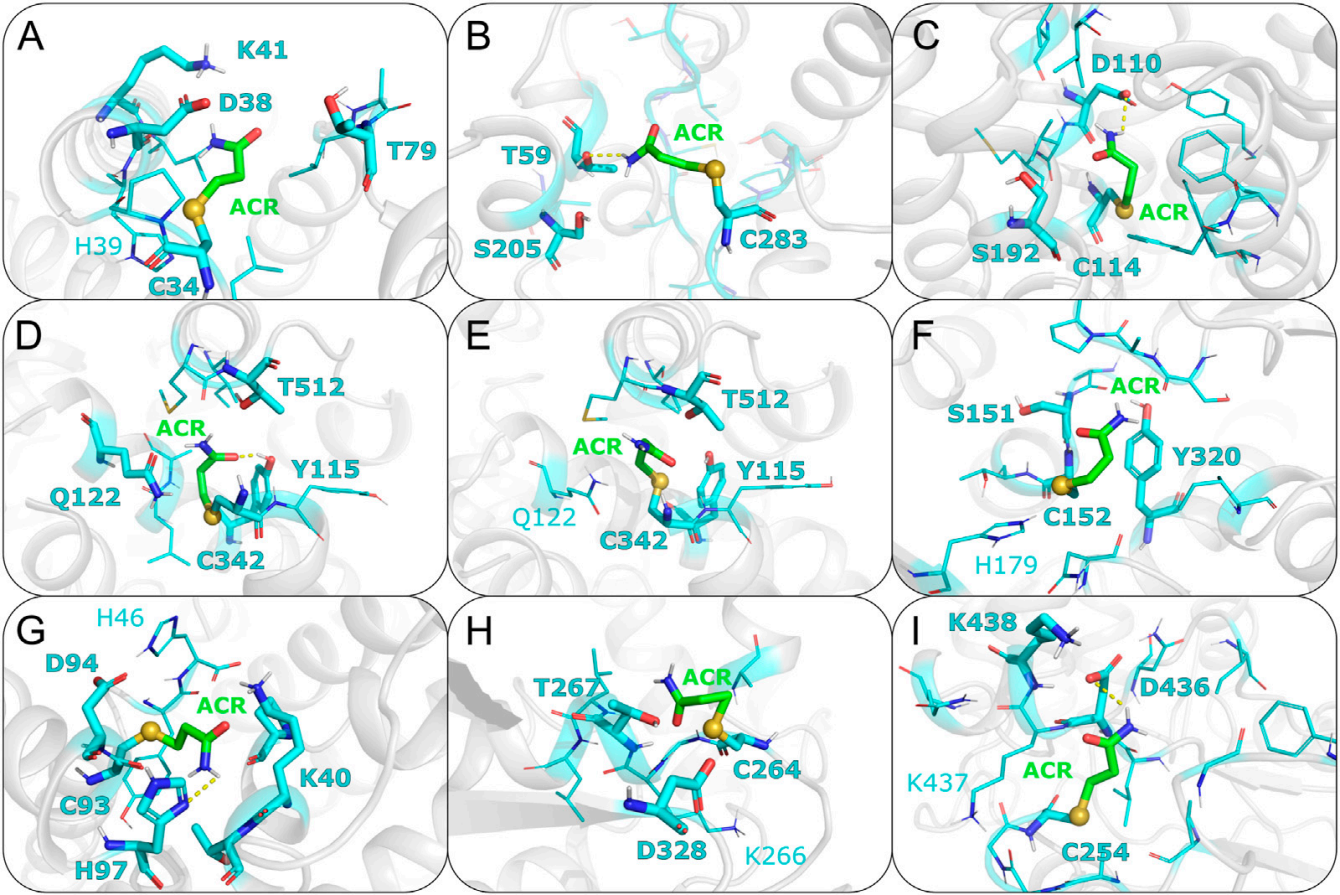
Representative covalent binding poses of ACR for each of the protein targets discussed in the main text. Acrylamide and its surrounding residues are represented as sticks, with carbon atoms colored in green and cyan, respectively. The sulfur atom between the reactive cysteine residue and the adduct is shown as a sphere. Residues forming hydrogen bonds (HBs) with ACR are displayed with thicker sticks and with bold labels. HBs present in more than 60% of the docking poses are shown with a dashed line. Nearby residues (i.e. within 5 Å) potentially favoring the Michael addition reaction are shown with thinner lines, with positively charged residues and putative proton acceptors labeled. **(A)** Albumin; **(B)** Creatine kinase; **(C)** Dopamine D3 receptor; **(D)** Dopamine transporter (inward conformation); **(E)** Dopamine transporter (outward conformation); **(F)** Glyceraldehyde-3-phosphate dehydrogenase; **(G)** Hemoglobin; **(H)** NEM-sensitive factor; **(I)** Vesicular proton ATPase.

Albumin is a plasma protein able to bind chemically diverse ligands, from hemin and fatty acids to drugs, acting as their plasma carrier/transporter (Fasano et al., 2005). Liquid chromatography–tandem mass spectrometry (LC-tandem MS) experiments have shown that C34 binds covalently acrylamide (Noort and Hulst, 2003; Tong et al., 2004). HSA contains 35 cysteine residues and all form disulfide bridges except C34 (Carter and Ho, 1994). This single free Cys is solvent exposed, with a SASA value of 8.6Å^2^, and has a calculated pK_a_ value of 10.2 (see Supplementary Table S1). This is in line with spectroscopic measurements showing HSA Cys34 to be more acidic than a normal Cys, with a pK_a_ around 7 (Pedersen and Jacobsen, 1980). The difference between the computational and experimental pK_a_ values can be ascribed to the known limitations of computational pK_a_ predictors when dealing with Cys residues (Awoonor-Williams and Rowley, 2016), as well as uncertainties in the experimental estimation of pK_a_ values using spectroscopic methods. For instance, the pK_a_ of Cys34 changes by 1.5 pH units depending on the ionic strength of the buffer used (Pedersen and Jacobsen, 1980). Covalent docking of ACR to C34 resulted in two similar clusters in terms of both score (−31.3 and −32.3 a.u., respectively) and cluster size (69 and 63, see Table 1). Mapping of C34 onto the HSA structure also revealed that this cysteine has two putative proton acceptors in the vicinity (H39 and D38) that can deprotonate the thiol group, as well as a positively charged residue (K41) that could stabilize the transition state and/or product of that reaction (see Figure 4A). Comparison with available functional information (Sampath et al., 2001; Fasano et al., 2005) suggests that acrylamide covalent binding to Cys34 might affect the drug binding properties of albumin. In particular, infrared spectroscopy has shown that Cys34 is linked allosterically with Sudlow’s site I for anesthetics such as halothane, propofol and chloroform (Sampath et al., 2001). Hence, formation of a covalent adduct at Cys34 can be transmitted to this site and modulate anesthetic binding.

#### 3.2.2 Creatine kinase (CK)

Creatine kinase is an enzyme responsible for converting creatine to phosphocreatine reversibly using adenosine triphosphate (ATP). CK is inhibited by acrylamide and such inhibition exhibits a biphasic behavior with respect to acrylamide concentration (Sheng et al., 2009), suggesting than more than one Cys residue within CK might be modified. C283 has been proposed as the primary site of ACR modification in CK. Based on site-directed mutagenesis, C283 was shown to be essential for enzymatic activity (Lin et al., 1994). Furthermore, experimental, studies indicated that C283 has a pK_a_ around 5.7 and thus this cysteine can be present as thiolate. This is probably required to constrain the position of the guanidinium group of the creatine substrate (Wang et al., 2006). The low pKa of C283 and its role in CK enzymatic activity makes it a good candidate for the main reactive Cys targeted by acrylamide. In contrast, the secondary site of ACR modification is unclear. A combined experimental and computational study (Sheng et al., 2009) suggested that acrylamide can bind to C283, as well as the nearby C74, but the results were not conclusive. Therefore, we performed docking for all five solvent exposed cysteines in CK (C74, C141, C146, C254 and C283; see Table 1). C283 is the most solvent exposed cysteine (37.2Å^2^) and with the lowest predicted pK_a_ value (around 9, see Supplementary Table S1). Although the calculated pK_a_ of C283 (∼ 9) differs from the experimentally measured value (5.7), we ascribed such difference to the known limitations of computational (implicit solvent-based) predictors when estimating the pK_a_ values of Cys residues, with RMSDs between 3.41 and 4.72 pKa units (Awoonor-Williams and Rowley, 2016). Given this uncertainty, we decided to use the calculated pK_a_ values only to rank by relative acidity the Cys residues within the same protein, i.e. C283 is the most acidic Cys in CK. In addition, the C283 docking yields the best score (−32.8 a.u.), as shown in Table 1. Furthermore, C283 is located in the catalytic site of CK, whereby nearby residues, such as R96 (distance of sulfur atom to *ζ*-carbon atom of 7.48Å), R132 (10.71Å) and R236 (10.70Å), can electrostatically stabilize the enolate intermediate of the Michael addition reaction (see Supplementary Table S3). Instead, docking at C74, previously proposed as acrylamide binding site (Sheng et al., 2009), gives a less favorable docking score (−21.3 a.u.). Together with most of the C74 poses showing distance values between the ligand C_*β*_ atom and sulfur atom outside the defined covalent bond range, this suggests that modification of C283 is preferred over binding to C74. Due to the proximity of C74 to C283, we also explored the possibility of two acrylamide molecules binding simultaneously to both C283 and C74, using a multibody docking approach (Karaca et al., 2010). The resulting docking poses indicate that adduct formation with one acrylamide molecule already occupies fully the pocket lined by C283 and C74 and thus will preclude binding of a second acrylamide molecule (see Supplementary Figure S12). Thus, C74 is unlikely to be modified by ACR, either alone or in combination with C283.

In contrast, docking to other cysteine residues revealed more suitable candidate for the secondary site of ACR modification in CK. The results for C141 and C146 yielded docking values closer to those of C283 (see Table 1), suggesting that modification of these two cysteines by acrylamide might be possible. Out of these two cysteines, C141 has a slightly more favorable docking score (−31.7 a.u.) than C146 (−28.2 a.u.), as well as a higher solvent exposed surface area (11.1 compared to 2.6Å^2^), suggesting a slightly higher preference of ACR for C141 over C146. Additionally, C141 has two nearby residues (H145 and E150) that could facilitate thiolate and/or adduct formation (see Supplementary Figures S6 and S7), whereas C146 is hydrogen bonded to P143 (see Supplementary Figures S6–S11). Based on our covalent docking results and the biphasic time dependent inactivation of CK observed in enzymatic assays (Sheng et al., 2009), we propose a molecular model in which ACR modification of C283 (Figure 4B) occurs first and is the primary site responsible for enzyme inactivation. Adduct formation at C283, located in the enzyme active site (Wang et al., 2006), will hinder creatine binding. At longer times, C141 might also be modified by ACR, further contributing to enzyme inactivation by thiol depletion (Meng et al., 2001; Lü et al., 2009).

#### 3.2.3 Dopamine D3 receptor (D3R)

Acrylamide exposure has been shown to result in decreasing dopamine concentrations by altering postsynaptic dopamine receptors (Erkekoglu and Baydar, 2014). Site-directed mutagenesis data showed that electrophilic compounds, such as NEM, blocked ligand binding to the dopamine D3 receptor (D3R) by modifying C114 (Alberts et al., 2009). Furthermore, functional data compiled in GPCRdb (Kooistra et al., 2020) indicates that C114 is involved in both ligand binding and receptor activation. Taken together the C114 reactivity and functional data, we considered C114 as the most likely candidate for acrylamide modification. Modeling of the covalent C114-ACR adduct further revealed how ACR modification can impair D3R signaling. The ligand interacts with D110 (Figure 4C); this aspartate is essential for ligand binding in aminergic GPCRs (Michino et al., 2015), such as D3R. Together with the aforementioned functional roles of C114 (Kooistra et al., 2020), this indicates that formation of the ACR covalent adduct will hinder ligand binding and/or impair receptor activation. Moreover, Cys at this position (3.36, following the Ballesteros-Weinstein generalized numbering for class A GPCRs) is conserved across dopamine receptors D2, D3 and D4. Given the role of these receptors in dopaminergic neurotransmission, ACR modification of Cys(3.36) might be one of the molecular mechanisms by which ACR intoxication mimics Parkinsonian symptoms.

#### 3.2.4 Dopamine transporter (DAT)

Dopamine transporters are integral membrane proteins responsible for regulating dopamine neurotransmitter concentrations at the synaptic cleft (Giros and Caron, 1993). Chemicals such as peroxynitrite and 2-aminoethyl methanethiosulfonate (MTSEA), which have in common the potential to modify cysteine sulfhydryls, are known to inhibit DAT (Park et al., 2002). Mutagenesis data has also shown that oxidation of C342 causes a decrease in DAT activity (Park et al., 2002). Furthermore, Cys modification is enhanced if the transporter is in the inward-facing state (Chen et al., 2000). To understand this differential reactivity of the two conformational states of DAT, we performed two covalent dockings for C342, using DAT structures in either outward- and inward-facing conformations (hereafter, OF and IF). Since experimental structural information for human DAT is missing, we generated homology models of the two transporter conformations. The templates used for the OF and IF models were the *Drosophila melanogaster* DAT (PDB code 6M2R) (Pidathala et al., 2021) and the human serotonin transporter (PDB code 6DZZ) (Coleman et al., 2019), respectively. The target-template sequence identities are 56.2% (OF) and 52.4% (IF); thus, the models are expected to be medium-to-high quality (Chothia and Lesk, 1986; Olivella et al., 2013; Piccoli et al., 2013). We further assessed the quality of the models by calculating their Ramachandran plots (Supplementary Figure S1) and QMEANbrane local quality values (Supplementary Figure S2). The percentage of residues in favored/allowed regions is 93.3%/98.7% (OF) and 95.6%/99.4% (IF), whereas the predicted local quality scores are above 0.7 (except for loop regions or not resolved in the template structures). Thus these two quality assessments support the reliability of the DAT homology models used here. SASA calculations show that C342 is more solvent exposed in the IF model, with SASA values four-fold larger than the OF model (see Supplementary Table S1). Therefore, cysteine accessibility seems to play a role in the observed higher reactivity of ACR with the IF state (Chen et al., 2000). Our covalent docking results (see Table 1) further support the enhanced ACR modification in the IF state. The top cluster for the IF model (Figure 4D) had a more favorable score of −15.1 a.u. than the one (−2.0 a.u.) for the OF model (Figure 4E). Such preferential binding of acrylamide to C342 in the IF state could alter the conformational transition between the two states crucial for dopamine transport, resulting in altered neurotransmitter concentrations. Considering the link between DAT and Parkinson’s disease, it is tempting to suggest that this might be responsible, at least in part, for the PD-like symptoms of acrylamide neurotoxicity (McHugh and Buckley, 2015; Jayaramayya et al., 2020).

Besides C342, we also performed covalent docking for C315. Mutagenesis experiments and transport assays upon treatment with sulfhydryl reagents have shown that C342 is the main modification site responsible for transport inhibition in wild-type DAT (Chen et al., 2000). However, C315 can also be modified and have a minor contribution to transport inhibition in a DAT C90A/C306A/C319F/C342A mutant construct lacking C342 (Whitehead et al., 2001), with higher C135 accessibility in the IF state (Chen et al., 2000; Whitehead et al., 2001). The docking score for this alternative C135 site (−11.9 a.u.) in the IF state is less favorable than for the main C342 site (−15.1 a.u.), further supporting our proposal that the docking score can help discriminate the most reactive Cys within a given protein target.

#### 3.2.5 Glyceraldehyde-3-phosphate dehydrogenase (GAPDH)

GAPDH is a housekeeping enzyme involved in both glycolysis, as well as apoptotic cell signaling. Multiple studies, both experimental and computational, have shown that acrylamide can covalently modify GAPDH, inhibiting enzymatic activity (Tanii and Hashimoto, 1985). Moreover, such enzymatic inactivation is concentration- and time-dependent, as well as pH sensitive Martyniuk et al. (2011). C152 has been identified as the most reactive cysteine compared to two other solvent exposed cysteine residues, C156 and C247 (Martyniuk et al., 2011). At small concentrations of acrylamide, almost only C152 is modified. As C152 is essential for GAPDH catalysis by acting as nucleophile, formation of the Michael adduct will result in enzyme inhibition (Martyniuk et al., 2011). However, at higher concentrations, ACR adducts with C156 and C247 are also formed and have been shown to further contribute to enzyme inhibition. To identify features that could explain this differential reactivity, we performed covalent docking for each of the aforementioned Cys residues (see Table 1; Figure 4F; Supplementary Figure S17–20). The top docking cluster for residue C152 had score of −12.3 a.u., significantly more favourable than those for C156 and C247 (46.1 and 9.9 a.u., respectively). Moreover, the last two dockings showed poses with C-S distances outside the covalent bond range. Therefore, modeling of the Michael adduct indicates that C152 is the primary binding site of ACR in GAPDH, in agreement with experiments (Martyniuk et al. 2011). In addition, both C156 and C247 had a calculated pK_a_ above 12, and thus are less likely to become deprotonated. Instead, the calculated pK_a_ value of 6.6 for C152 suggests that the sulfur atom can be present, at least partially, as a thiolate anion. Besides, C152 forms a hydrogen bond with residue H179 (Figure 4F); this could help deprotonate C152. Indeed, H179 activates the thiol group during enzymatic catalysis (Soukri et al., 1989). Moreover, the resulting doubly protonated H179 and its respective positive charge could electrostatically stabilize the enolate-type intermediate of the Michael addition reaction. Furthermore, the SASA values further support C152 as the most reactive residue, since its predicted accessibility is two orders of magnitude higher than C156 and C247 (see Table 1). Altogether, the computational results are in agreement with the experimental evidence that C152 is the primary site of acrylamide modification of GAPDH. Moreover, the higher reactivity of C152 with respect to C156 and C247 seems to correlate with the more favorable score obtained for the first cysteine.

#### 3.2.6 Hemoglobin (Hb)

Hemoglobin is a heme-containing protein responsible for oxygen transport from lungs to other tissues. Structurally, Hb is a heterotetramer (Ahmed et al., 2020) formed by two *α* and two *β* subunits that assemble as dimer of dimers (*α*_1_ *β*_1_ and *α*_2_ *β*_2_, respectively). Mass spectrometry showed that C93 within the *β* chains and C104 in the *α* chains are modified by acrylamide, with C93 being the most reactive site (Basile et al., 2008). Hence, measuring the levels of ACR-modified Hb in plasma can be used to monitor acrylamide exposure (Barber et al., 2001; Basile et al., 2008). The two aforementioned reactive cysteines have a predicted pK_a_ value above 12. However, C93 is more solvent-exposed compared to C104 (see Supplementary Table S1), suggesting that C93 is more accessible to acrylamide. This is in line with the covalent docking results obtained here (Table 1). The top docking cluster for C93 shows a significantly better docking score (−13.9 a.u.) compared to −3.3 a.u. for C104. Indeed, docking simulations with C104 did not result in a properly formed covalent bond between Cys and acrylamide, which could explain the less favorable docking scores compared to C93. Moreover, C93 has three nearby potential proton acceptors, H346, H97 and D294 (Figure 4G), belonging to the same *β*_2_ subunit. C93 is also located near K40 of the adjacent *α*_1_ chain, which stabilizes the adduct by forming a hydrogen bond with the ligand oxygen atom (see Supplementary Figures S21–S23). In addition, the nearby positively charged side chain could help stabilize the transient negative charge developed on the ligand oxygen atom during the nucleophilic attack. Instead, C104 only has a single nearby residue, H103, which could deprotonate the thiol group or stabilize the adduct. Taken together, our computational analysis suggests that C93 in the *β* subunit should be the primary site for acrylamide adduct formation in Hb, whereas binding to C104 in the *α* subunit is likely to occur only at higher acrylamide concentrations or longer times, in line with the experimentally observed reactivity (Basile et al., 2008). Moreover, the location of C93 at the interface between the *α*_1_ and *β*_2_ suggests that covalent modification of this cysteine by acrylamide could affect Hb function. Oxygen binding to Hb induces changes within this quartenary structure, i.e. a conformational transition from deoxyhemoglobin (T-state) to oxyhemoglobin (R-state). The largest movement occurs between the *α*_1_C-helix and the *β*_2_FG corner and a smaller change takes place between the *α*_1_FG corner and the *β*_2_C-helix (Perutz, 1979). Thus, the aforementioned *α*_1_ *β*_2_ intersubunit location of C93 might alter the transition from the T to R state triggered by oxygen binding to Hb and/or its cooperativity mechanism. We propose here that the effect of ACR modification on C93 could be tested experimentally, for instance, by measuring oxygen saturation at different oxygen partial pressures, after incubation with acrylamide. In the absence of such experimental validation, the functional impact of ACR modification is partially supported by a previous experimental study showing that covalent modification of C93 and C104 by other (larger) organic compounds prevented formation of the Hb tetramer (Hwang and Greer, 1980).

#### 3.2.7 NEM-sensitive factor (NSF)

N-ethylmaleimide(NEM)-sensitive factor is a homohexameric ATPase (Hoyle et al., 1996). In the presynaptic neuron, NSF, together with SNARE proteins, is involved in fusion of neurotransmitter-loaded vesicles with the cell membrane and vesicle recycling and thus is key for synaptic neurotransmission (May et al., 2001). Previous studies indicated that thiol reagents (e.g., NEM or NO) inhibit NSF (Matsushita et al., 2003). Mass spectrometry data showed that acrylamide modifies cysteine sulfhydryls, thereby altering the ATPase activity of NSF (Barber and LoPachin, 2004). Moreover, experimental evidence indicates that C264 is a critical residue for NSF function (Zhao et al., 2007). This cysteine is located in a so-called Walker A motif, important to ATP binding and thus for NSF ATPase function.

Due to the lack of experimental structural information for human NSF, we generated a homology model based on a *Cricetulus griseus* template (PDB code 3J94) (Zhao et al., 2015), which has sequence identity of 98.4%. Therefore, the model is expected to be high quality (Chothia and Lesk, 1986; Olivella et al., 2013; Piccoli et al., 2013). Indeed, its Ramanchandran plot (Supplementary Figure S1) shows 90.4%/96.5% residues in favored/allowed regions (comparable to the 93.0%/98.0% values, respectively, for the template structure, solved at 4.20Å resolution). Additionally, the QMEANDisCo local quality values (Supplementary Figure S2) are mostly above 0.7 (except for loop regions or not resolved in the template structures), further supporting the quality of the model. Our computational analysis using this homology model showed that, among the potential attachment points of ACR, C264 is both the most susceptible to deprotonation and the most accessible to acrylamide, with a pK_a_ value of 7.7 and a SASA value of 85.2Å^2^. The docking results further support the modification of C264 by acrylamide (Figure 4H). Structural inspection of the docking poses revealed that ACR would partially block access to the ATP binding site, thus hindering ATP binding and decreasing NSF activity.

#### 3.2.8 Vesicular proton ATPase (v-ATPase)

Filling of the synaptic vesicles with neurotransmitters relies on the proton gradient created by the vesicular proton ATPases (v-ATPases) using ATP. N-ethylmaleimide (NEM), a sulfhydryl reagent similar to ACR, reduces H^+^ uptake, as well as decreases v-ATPase activity (Shulamit and Sihra, 1989). The modification site responsible for v-ATPase inhibition by sulfhydryl reagents (Feng and Forgac, 1992; LoPachin and Barber, 2006) has been proposed to be C254, which is located in a loop segment of the v-ATPase catalytic subunit, corresponding to the so-called Walker A (GAFGCGKT) motif coordinating ATP binding and hydrolysis. Physicochemical characterization of C254 revealed that this cysteine has a pK_a_ value of 10.3 and a SASA value of 11.0 Å, further supporting this particular cysteine as ACR target site. Thus, we performed the corresponding Haddock calculation for C254. The covalent docking poses obtained here (Figure 4I) show that the ligand is placed at the entrance of the active site and thus can hinder ATP binding. Nonetheless, the loop where C254 is located exhibits large rearrangements during the conformational cycle of v-ATPase (see Supplementary Figure S28) and such structural changes cannot be modeled with covalent docking. Hence, we integrated additional experimental data for validation. Our hypothesis is indirectly supported by the experimental observation that NEM inactivation of v-ATPase is associated with exposure of a single cysteine residue that can be protected by incubation with nucleotides (Hunt and Sanders, 1996). Moreover, another experimental study showed that C254 modification, either through formation of a disulfide bridge with C532 or through adduct formation with NEM, causes inactivation of the v-ATPase (Feng and Forgac, 1994). Therefore, we surmise that modification of C254 by ACR might have a similar inhibitory effect.

### 3.3 Prediction of ACR-modifiable Cys candidates with limited experimental information

Besides performing dockings for ACR protein targets with experimentally validated reactive Cys (see previous Sections 3.2.1–3.2.8), we also considered protein targets that are known to be modified by ACR or NEM, but for which the specific cysteine(s) forming the covalent adduct is not known (see Supplementary Table S1). In order to pinpoint the most likely reactive Cys candidates, we first analyzed cysteine properties (SASA and pKa values), inspected their microenvironment (since nearby residues can favor Cys deprotonation) and checked for post-translational modifications and Cys conservation (see Section 2.2). Similar criteria have been previously used to predict cysteines potentially involved in disulfide bridges, metal binding, post-translational modifications or catalysis (as nucleophile) (Soylu and Marino, 2015, 2017; Li et al., 2021). For most proteins, this first filtering rendered multiple Cys residues potentially targeted by ACR. Hence, covalent docking was performed for each candidate Cys. Based on the observation that more favorable docking scores appear to correlate with Cys reactivity against ACR within a given protein (see e.g., Sections 3.2.2 and 3.2.5), we used the docking scores as second filter to suggest the most likely Cys to be modified by ACR (see Supplementary Table S1). We would like to emphasize that the Haddock scoring function used here (Kurkcuoglu et al., 2018; Koukos et al., 2019) is not normalized and thus the docking score is used only to predict ACR reactivity for Cys residues belonging to the same protein, but not to compare different proteins. Further details for this subset of proteins with no experimental information on the reactive cysteine(s) are provided in the Supplementary Material S1. The protein-ligand interaction fingerprints of the obtained docking poses are shown in Supplementary Material S1, whereas Supplementary Material S2 includes the average Haddock score and size of the docking clusters, as well as the individual scores for the top four structures of each cluster.

### 3.4 Hydrogen bonds

For each of the considered protein targets in Supplementary Table S1, we analyzed the hydrogen bond (H-bond) network between the ligand and its surrounding binding site residues. The representative clusters of each docking were pooled together and hydrogen bond frequencies were calculated as explained in the Methods Section 2.

**FIGURE 5:**
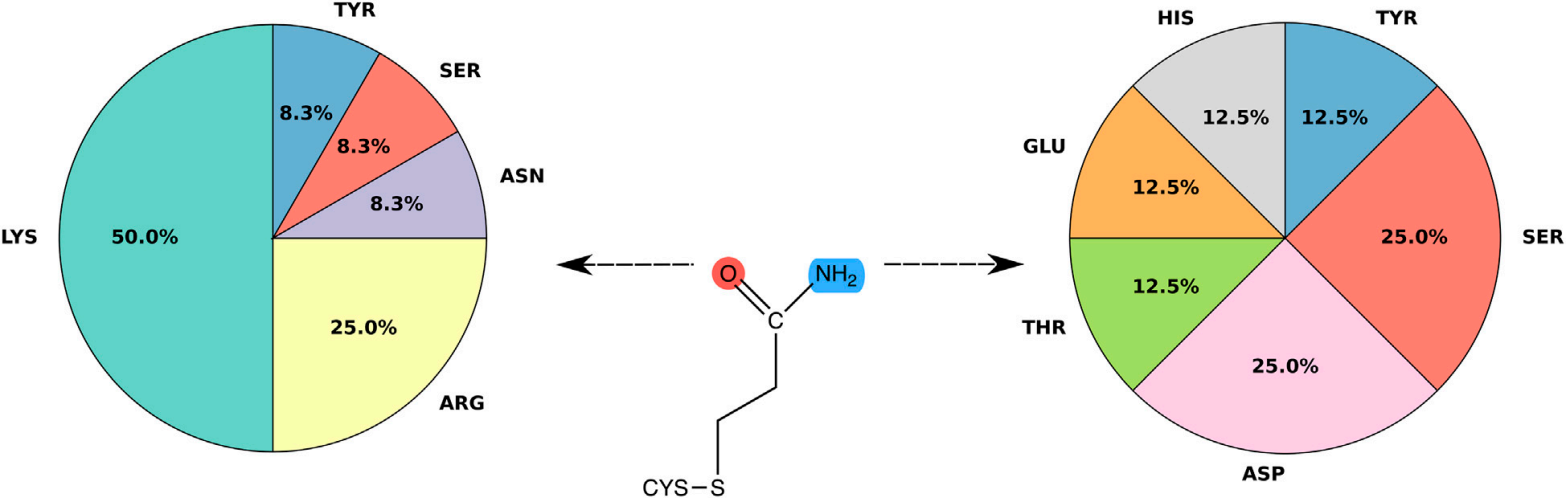
Frequency of hydrogen bonds. Pie chart showing the distribution of binding residues forming H-bonds with acrylamide. Only specific interactions with amino acid side chains and hydrogen bonds with frequency over 60% were considered.

Figure 5 shows ligand-protein H-bonds classified by type of amino acid. Only H-bonds with amino acid sidechains and frequency over 60% are displayed; the use of other thresholds turned out not to significantly change the amino acid ranking. Interactions were grouped based on whether the H-bond was formed with either the carbonyl or the amino group of the ligand. Among the H-bonds formed with the carbonyl group, lysine is the residue with the highest frequency (50.0%), followed by arginine (25.0%). After these positively charged residues, asparagine, serine and tyrosine are next in the ranking, with a frequency of 8.3% each. The ligand amino group formed instead H-bonds with polar residues, such as serine (20%), as well as histidine, tyrosine, aspartic acid, threonine and glutamic acid (14.3% each). By grouping amino acids of similar chemical characteristics a more clear picture emerges. In particular, positively charged amino acids, i.e. lysine and arginine, act as main H-bond donors to the carbonyl group of the covalent adduct and have a combined frequency of 75%. Therefore, our structure-based analysis suggests a preference of the ligand carbonyl group to interact with positively charged amino acids, in line with a previous sequence only-based analysis with other thiol-reactive electrophiles (Dennehy et al., 2006). The ligand amino group shows a more diverse picture, in that we did not observe any amino acid preference to interact with the amino group. In particular, the ligand nitrogen does not prefer to interact with negatively charged amino acids (aspartate and glutamate, see Figure 5). We surmise that the specificity of the H-bonds with the carbonyl group with respect to the amino group is a remnant of the role of the H-bond donors in the mechanism of the Michael addition reaction. H-bonding to the carbonyl group does not only contribute to stabilize the resulting Michael adduct, but can also help to stabilize the negative charge developed in the enolate-type reaction intermediate.

## 4 Discussion

Cysteine residues are one of the least frequent (3.3%) proteinogenic amino acids (King and Jukes, 1969). Nevertheless, this residue is disproportionately involved in a variety of important protein functions due to the nucleophilic and redox properties of the thiol group (e.g catalytic or regulatory activities), as well as its ability to form disulfide bridges and bind metal ions. In this regard, proteins within the synaptic vesicle cycle are of interest, since they are considered as cysteine-rich proteins (Calakos and Scheller, 1996).

Acrylamide (ACR) is a toxicant that has been shown to affect protein function by reacting with Cys residues of several protein targets (LoPachin and Barber, 2006; LoPachin et al., 2007, 2008; LoPachin and Gavin, 2012). Such covalent adduct formation proceeds through a Michael addition mechanism and requires the Cys thiol group to be accessible to acrylamide, as well as deprotonated (i.e. thiolate, see Figure 1, step 2), so that it can act as nucleophile (Figure 1, step 3).

Here, we first calculated Cys physicochemical properties to assess whether these intrinsic values would help pinpoint the most reactive cysteine(s) in a given ACR protein target. The solvent accessible surface area (SASA) is used as proxy of the Cys exposure to ligands. However, the SASA values can significantly vary depending on the resolution of the crystal structure (which can affect the accuracy of the position of the Cys side chain) and/or the functional state of the structure chosen (for the analysis for proteins undergoing large conformational changes during their functional cycle). For instance, the accessibility of C342 in DAT is larger in the IF state compared to OF (see Supplementary Table S1). As for the prediction of pK_a_ values, popular software packages, such as H++ (Anandakrishnan et al., 2012), PROPKA (Rostkowski et al., 2011) or MCCE2 (Song et al., 2009), are quite successful in predicting the acidity of aspartic or glutamic acid, but their performance for Cys residues is significantly lower (Awoonor-Williams and Rowley, 2016). For instance, C283 in CK has an experimentally validated pK_a_ value of ∼5.6 (Pahari et al., 2019), yet both H++ and PROPKA estimated values of around 9 (Wang et al., 2006). Unfortunately, both experimental and computational approaches show limitations at estimating pK_a_ values. Experimentally, NMR is the most commonly used technique for pK_a_ estimation; however, such experiments are quite demanding, in that they require recording multidimensional (^13^C and ^15^N) spectra at different pH values (Stivers et al., 1996; Czerwinski et al., 2001; Hass and Mulder, 2015). Hence, PKAD (a database of experimentally measured pK_a_ values of ionizable groups in proteins) (Pahari et al., 2019) contains data only for 1,350 residues in 157 wild-type proteins and for 232 residues in 45 mutant proteins; out of these, only 20 values correspond to Cys residues. Computationally, besides implicit solvent methods such as PROPKA (Rostkowski et al., 2011) and H++ (Anandakrishnan et al., 2012) mentioned here, explicit solvent methods include, among others, constant pH (Harris and Shen, 2019; Aho et al., 2022) or quantum mechanics/molecular mechanics (QM/MM) (Riccardi et al., 2005). Such molecular dynamics-based approaches have been shown to perform better at predicting pK_a_ values; however, their high computational cost makes them prohibitive for systematic applications, such as the one presented here. Moreover, even such advanced approaches may have limitations. For instance, convergence problems might arise due to limited sampling of the titratable residue conformations and of the reorganization of the surrounding protein environment and water molecules upon protonation state change. Moreover, in the case of QM/MM, estimating the energetic contribution of proton dissociation from the protein to the bulk solvent is far from trivial (Mangold et al., 2011; Borstnar et al., 2012; Wang et al., 2020). Therefore, in this work we decided to take advantage of a low computational cost approach such as the H++ webserver (Anandakrishnan et al., 2012) and then use the calculated pK_a_ values only to rank Cys residues within a given target protein by acidity, rather than considering the predicted absolute values. Moreover, as thiolate formation is favored in the presence of H-bonding and positively charged residues (Roos et al., 2013), we further inspected nearby residues in order to identify possible candidates to increase Cys acidity (such as Lys and Arg) or act as proton acceptors (such as His, Asp and Glu). However, this initial filtering of the candidate Cys residues based on physicochemical properties and microenvironment effects is not able to pinpoint a single ACR target site, but rather helps to discard the least likely Cys sites, as shown in Section 3.3. This is not surprising, because such analysis has been used to predict Cys reactivity in general (see e.g., (Soylu and Marino, 2015, 2017; Li et al., 2021)), but does not take into account specific features related to acrylamide reactivity.

In this work, we have compiled a list of protein targets associated to ACR toxicity and biomonitoring of ACR exposure and used a covalent docking approach to model the adduct formed upon Michael addition reaction of acrylamide with Cys residues of these proteins (Supplementary Tables S1–S3). First, we modeled the Cys-ACR covalent adduct for a set of protein targets for which the most reactive Cys is experimentally verified (i.e. those target proteins with a Cys highlighted in bold font in Supplementary Table S1). This is the case for (i) C34 of albumin, (ii) C283 of creatine kinase, (iii) C114 of dopamine D3 receptor, (iv) C342 of dopamine transporter, (v) C152 of GAPDH, (vi) C93 of hemoglobin, (vii) C264 of NSF and (viii) C254 of v-ATPase. The covalent docking approach used here, based on scaling down the van der Waals parameters of the Cys sulfur atom and defining two distance restraints, allows to streamline the generation of structural models of the protein-ACR adducts, compared to more computationally intensive approaches, such as QM/MM (Mondal and Warshel, 2020; Luo, 2021; Mihalovits et al., 2022). Moreover, in the case of creatine kinase, DAT, GAPDH and hemoglobin, we also applied covalent docking to secondary Cys sites shown experimentally to be modified at increasing ACR concentrations or longer incubation times (see Sections 3.2.2–3.2.6). We found that the docking score was able to discriminate between primary and secondary ACR sites of the aforementioned proteins, as well as the higher reactivity of C342 of the DAT in the IF state compared to the OF one (see Section 3.2.4). Therefore, we surmised that covalent docking scores can help identify the main Cys reacting with ACR within a given protein and proceeded to apply the same approach to other protein targets associated to ACR toxicity for which the reactive Cys is unknown (see Supplementary Table S1). Although the aforementioned traditional approaches (Soylu and Marino, 2015, 2017), based on solvent accessibility, pKa prediction and H-bonding environment, can help pinpoint possible reactive Cys, the combination with covalent docking, as proposed here, can help better discriminate between different cysteines within the protein. Based on our observation that higher Cys reactivity against ACR turned out to correlate with more favorable docking scores, we suggest that the following Cys are modified by ACR (marked with an asterisk in Supplementary Table S1): (i) C240 of alcohol dehydrogenase, (ii) C134, C239, C268 and C289 of aldolase, (iii), C134 of immunoglobulin kappa light chain, (iv) C398 of enolase, (v) C381 and C530 of estrogen receptor, (vi) C663 of kinesin KIF1C, (vii) C260 and C287 of kinesin KIF2C, (viii) C395 of immunoglobulin G1 H Nie, (ix) C997 of topoisomerase IIa, and (x) C164 and C188 of sex hormone-binding globulin. Nevertheless, further experiments are needed to validate our computational predictions.

Analysis of the residues surrounding the ACR covalent adducts modeled in the present study shows that acrylamide binding sites are enriched in positively charged Arg and Lys residues (see Figure 5). Thus, our structure-based study confirms the hypothesis put forward in a previous sequence only-based study with other thiol-reactive electrophiles (Dennehy et al., 2006). In addition, our work shows that residues such as His, Asp or Glu are often found in close proximity of ACR-modified Cys sites. We surmise that the particular amino acid composition of acrylamide binding sites may have catalytic effects on covalent adduct formation, in line with a previous study on model peptide systems (Lutolf et al., 2001; Nair et al., 2014). Cys deprotonation (step 2 in Figure 1) may be favored in the presence of positively charged Lys and Arg (which lower the Cys pKa) and His/Asp/Glu residues (which can either further decrease the Cys pKa by H-bonding or act as proton acceptors). Moreover, the Michael addition reaction (step 3 in Figure 1) proceeds *via* an enolate-type intermediate, which may be stabilized in the presence of Lys/Arg/His interacting with the negatively charged oxygen atom, thus decreasing the reaction energy barrier.

We would like to note here that, although the docking protocol used here (see Section 2.3.2) includes a final refinement step in the presence of explicit water molecules, our analysis of the protein-ligand H-bonds was focused on direct interactions only and thus does not include water-mediated interactions. However, water molecules could also play a catalytic role in acrylamide adduct formation. Water can participate in Cys deprotonation (step 2 in Figure 1), either as proton shuttle between the reactive Cys and a nearby proton acceptor residue or as a base itself. Unfortunately, such role is also difficult to predict on the basis of the experimental protein structures alone, as many show only a limited number of structured water molecules or lack water molecules altogether (Gnesia and Carugo, 2017; Caldararu et al., 2020). In addition, water can help further stabilize the covalent adduct (formed upon step 3 in Figure 1) by mediating H-bonds between the adduct and nearby residues, as observed in other protein-ligand complexes (Breiten et al., 2013). However, here we were interested in predicting the protein binding site determinants of acrylamide reactivity, rather than quantifying the binding energetics.

The covalent protocol used here has two potential limitations. As for the SASA and pK_a_ calculations, the docking results might depend on the input protein structures. To minimize this dependency, we chose the highest resolution structure available for each protein target (to minimize possible errors in the accuracy of the position of the Cys side chain) and employed a fully flexible docking approach (to allow the protein environment to adjust to the presence of the ACR adduct). Nonetheless, docking approaches cannot model large conformational changes. Therefore, analysis of proteins undergoing large structural rearrangements during their functional cycle might require previous knowledge on the conformational state preferentially targeted by ACR (as done here for the dopamine transporter, see Section 3.2.4). Moreover, classical docking scoring functions, such as the one used here (see Section 2.3.2), have been parameterized to describe noncovalent protein-ligand interactions and thus can have limited accuracy at describing covalent ligands (Scarpino et al., 2018; Sotriffer, 2018). Nonetheless, such approaches still exhibit a high success rate for certain warhead chemistry and ligand features, in particular the Michael addition reaction and small size ligands (Scarpino et al., 2018), as it is the case for the Cys-ACR adducts considered here.

In conclusion, the application of covalent docking to ACR protein targets has provided molecular insights into the binding site where the covalent adduct is formed upon Michael addition. Such sites are enriched in Lys and Arg residues and additionally contain H-bonding residues that stabilize the covalent adduct. Docking scores emerge as a predictive tool to pinpoint Cys residues most likely to be modified by ACR within a given protein. Therefore, the computational workflow presented here (Figure 2) could serve to filter putative ACR protein targets and candidate reactive Cys resulting from mass spectrometry-based proteomics studies and prioritize those that are more likely to be true positives. However, given the limitations of docking, such ranking should be used to guide follow-up validation studies. Mutagenesis and biochemical experiments would help to assess the impact of ACR on protein function and eventually (neuro)toxicity and computational simulations would provide further insights into the reaction mechanism of ACR modification, as done for other covalent inhibitors (Mondal and Warshel, 2020; Luo, 2021; Mihalovits et al., 2022).

The computational workflow presented here is based on experimental structures from the Protein Data Bank (Berman et al., 2000; Burley et al., 2021). However, recently developed machine learning-based protein structure prediction algorithms (Baek et al., 2021; Jumper et al., 2021; Ahdritz et al., 2022; Lin et al., 2022) could also be used to generate input protein structures. Moreover, here we performed the covalent docking calculations with HADDOCK (Dominguez et al., 2003), because its availability as a webserver (De Vries et al., 2010; van Zundert et al., 2016) and the minimal preparation of the protein structures required makes our workflow accessible to both new and experienced docking users. Nonetheless, processing the large number of possible candidate ACR protein targets and reactive Cys sites emerging from mass spectrometry-based proteomics will require automated covalent docking workflows, which could integrate either HADDOCK or other docking programs (Scarpino et al., 2018), such as GOLD (Borisek et al., 2015) or Schrödinger (Zhu et al., 2014). Upon such covalent docking-based initial screening, the most promising protein targets and reactive Cys sites could be further filtered and analyzed using more computationally intensive QM/MM methods (Luo, 2021; Mihalovits et al., 2022), such as empirical valence bond (Warshel and Levitt, 1976; Warshel, 1991).

## Data Availability

The original contributions presented in the study are included in the article/Supplementary Material; further inquiries can be directed to the corresponding author.
