# Supplementary material for: Molecular determinants of acrylamide neurotoxicity through covalent docking
Source: Front Pharmacol. 2023 Mar 2;14:1125871. doi: 10.3389/fphar.2023.1125871 (PMC10018202; doi:10.3389/fphar.2023.1125871)
Supplement: Supplementary file 1 [file DataSheet1.pdf]

Supplementary Material 1

1 SUPPLEMENTARY DATA

1.1 Dataset of acrylamide protein targets

The list of 19 proteins emerging from our literature and chemical database search of acrylamide (ACR) targets (see section 2.1 in the main text) is given in Table S1. Experimental structures are available for 17 out of the 19 target proteins in our dataset; when more than one structure was available, we chose the one at the highest resolution (see Table S1). For the two remaining targets (i.e. the dopamine transporter and the NEM-sensitive factor), we generated homology models of the human proteins, as explained in section 2.3.1 in the main text. Quality evaluation of these models is discussed in the main text (sections 3.2.4 and 3.2.7). In particular, Ramachandran plots were calculated to check whether the protein backbone geometry falls within allowed/favored regions; these plots are shown in Figure S1. In addition, we estimated the local quality values (Figure S2) using QMEANbrane for DAT and QMEANDisCo (Studer et al., 2020) for NSF. We would like to note here that QMEANbrane (Studer et al., 2014) has been trained to evaluate the quality of homology models for membrane proteins, such as the DAT, whereas QMEANDisCo (Studer et al., 2020) was developed for soluble proteins, such as the ATPase domain of NSF considered here.

For each protein target, we have used the structures listed in Table S1 to analyze the physicochemical properties and location for each of the candidate Cys residues, as well as their corresponding microenvironment, as explained in the main text (see section 2.4). Most of the cysteines in our dataset happened to be located in enzyme active sites (see Table S1). Most likely this reflects the more readily available purification methods and functional assays for enzymes compared to other protein functional classes (see also below). Cys residues with higher SASA and lower pK_a values in Table S1 are more accessible and acidic and thus potentially more reactive. However, SASA and pK_a calculations can have limited accuracy due to the dependency on the structure used to represent the protein and the poor performance of pK_a predictors for Cys (Awoonor-Williams and Rowley, 2016)). Hence, we have also inspected residues in the vicinity of the candidate cysteines that could potentially favor Cys deprotonation (step 2 in Figure 1 in the main text). His and Asp/Glu (in green and red, respectively, in Table S2) could deprotonate the Cys thiol group, whereas Arg/Lys (in blue) would stabilize the resulting thiolate. In addition, other H-bonding capable residues (in orange in Table S2) could also help make the Cys more acidic (Roos et al., 2013).

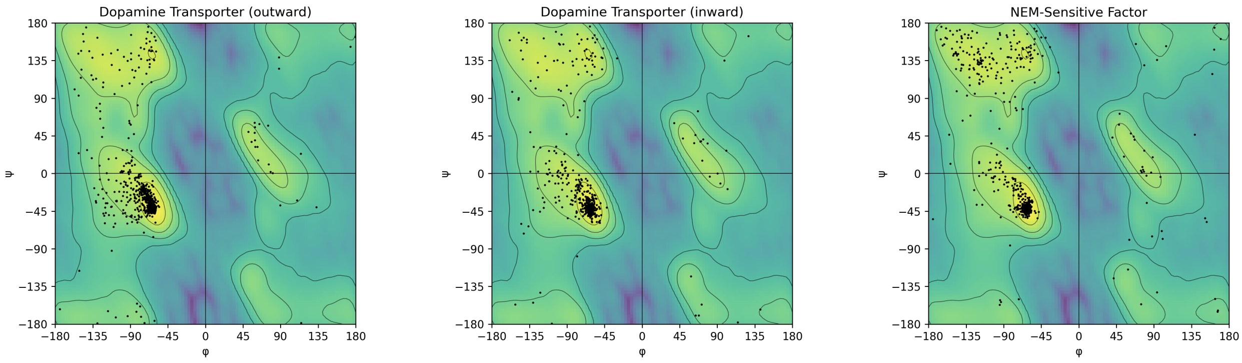

Figure S1: Ramachandran plots of the homology models built for the dopamine transporter, outward and inward conformations, as well as the NEM-sensitive factor (from left to right). The plots were generated using the RamachanDraw tool (https://pypi.org/project/RamachanDraw/), distributed under the MIT license.

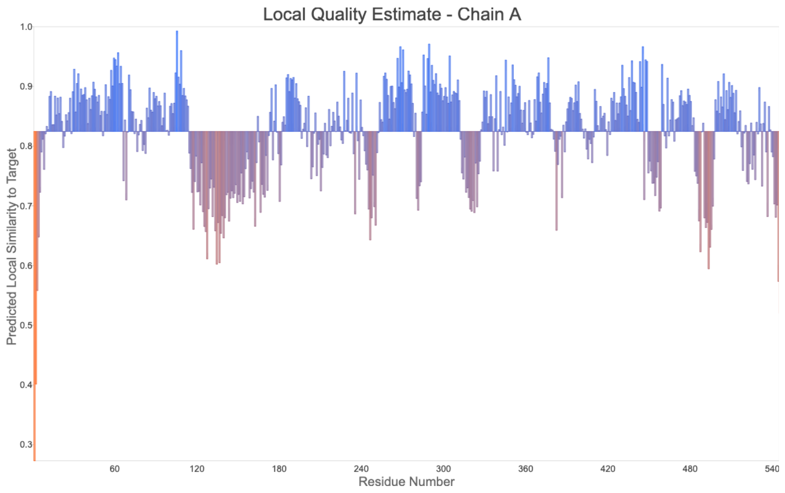

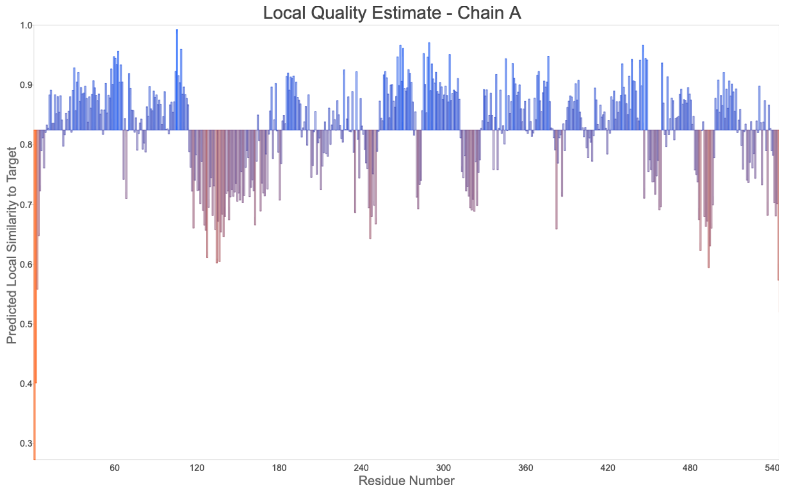

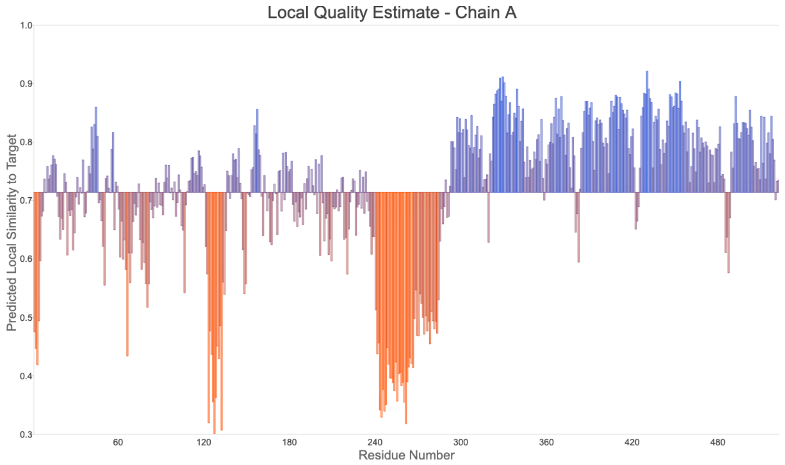

Figure S2: Local quality values of the homology models built for the dopamine transporter, outward and inward conformations, and the NEM-sensitive factor (from top to bottom). The plots were generated with the QMEAN webserver (https://swissmodel.expasy.org/qmean/).

Table S1: Acrylamide protein targets compiled in this study. Protein structures and cysteine residues considered for the covalent docking calculations are listed. Targets for which homology models of the human proteins had to be generated are indicated by HM and the template structure used between parentheses; further details are provided in the main text (sections 3.2.4 and 3.2.7). Physicochemical characterization and location of the candidate cysteines are also included; n.a. indicates cysteines for which no functionally relevant location was identified. Residue numbers highlighted in bold indicate the main reactive cysteine in protein targets with experimental data, whereas an asterisk marks residues predicted to be targeted by acrylamide based on the results of our covalent docking calculations.

#	Protein name	PDB code	Resolution (Å)	Cysteine	SASA (Å^2)	pK_a	Cys location	References
(1)	Albumin	6HSC	1.9	**34**	8.6	10.2	allosteric site	(Noort and Hulst, 2003; Tong et al., 2004; Ferguson et al., 2010)
(2)	Alcohol Dehydrogenase	1U3W	1.45	170	0.0	>12	n.a.	(Hansch et al., 1986; Dixit et al., 1981)
				240*	30.8	>12	protein surface	
(3)	Aldolase	1QO5	2.5	134*	0.0	>12	n.a.	(Dobryszycki et al., 1999a,b)
				239*	16.8	>12	protein surface	
				268	2.0	>12	n.a.	
				289*	7.6	10.8	protein surface	
(4)	Creatine Kinase	3B6R	2.0	74	2.6	>12	n.a.	(Matsuoka et al., 1996; Lin et al., 1994; Meng et al., 2001; Sheng et al., 2009)
				141	11.1	>12	protein surface	
				146	2.6	>12	n.a.	
				254	3.6	>12	protein surface	
				**283**	37.2	~9	active site	
(5)	Dopamine Receptor	3PBL	2.89	**114**	18.1	>12	binding site	(Erkekoglu and Baydar, 2014)
(6)	Dopamine Transporter (outward)	HM (6M2R)	2.8	**342**	3.7	>12	intracellular loops	(LoPachin and Barber, 2006)
				135	0.8	>12		
	Dopamine Transporter (inward)	HM (6DZZ)	3.6	**342**	12.8	>12		
				135	7.5	11.3		

#	Protein name	PDB code	Resolution (Å)	Cysteine	SASA ($Å^2$)	pK_a	Cys location	Reference
(7)	Enolase	2PSN	2.2	388<br>398*	7.7<br>36.0	>12<br>~9	n.a.<br>protein-protein interface	(Howland et al., 1980)
(8)	Estrogen Receptor	1ERE	3.1	381*<br>530*	37.5<br>68.5	>12<br>9.9	Cys-rich region	(Hogervorst et al., 2009; Aliau et al., 1999)
(9)	Glyceraldehyde-3-phosphate dehydrogenase	4WNC	1.99	**152**<br>156<br>247	32.8<br>0.2<br>0.0	6.6<br>>12<br>>12	active site<br>solvent exposed<br>PTM site	(Martyniuk et al., 2011)
(10)	Hemoglobin	6KA9	1.4	**93**<br>104	12.2<br>5.1	>12<br>>12	protein-protein interface	(Basile et al., 2008)
(11)	Immunoglobulin G1 H Nie	6ARP	1.7	395*	–[a]	–[a]	n.a.	(Feng and Lu, 2011a)
(12)	Immunoglobin kappa light chain	6N35	1.75	134*	–[a]	–[a]	n.a.	(Feng and Lu, 2011a)
(13)	Kinesin KIFC1	5WDH	2.25	663*	33.4	>12	protein surface	(Friedman et al., 2008)
(14)	Kinesin KIF2C	4UBF	3.0	260*<br>287*	9.4<br>56.4	>12<br>9.3	dimeric interface<br>dimeric interface	(Friedman et al., 2008)
(15)	NEM-sensitive factor	HM (3J94)	4.2	**264**	85.2	7.7	walker A motif	(LoPachin and Barber, 2006)
(16)	Sex Hormone-Binding Globulin	1KDM	2.35	164*<br>188*	–[a]	–[a]	protein surface<br>protein surface	(Hogervorst et al., 2009)
(17)	Topoisomerase IIa (ATPase domain)	1ZXM	1.87	170	0.0	>12	near active site	(Sciandrello et al., 2010)
(18)	Topoisomerase IIa (Toprim domain)	4FM9	2.90	997*	0.9	11.1	protein-DNA interface	(Sciandrello et al., 2010)
(19)	Vesicular proton ATPase	6WM2	3.1	**254**	11.0	10.3	walker A motif	(LoPachin and Barber, 2006)

[a]Cysteines involved in a disulfide bridge; hence, pK_a and solvent-accessible surface area (SASA) values could not be calculated.

Table S2: Protein microenvironment of the potential reactive cysteines. The analysis was performed using the same protein structures (listed here by their PDB codes) as in Table S1. Protein residues within a distance cutoff of 10Å from the respective Cys were analyzed. Asp/Glu and Arg/Lys residues are displayed with blue and red shades, respectively, whereas His residues are in green and other H-bonding capable residues in orange. In addition, for residues within 5Å from the candidate Cys, distances with respect the Cys sulfur atom are specified[a]. For candidate cysteines surrounded only by hydrophobic residues or surface water molecules, a gray shade (and the label n.a., not applicable) was used.

#	Protein name	PDB code	Cysteine	Environment
(1)	Albumin	6HSC	**34**	Lys41
				Asp38 (5.65Å)
				His39 (4.62Å)
				Thr79 (4.54Å), Tyr84 (2.98Å)
(2)	Alcohol Dehydrogenase	1U3W	170	Lys369
				Glu62 (5.98Å)
			240*	Lys233 (4.97Å)
(3)	Aldolase	1QO5	134*	Arg133 (6.83Å)
				Cys177 (4.07Å), Asn180 (5.32Å)
			239*	Lys241
				Asp195 (7.01Å), Asp197 (5.74Å)
				Tyr243 (4.43Å)
			268	n.a.
			289*	n.a.
(4)	Creatine Kinase	3B6R	74	n.a.
			141	Asp78 (5.08Å), Glu80 (7.77Å), Glu150 (4.11Å)
				His145 (4.58Å)
			146	Ser49 (2.91Å)
				Arg151 (4.14Å), Arg209 (5.28Å), Arg215
				Asn230 (6.27Å)
			254	Arg135
				Thr251 6.58(Å), Thr258 (6.46Å)
			**283**	Arg96, Arg132, Arg236, Arg341
				Glu232, Asp233 (8.73Å)
				Ser285 (3.26Å), Asn286 (5.32Å)

#	Protein name	PDB code	Cysteine	Environment
(5)	Dopamine Receptor	3PBL	**114**	Asp110 (7.57Å)
				Ser117 (6.52Å)
(6)	Dopamine Transporter (inward)	HM (6M2R)	**342**	Tyr115 (5.15Å), Gln122 (4.94Å), Thr339 (5.42Å)
			135	n.a.
	Dopamine Transporter (outward)	HM (6DZZ)	**342**	Tyr115 (5.48Å), Asn341 (6.64Å), Thr512 (5.45Å)
			135	n.a.
(7)	Enolase	2PSN	388	Lys357
				Glu141 (6.67Å)
				Asn139 (4.50Å), Gln360 (7.77Å)
			398*	Arg399 (4.39Å), Arg399’
				Lys192, Arg208 (7.18Å)
				Tyr188 (3.30Å)
(8)	Estrogen Receptor	1ERE	381*	Arg515’, Lys520’
				Glu380 (7.72Å), Glu385 (8.14Å)
				His377 (4.56Å), His513’, His516’, His547 (4.92Å)
				Thr460 (4.67Å)
			530*	Lys523 (4.42Å)
				Tyr526 (3.93Å)
(9)	Glyceraldehyde-3-phosphate dehydrogenase	4WNC	**152**	Arg234
				Glu317 (9.16Å)
				His179 (3.48Å)
				Thr153 (3.65Å), Tyr314 (4.37Å), Asn316 (5.0Å)
			156	Ser293 (3.57Å), Ser312 (3.83Å)
			247	n.a.
(10)	Hemoglobin	6KA9	**93**	Lys40’
				Asp94 (4.55Å),
				His146 (4.29Å) His97 (6.40Å)
			104	Arg31
				His103 (5.47Å)
				Ser35 (4.86Å), Gln127 (4.76Å)
(11)	Immunoglobulin G1 H Nie	6ARP	395*	Lys251

#	Protein name	PDB code	Cysteine	Environment
(12)	Immunoglobin kappa light chain	6N35	134*	Glu161
				Ser177 (6.29Å)
(13)	Kinesin KIFC1	5WDH	663*	Ser607 (4.18Å)
(14)	Kinesin KIF2C	4UBF	260*	Asp312 (4.29Å)
				His257 (5.18Å)
				Cys262 (5.61Å), Cys344, Cys560 (3.56Å)
			287*	Lys286 (6.77Å)
				Glu244 (5.44Å), Glu712 (5.11Å)
				Ser285 (Ser285Å), Cys287 (5.61Å)
(15)	NEM-sensitive factor	HM (3J94)	**264**	Arg271, Arg446
				Glu329, Glu446
(16)	Sex Hormone-Binding Globulin	1KDM	164*	Arg47 (4.85Å)
				Asp162 (7.69Å)
				His17 (4.56Å)
			188*	Arg47 (6.11Å)
				His17 (3.64Å)
(17)	Topoisomerase IIa (ATPase domain)	1ZXM	170	Ser174 (4.08Å)
(18)	Topoisomerase IIa (Toprim domain)	4FM9	997*	Arg863
				Tyr892, Asn905 (6.04Å), Tyr907 (5.24Å)
(19)	Vesicular proton ATPase	6M2R	**254**	Lys256, Arg400, Lys437 (6.37Å), Lys438
				Asp436 (6.97Å)

[a]For residues within 5Å from the candidate Cys, distances with respect the Cys sulfur atom were calculated using the following side chain atoms: carboxylate C for Asp/Glu, guanidinium C for Arg, amino N for Lys, closest imidazole N for His, hydroxyl O for Ser/Thr/Tyr, thiol S for Cys, indole N for Trp and closest amide N or O for Asn/Gln.

The ACR protein targets were further characterized based on their oligomerization state and protein class (see Table S3). The latter classification (see Figure S3) aims at connecting the corresponding protein target with the subcellular mechanism responsible for ACR toxicity, as discussed in the main text (see section 3.1). In addition, Table S3 includes the optimal pH range for optimal function of the corresponding protein, since the Michael addition reaction is favored with increasing pH (Lutolf et al., 2001; Nair et al., 2014). Most proteins in our dataset have an optimal pH close the physiological value of 7.

Table S3. Additional information for the ACR protein targets listed in Table S1, including oligomerization state and protein class, as well as the pH range for optimal protein function (as indicated in the corresponding reference).

#	Protein name	Oligomerization state	Protein class	Optimal pH	Reference
(1)	Albumin	monomer	plasma protein	7.4	(Baler et al., 2014)
(2)	Alcohol Dehydrogenase	dimer	enzyme	7-8	(Neto et al., 2011)
(3)	Aldolase	tetramer	enzyme	7.2	(Eagles and Iqbal, 1973)
(4)	Creatine Kinase	dimer	enzyme	6.5-7	(Szasz et al., 1976)
(5)	Dopamine Receptor	monomer	membrane receptor	∼7	(Kapolka et al., 2021)
(6)	Dopamine Transporter	monomer	membrane transporter	6.0-7.4	(Berfield et al., 1999)
(7)	Enolase	dimer	enzyme	6.8–6.9	(Shimizu et al., 1983)
(8)	Estrogen Receptor	dimer	nuclear receptor	6-8	(de Oliveira et al., 2022)
(9)	Glyceraldehyde-3-phosphate dehydrogenase	tetramer	enzyme	7.2-8.3	(Heinz and Freimüller, 1982)
(10)	Hemoglobin	tetramer	plasma protein	7.4	(Imai and Yonetani, 1975)
(11)	Immunoglobulin G1 H Nie	tetramer	plasma protein	∼6.5	(Riera and Álvarez, 2014)
(12)	Immunoglobin kappa light chain	tetramer	plasma protein	∼6.5	(Riera and Álvarez, 2014)
(13)	Kinesin KIFC1	monomer	ATPase*	6.8-7.2	(Verhey et al., 1998)
(14)	Kinesin KIF2C	dimer	ATPase*	6.8-7.2	(Verhey et al., 1998)
(15)	NEM-sensitive factor	hexamer	ATPase	9.0	(Whiteheart et al., 1994)
(16)	Sex Hormone-Binding Globulin	dimer	plasma protein	>5	(Selby, 1990)
(17)	Topoisomerase IIa (ATPase domain)	dimer	enzyme	7.5	(Gardiner et al., 1998)
(18)	Topoisomerase IIa (Toprim domain)	dimer	enzyme	7.5	(Osheroff et al., 1983)
(19)	Vesicular proton ATPase	monomer	ATPase*	7	(Osheroff et al., 1981)

*For both kinesins and the vesicular proton ATPase, their ATPase domain is the one considered in the covalent docking calculations and thus was used for the protein class and oligomeric state classification.

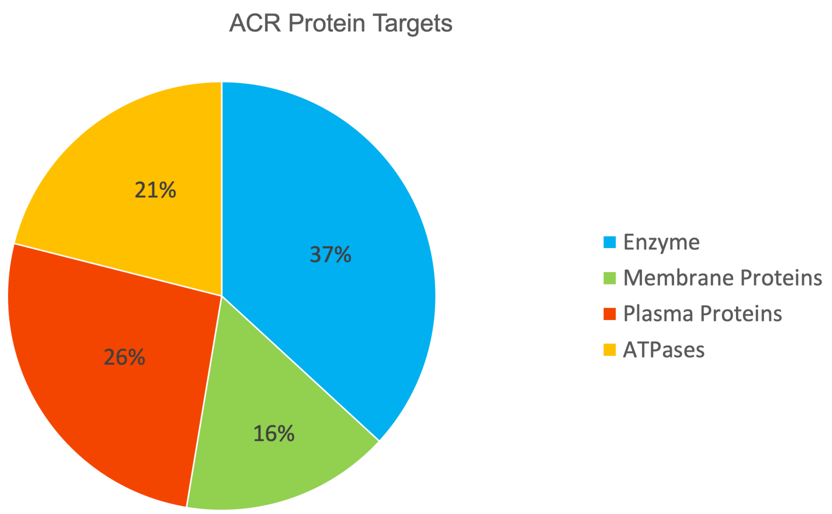

Figure S3: Functional classification of the acrylamide protein targets in the dataset compiled for this study.

1.2 Validation of the covalent docking approach

Covalent docking to the reactive cysteine of each target protein (see Table S1) was performed using Haddock (version 2.2.) (De Vries et al., 2010; van Zundert et al., 2016). We followed the standard covalent docking protocol of Haddock (HADDOCK developer team, 2018), which was initially developed for covalent inhibitors of cathepsin K (HADDOCK developer team, 2018). Here we have validated it for Cys-ACR adducts using the available experimental structures for such covalent ligand-protein complexes. In particular, we searched in the Protein Data Bank (Berman et al., 2000; Burley et al., 2021) for experimental structures containing the ligand name ROP (i.e. propionamide, the product of the Michael addition reaction, as explained in the main text, section 2.3.1); see (RCSB PDB Ligand Summary ROP, 2022). We then filtered for entries in which this ligand is covalently linked to the protein, rendering a total of five X-ray structures (PDB codes 3ZVI (Raj et al., 2012), 4GYL (Weber et al., 2013), 4IZV, 4IZU, and 4WGF (Groftehauge et al., 2015)), of which one (PDB 4IZU) contains two covalent ACR adducts (with C53 and C145). All these structures correspond to bacterial enzymes and have resolution between 1.4Å and 2.3Å. For the redocking calculations, the protein structures were stripped from the ROP ligand and submitted to the same covalent docking protocol described in the main text (see section 2.3.2). The redocking and crystallographic poses are compared in Table S4, in terms of their protein-ligand interactions. Most of the protein-ligand interactions observed in the crystal structures are reproduced in the redocking poses. Additional interactions are present in the redocking poses, which we ascribed to the increased flexibility of acrylamide in the redocking calculations (which include a final refinement molecular dynamics step at 300 K; see step 3b in section 2.3.2 in the main text) compared to the X-ray structures (solved at 100K-110K). For further information on the redocking calculations, we refer the reader to Supplementary Material 2, which includes the Haddock score and size of the redocking clusters, as well as the Haddock score of the top four poses of each cluster.

Table S4. Comparison of the protein-ligand interaction fingerprints of the crystal structures containing Cys-ACR adducts and the corresponding redocking poses. For each experimental structure, the PDB code, reactive Cys and resolution (between parentheses) is given. The presence/absence of an interaction is indicated with a black/white box, respectively; H-bonds were defined as explained in the main text (see section 2.4).

PDB code 3ZVI - C361 (1.90 Å)

Residue	Interaction	X-ray	Redocking
Gln73	HB acceptor	■	■
Thr360	HB donor	■	■

PDB code 4GYL - C166 (1.90 Å)

Residue	Interaction	X-ray	Redocking
Tyr60	HB acceptor	■	■
Lys134	HB donor	■	■
Asp167	HB acceptor	□	■
Tyr192	HB acceptor	□	■

PDB code 4IZV - C53 (1.65 Å)

Residue	Interaction	X-ray	Redocking
Pro89	HB acceptor	■	□

PDB code 4IZU - C53 (1.40 Å)

Residue	Interaction	X-ray	Redocking
Pro49	HB acceptor	□	■
Ser50	HB acceptor	□	■
Pro89	HB acceptor	■	□

PDB code 4IZU - C145 (1.40 Å)

Residue	Interaction	X-ray	Redocking
Gln41	HB donor	■	■
Gln41	HB acceptor	□	■
Thr47	HB acceptor	□	■
Thr47	HB donor	■	■
Lys111	HB donor	■	■
Glu119	HB acceptor	■	■

PDB code 4WGF - C118 (2.34 Å)

Residue	Interaction	X-ray	Redocking
Asp19	HB acceptor	■	■
Ser59	HB acceptor	■	□
Ser59	HB donor	□	■
Asn65	HB donor	■	■
Trp176	HB donor	■	□

1.3 Proteins with experimentally verified reactive cysteine

Out of the 19 proteins in our dataset, the specific Cys targeted by acrylamide is known for eight. Below we present the results of the covalent docking performed for each of these proteins. In some cases (creatine kinase, glyceraldehyde-3-phosphate dehydrogenase and hemoglobin) experimental evidence suggests more than one cysteine targeted by acrylamide, but with different reactivity; thus we performed a docking calculation for each of the cysteines within the same protein target separately.

The outcome of these dockings is presented in part in the main text and in part here below. Namely, the main text includes an overview of the results and their discussion, as well as Table 1, which reports the Haddock score and cluster size of the top (best scored) cluster. Here below we show the protein-ligand interaction analysis of the docking poses of the top cluster; additional clusters are also considered if their Haddock scores fall within standard deviation of the top cluster. Such analysis was carried out with ProLIF (Bouysset and Fiorucci, 2021), as explained in section 2.4 of the main text; if no interactions were detected, no scheme is shown. For further details, we refer the reader to Supplementary Material 2, which includes the full report of the docking results.

1.3.1 Human Serum Albumin (HSA)

Covalent docking for C34 of albumin resulted in two main clusters (numbers 2 and 1) with Haddock scores within standard deviation of each other (see Supplementary Material 2).

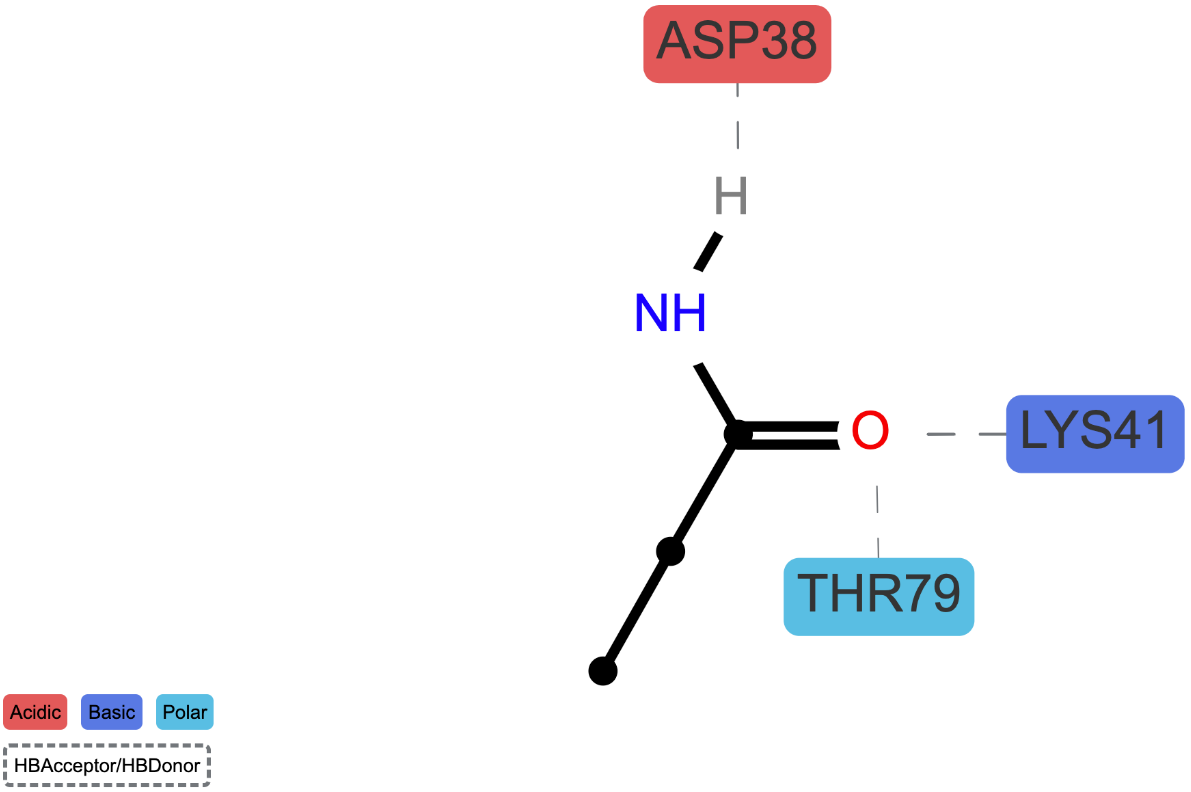

Albumin (C34) - Cluster 2			
residue	number	interaction	occurrence
Asp	38	HB acceptor	0.14
Lys	41	HB donor	0.19
Thr	79	HB donor	0.10

Figure S4: Modeled covalent adduct between acrylamide (ACR) and the target protein. (*Top*) Schematic representation of acrylamide and the hydrogen bonds (HBs) formed with the surrounding binding site residues. Residues are colored according to their physicochemical properties, as shown in the figure legend, and the corresponding HB frequency is represented through the width of the dashed line. (*Bottom*) Table indicating the target protein, reactive Cys and docking cluster considered, together with the list of detected protein-ligand HBs and their respective frequency.

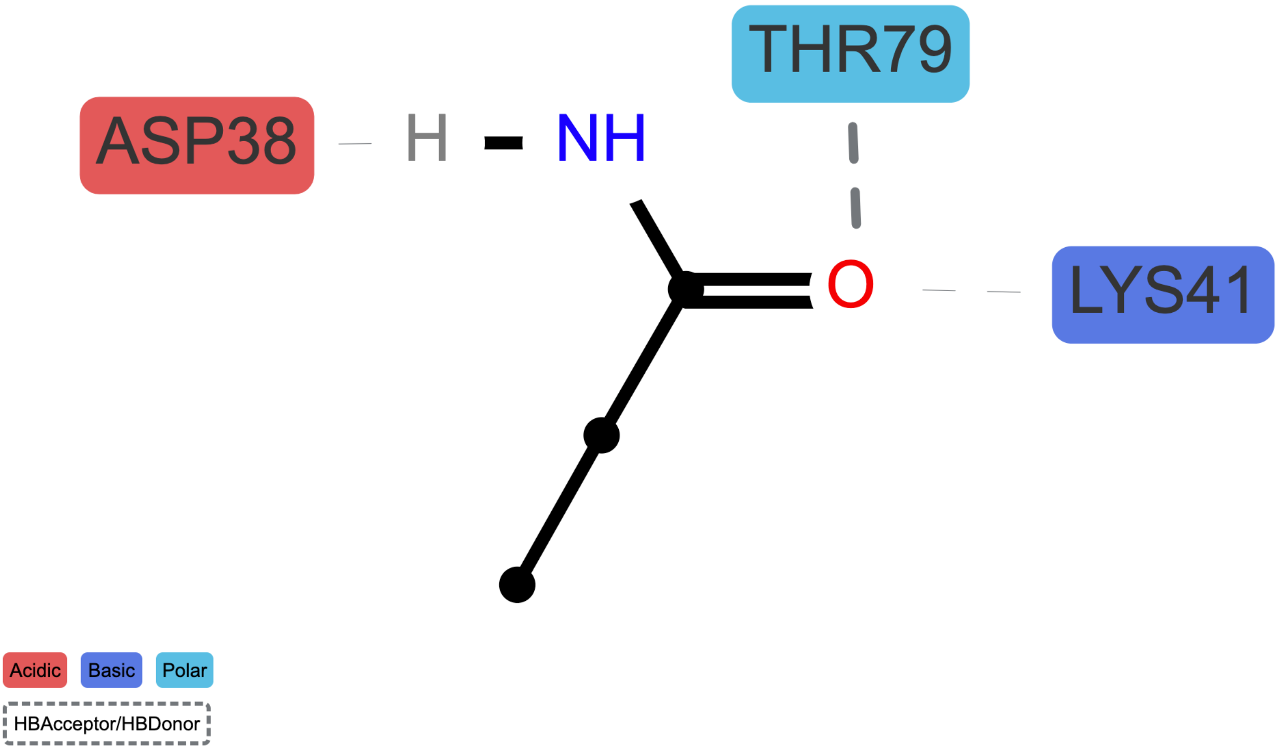

Albumin (C34) - Cluster 1			
residue	number	interaction	occurrence
Asp	38	HB acceptor	0.04
Lys	41	HB donor	0.04
Thr	79	HB donor	0.48

Figure S5: Modeled covalent adduct between acrylamide (ACR) and the target protein. (*Top*) Schematic representation of acrylamide and the hydrogen bonds (HBs) formed with the surrounding binding site residues. Residues are colored according to their physicochemical properties, as shown in the figure legend, and the corresponding HB frequency is represented through the width of the dashed line. (*Bottom*) Table indicating the target protein, reactive Cys and docking cluster considered, together with the list of detected protein-ligand HBs and their respective frequency.

1.3.2 Creatine Kinase (CK)

Because of the the biphasic time dependent inactivation of CK by ACR observed in enzymatic assays (Sheng et al., 2009), we performed covalent docking for several cysteine residues (see Table S1). Covalent docking for the experimentally known primary site of ACR modification in CK, C283, resulted in one main cluster (number 1); the top pose is shown Figure 4B in the main text and the corresponding protein-ligand interaction fingerprints in Figure S11 below. One main cluster (number 1) was also obtained for the secondary ACR binding site (C141) predicted in this study (see Figures S6 and S7). The docking results for the alternative cysteine discussed in the main text (C146, see section 3.2.2) are shown in Figures S6 and S8-S10. For both C141 and C146, some docking poses showed a distance between ACR and the corresponding Cys too long to be compatible with a covalent bond. Such non-covalently bound ligand poses were excluded when calculating the average Haddock score and cluster size shown in Table 1 in the main text and the protein-ligand interaction fingerprints in Figures S7 and S8-S10.

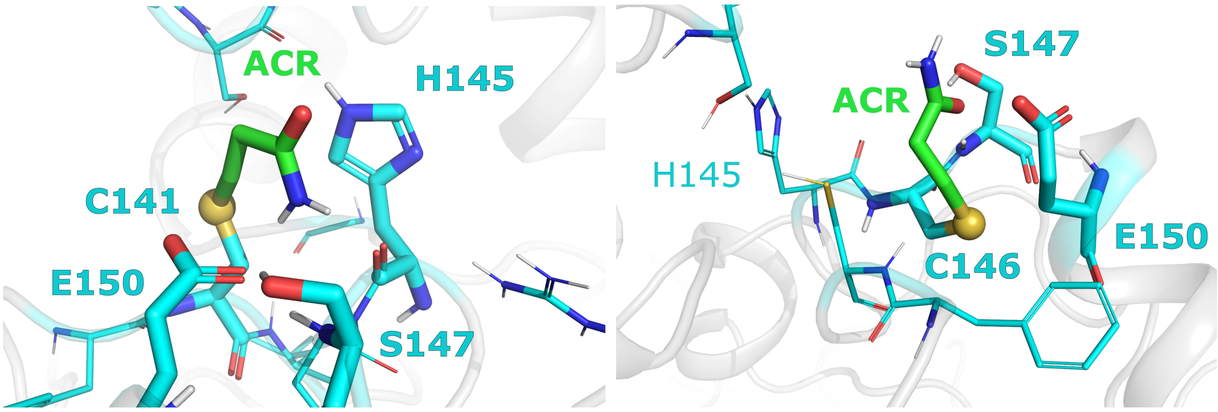

Figure S6: Representative covalent binding poses of ACR and CK C141 (left) and C146 (right). Acrylamide and its surrounding residues are represented as sticks, with carbon atoms colored in green and cyan, respectively. The sulfur atom between the reactive cysteine residue and the adduct is shown as a sphere. Residues forming hydrogen bonds (HBs) with ACR are displayed with thicker sticks and labeled. HBs present in more than 60% of the docking poses are shown with a dashed line. Residues within 5 Åof the adduct that can potentially favor the Michael addition reaction are shown with thinner lines.

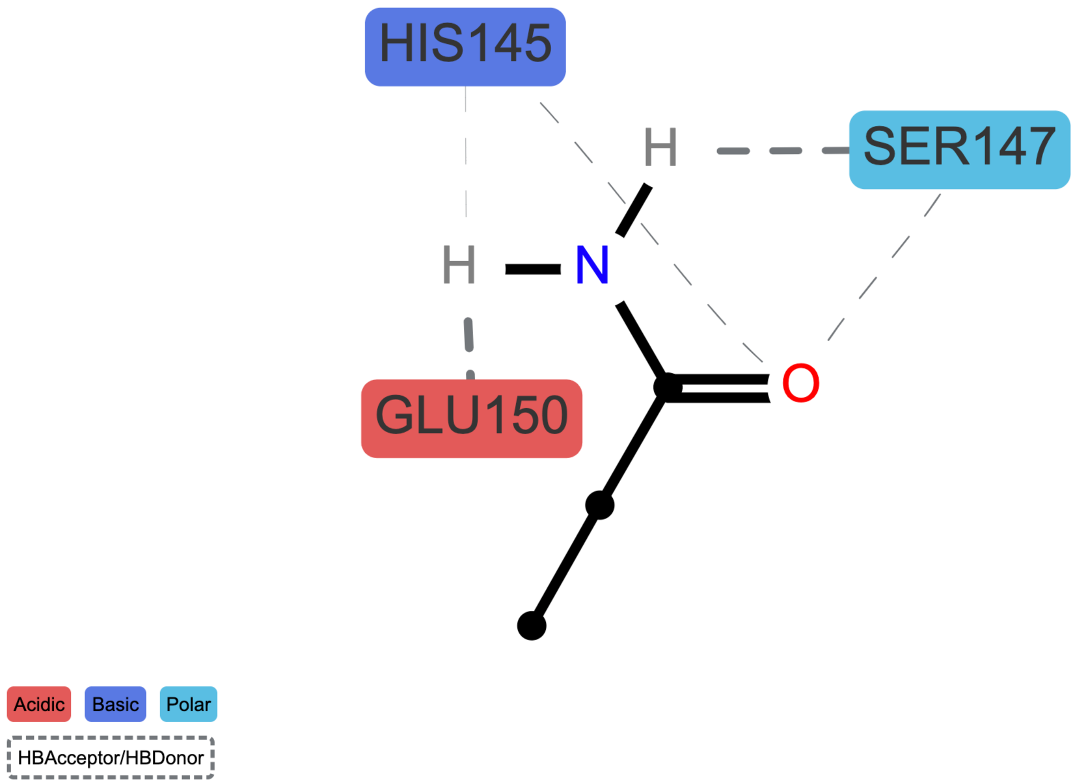

Creatine Kinase (C141) - Cluster 1			
residue	number	interaction	occurrence
His	145	HB donor	0.10
His	145	HB acceptor	0.03
Ser	147	HB donor	0.11
Ser	147	HB acceptor	0.51
Glu	150	HB acceptor	0.57

Figure S7: Modeled covalent adduct between acrylamide (ACR) and the target protein. (*Top*) Schematic representation of acrylamide and the hydrogen bonds (HBs) formed with the surrounding binding site residues. Residues are colored according to their physicochemical properties, as shown in the figure legend, and the corresponding HB frequency is represented through the width of the dashed line. (*Bottom*) Table indicating the target protein, reactive Cys and docking cluster considered, together with the list of detected protein-ligand HBs and their respective frequency.

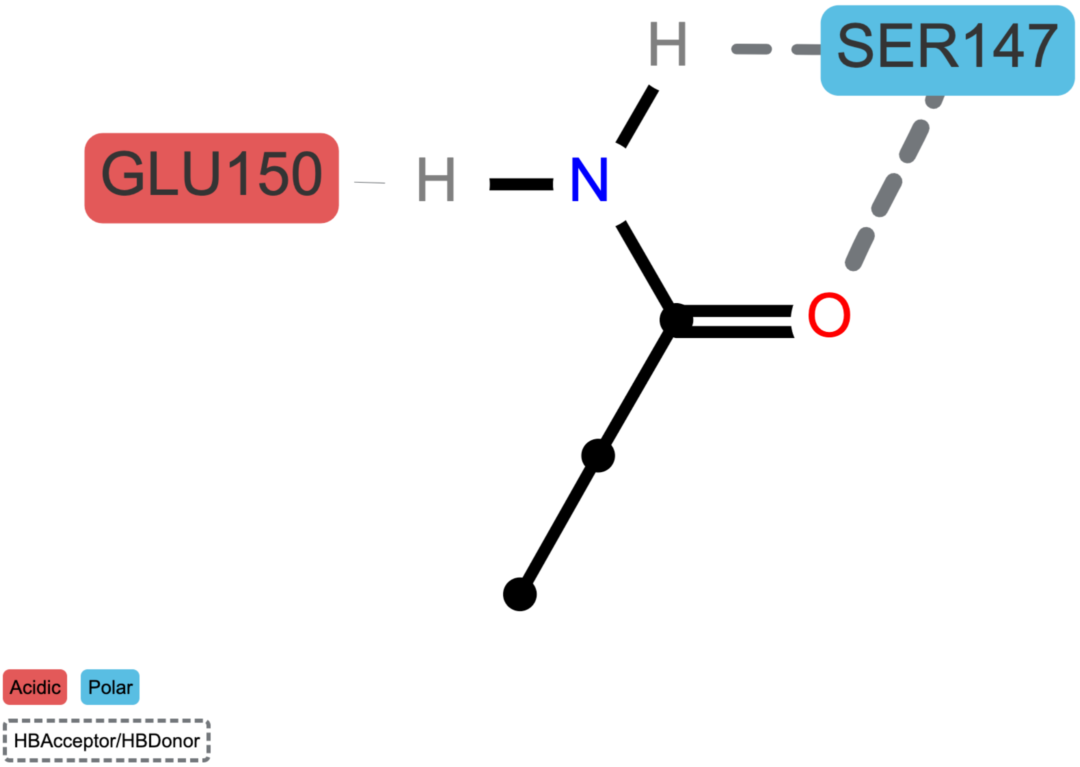

Creatine Kinase (C146) - Cluster 1			
residue	number	interaction	occurrence
Ser	147	HB donor	0.95
Ser	147	HB acceptor	0.59
Glu	150	HB acceptor	0.05

Figure S8: Modeled covalent adduct between acrylamide (ACR) and the target protein. (*Top*) Schematic representation of acrylamide and the hydrogen bonds (HBs) formed with the surrounding binding site residues. Residues are colored according to their physicochemical properties, as shown in the figure legend, and the corresponding HB frequency is represented through the width of the dashed line. (*Bottom*) Table indicating the target protein, reactive Cys and docking cluster considered, together with the list of detected protein-ligand HBs and their respective frequency.

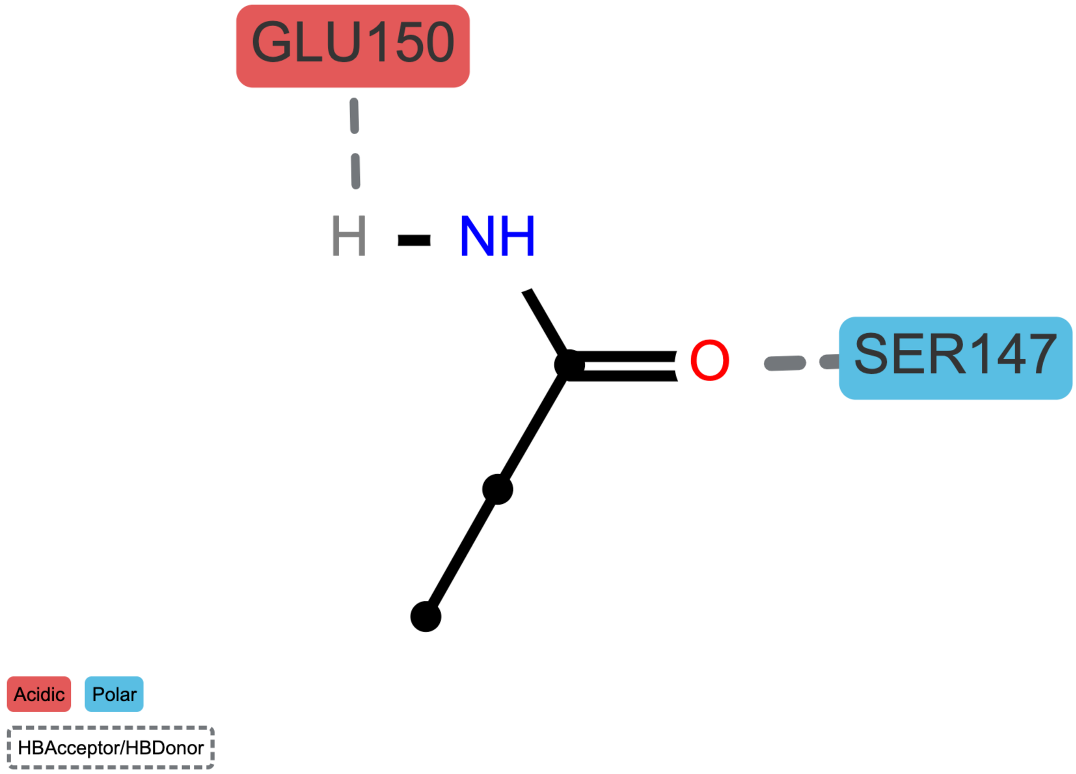

Creatine Kinase (C146) - Cluster 2			
residue	number	interaction	occurrence
Ser	147	HB donor	0.88
Glu	150	HB acceptor	0.53

Figure S9: Modeled covalent adduct between acrylamide (ACR) and the target protein. (*Top*) Schematic representation of acrylamide and the hydrogen bonds (HBs) formed with the surrounding binding site residues. Residues are colored according to their physicochemical properties, as shown in the figure legend, and the corresponding HB frequency is represented through the width of the dashed line. (*Bottom*) Table indicating the target protein, reactive Cys and docking cluster considered, together with the list of detected protein-ligand HBs and their respective frequency.

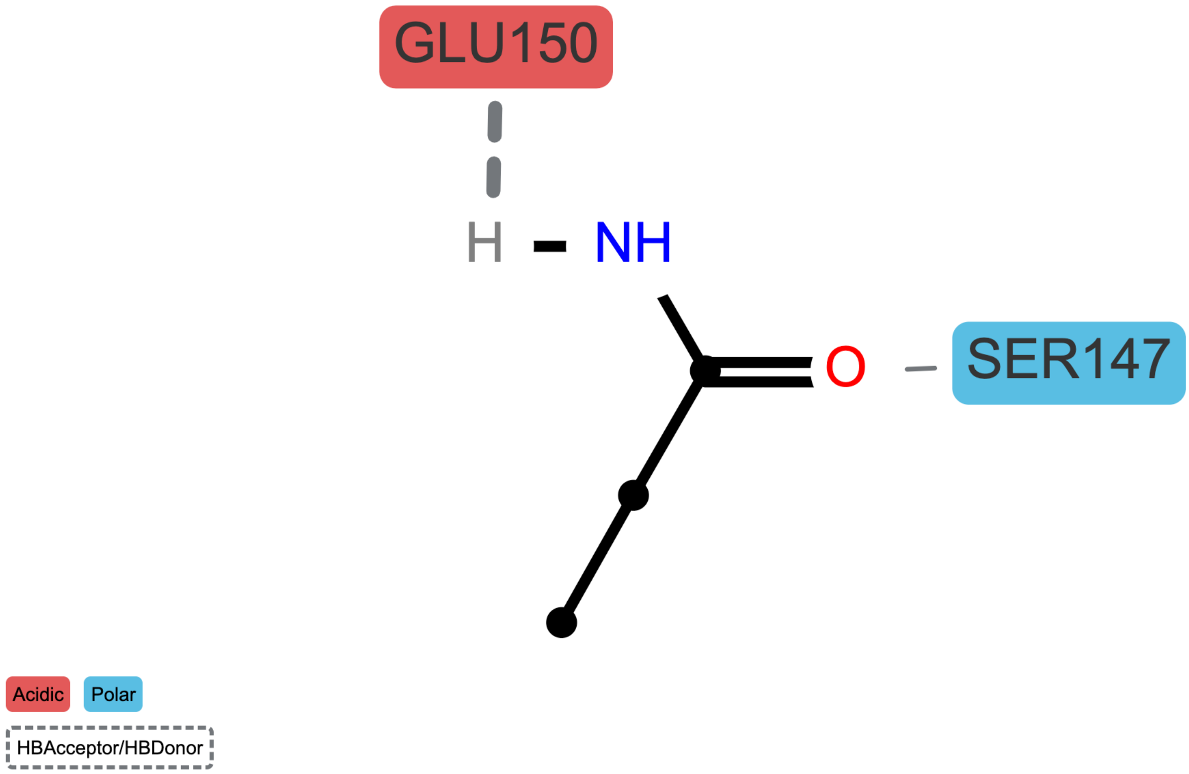

Creatine Kinase (C146) - Cluster 5			
residue	number	interaction	occurrence
Ser	147	HB donor	0.29
Glu	150	HB acceptor	0.86

Figure S10: Modeled covalent adduct between acrylamide (ACR) and the target protein. (*Top*) Schematic representation of acrylamide and the hydrogen bonds (HBs) formed with the surrounding binding site residues. Residues are colored according to their physicochemical properties, as shown in the figure legend, and the corresponding HB frequency is represented through the width of the dashed line. (*Bottom*) Table indicating the target protein, reactive Cys and docking cluster considered, together with the list of detected protein-ligand HBs and their respective frequency.

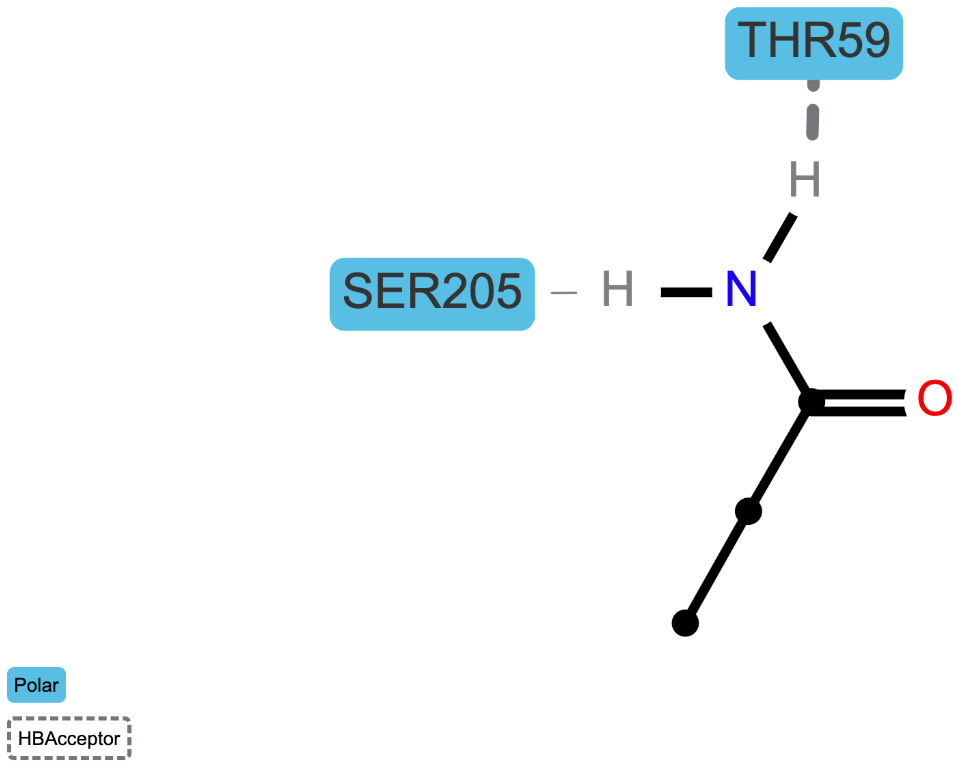

Creatine Kinase (C283) - Cluster 1			
residue	number	interaction	occurrence
Thr	59	HB acceptor	0.89
Ser	205	HB acceptor	0.20

Figure S11: Modeled covalent adduct between acrylamide (ACR) and the target protein. (*Top*) Schematic representation of acrylamide and the hydrogen bonds (HBs) formed with the surrounding binding site residues. Residues are colored according to their physicochemical properties, as shown in the figure legend, and the corresponding HB frequency is represented through the width of the dashed line. (*Bottom*) Table indicating the target protein, reactive Cys and docking cluster considered, together with the list of detected protein-ligand HBs and their respective frequency.

In addition, we performed a multibody docking to investigate the possibility of simultaneous binding of two ACR molecules to C74 and C283, as proposed in (Sheng et al., 2009). A representative docking pose is displayed in Figure S12, showing that binding of the first ACR molecule to C283 blocks access to C74. Therefore, in our hands, C74 cannot be modified by ACR, unlike the model proposed in reference (Sheng et al., 2009).

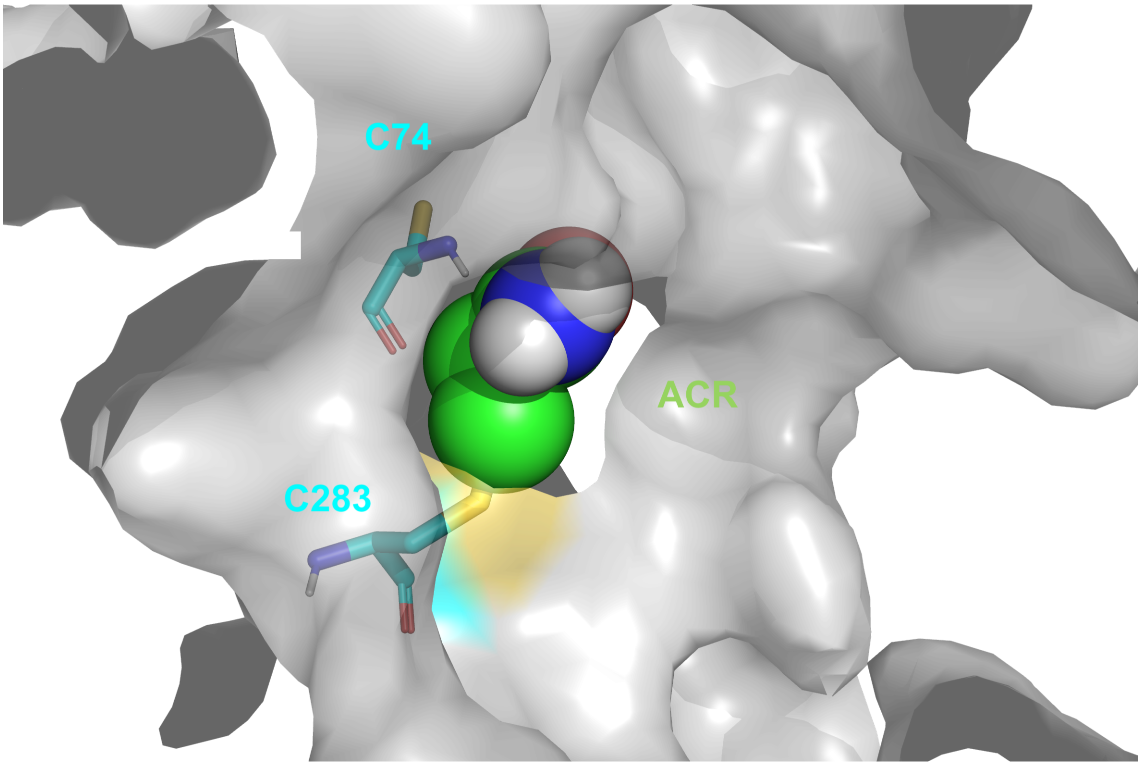

Figure S12: Representative pose of the multibody docking for creatine kinase. ACR is shown as spheres, C283 and C74 in stick representation and the surface of CK is shown in grey. A molecule of ACR is covalently bound to C283; as a result, C74 is no longer accessible to the second ACR molecule coming from the solution. Indeed, the covalent docking calculation placed the second molecule of ACR (not shown) outside the cavity where C74 and C283 are located.

1.3.3 Dopamine D3 Receptor (D3R)

Covalent docking for C114 of D3R resulted in one main top cluster (number 1).

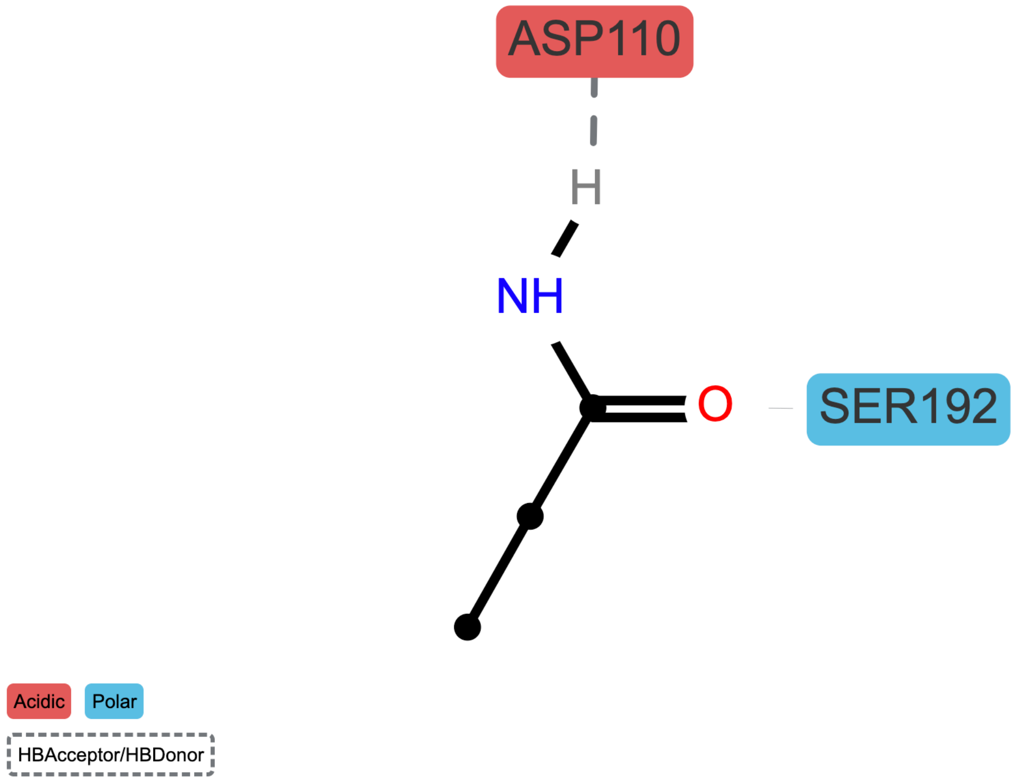

Dopamine D3 Receptor (C114) - Cluster 1			
residue	number	interaction	occurrence
Asp	110	HB acceptor	0.82
Ser	192	HB donor	0.01

Figure S13: Modeled covalent adduct between acrylamide (ACR) and the target protein. (*Top*) Schematic representation of acrylamide and the hydrogen bonds (HBs) formed with the surrounding binding site residues. Residues are colored according to their physicochemical properties, as shown in the figure legend, and the corresponding HB frequency is represented through the width of the dashed line. (*Bottom*) Table indicating the target protein, reactive Cys and docking cluster considered, together with the list of detected protein-ligand HBs and their respective frequency.

1.3.4 Dopamine Transporter (DAT)

Covalent docking for C342 of DAT (i.e. the primary site of ACR modification for the wild-type transporter) was performed for both the inward and outward conformations (IF and OF, respectively). One main top cluster (number 1) was obtained for the IF state, but two (clusters 1 and 4) for the OF state.

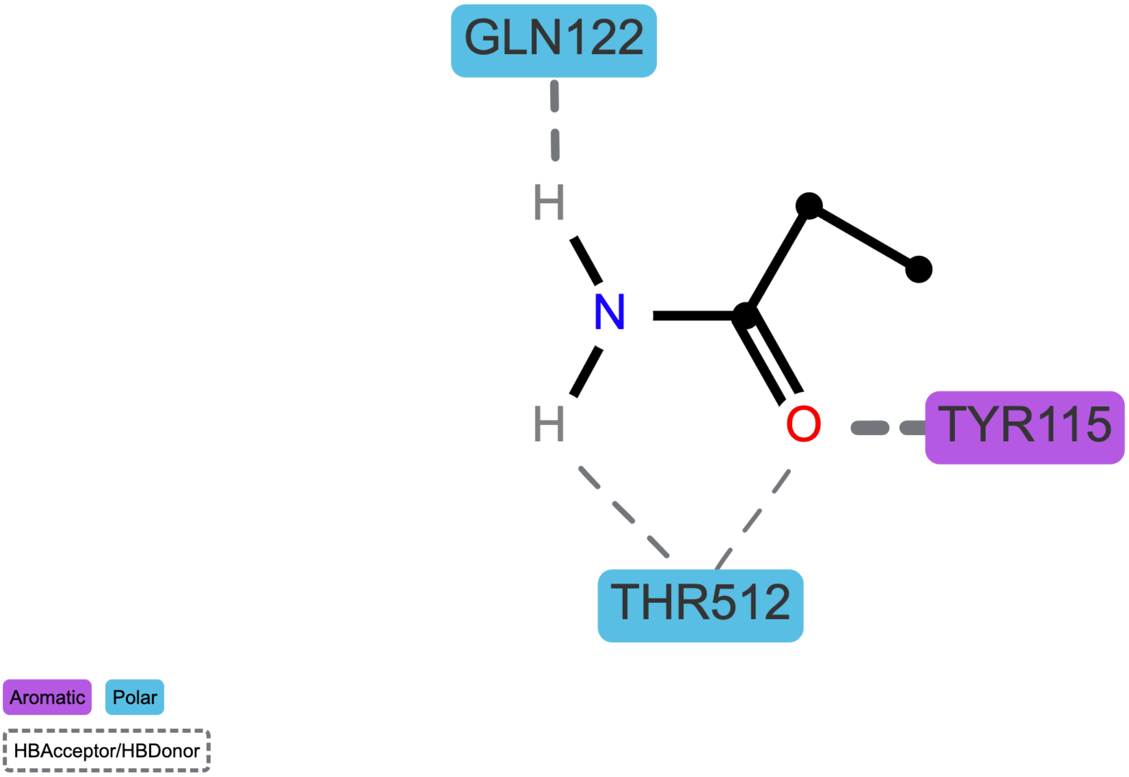

Dopamine Transporter (IF) (C342) - Cluster 1			
residue	number	interaction	occurrence
Tyr	115	HB donor	1.00
Gln	122	HB acceptor	0.59
Thr	512	HB donor	0.31
Thr	512	HB acceptor	0.38

Figure S14: Modeled covalent adduct between acrylamide (ACR) and the target protein. (*Top*) Schematic representation of acrylamide and the hydrogen bonds (HBs) formed with the surrounding binding site residues. Residues are colored according to their physicochemical properties, as shown in the figure legend, and the corresponding HB frequency is represented through the width of the dashed line. (*Bottom*) Table indicating the target protein, reactive Cys and docking cluster considered, together with the list of detected protein-ligand HBs and their respective frequency.

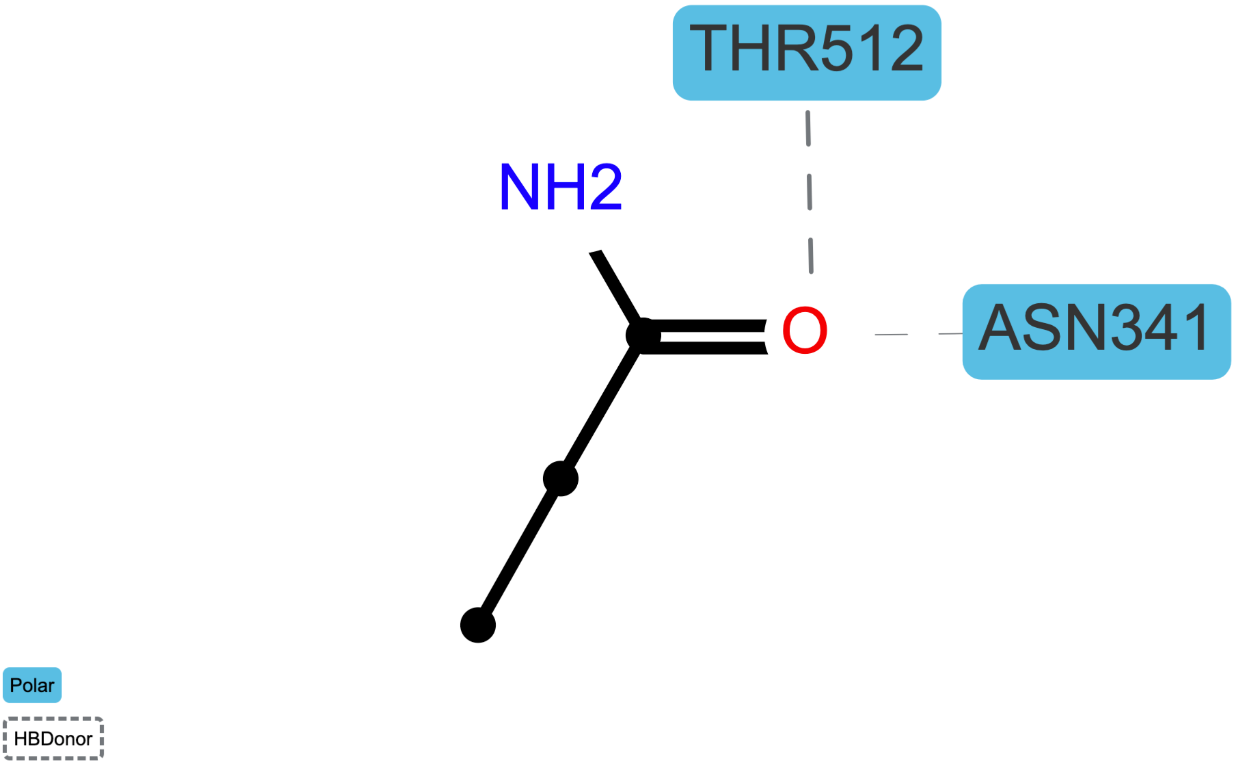

Dopamine Transporter (OF) (C342) - Cluster 1			
residue	number	interaction	occurrence
Asn	341	HB donor	0.06
Thr	512	HB donor	0.24

Figure S15: Modeled covalent adduct between acrylamide (ACR) and the target protein. (*Top*) Schematic representation of acrylamide and the hydrogen bonds (HBs) formed with the surrounding binding site residues. Residues are colored according to their physicochemical properties, as shown in the figure legend, and the corresponding HB frequency is represented through the width of the dashed line. (*Bottom*) Table indicating the target protein, reactive Cys and docking cluster considered, together with the list of detected protein-ligand HBs and their respective frequency.

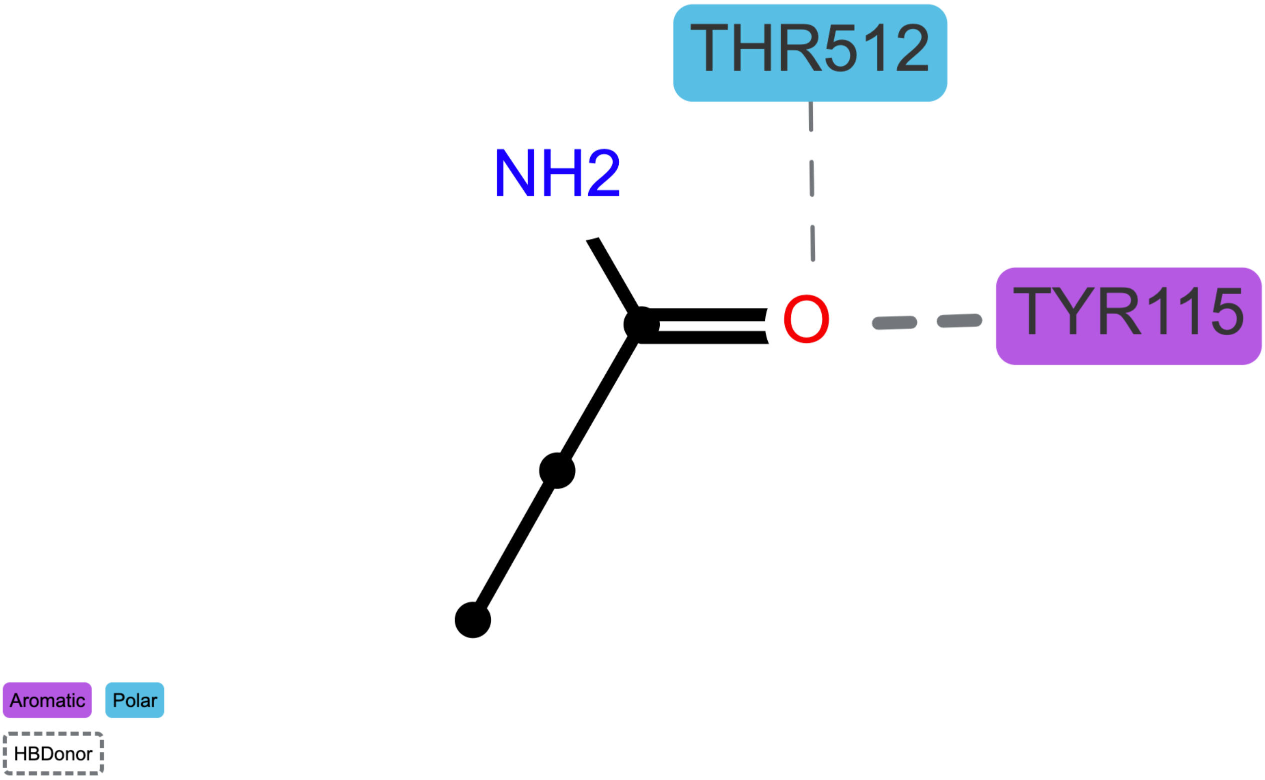

Dopamine Transporter (outward) (C342) - Cluster 4			
residue	number	interaction	occurrence
Thr	115	HB donor	0.67
Thr	512	HB donor	0.22

Figure S16: Modeled covalent adduct between acrylamide (ACR) and the target protein. (*Top*) Schematic representation of acrylamide and the hydrogen bonds (HBs) formed with the surrounding binding site residues. Residues are colored according to their physicochemical properties, as shown in the figure legend, and the corresponding HB frequency is represented through the width of the dashed line. (*Bottom*) Table indicating the target protein, reactive Cys and docking cluster considered, together with the list of detected protein-ligand HBs and their respective frequency.

1.3.5 Glyceraldehyde-3-phosphate dehydrogenase (GAPDH)

For GAPDH, covalent docking was performed for three cysteines: C152, C156 and C247, which can be modified at increasing ACR concentrations (Martyniuk et al., 2011). Docking with C152 resulted in one top cluster (number 1); the top pose is shown in the main text (Figure 4F) and the corresponding protein-ligand fingerprint in Figure S18. For C156 and C247, the generated docking poses were more diverse, resulting in either two clusters (numbers 1 and 2) or five clusters (numbers 1-5), respectively, with Haddock scores within standard deviation of the top (best scored) cluster. The top docking poses are shown in Figure S17, and the protein-ligand fingerprints of the corresponding dockings are presented in Figure S19 for C156 and Figure S20 for C247. As explained for CK (see section 1.3.2), docking poses with a distance between ACR and the corresponding Cys too long to be compatible with a C-S covalent bond were excluded from the analysis.

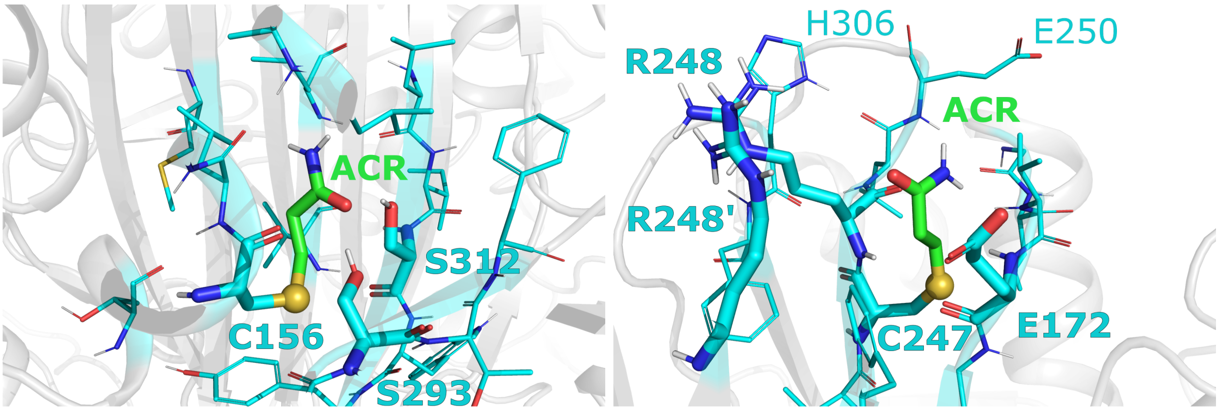

Figure S17: Representative covalent binding poses of ACR and GAPDH C156 (left) and C247 (right). Acrylamide and its surrounding residues are represented as sticks, with carbon atoms colored in green and cyan, respectively. The sulfur atom between the reactive cysteine residue and the adduct is shown as a sphere. Residues forming hydrogen bonds (HBs) with ACR are displayed with thicker sticks and labeled. HBs present in more than 60% of the docking poses are shown with a dashed line. Residues within 5 Åof the adduct that can potentially favor the Michael addition reaction are shown with thinner lines.

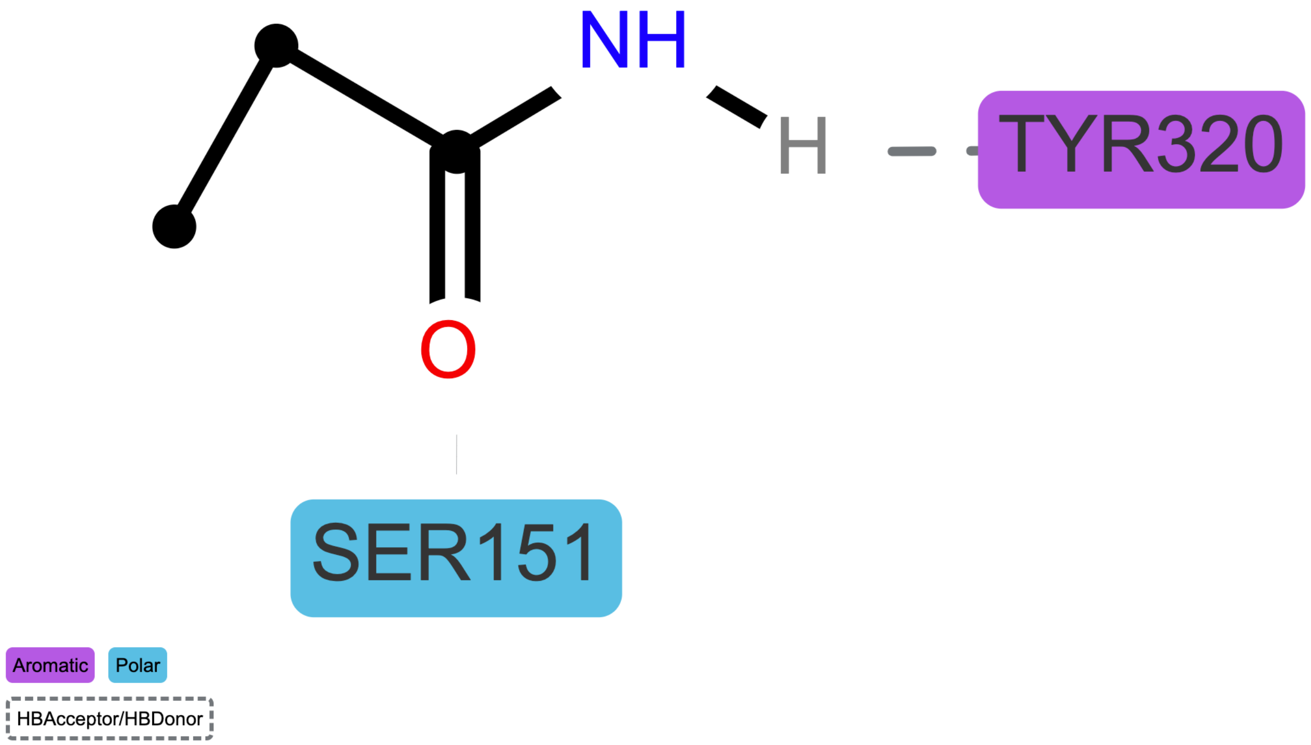

Glyceraldehyde-3-Phosphate Dehydrogenase (C152) - Cluster 1			
residue	number	interaction	occurrence
Ser	151	HB donor	0.01
Tyr	320	HB acceptor	0.48

Figure S18: Modeled covalent adduct between acrylamide (ACR) and the target protein. (*Top*) Schematic representation of acrylamide and the hydrogen bonds (HBs) formed with the surrounding binding site residues. Residues are colored according to their physicochemical properties, as shown in the figure legend, and the corresponding HB frequency is represented through the width of the dashed line. (*Bottom*) Table indicating the target protein, reactive Cys and docking cluster considered, together with the list of detected protein-ligand HBs and their respective frequency.

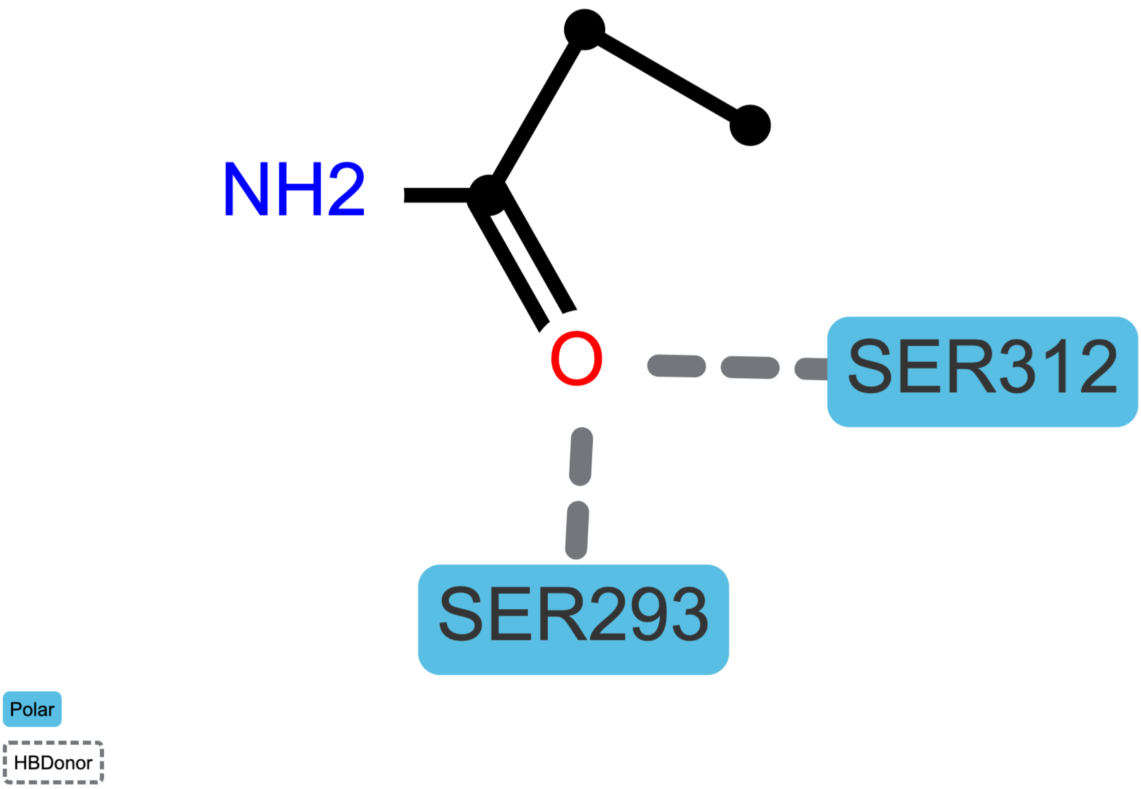

Glyceraldehyde-3-Phosphate Dehydrogenase (C156) - Cluster 1			
residue	number	interaction	occurrence
Ser	293	HB donor	1.00
Ser	312	HB donor	1.00

Figure S19: Modeled covalent adduct between acrylamide (ACR) and the target protein. (*Top*) Schematic representation of acrylamide and the hydrogen bonds (HBs) formed with the surrounding binding site residues. Residues are colored according to their physicochemical properties, as shown in the figure legend, and the corresponding HB frequency is represented through the width of the dashed line. (*Bottom*) Table indicating the target protein, reactive Cys and docking cluster considered, together with the list of detected protein-ligand HBs and their respective frequency.

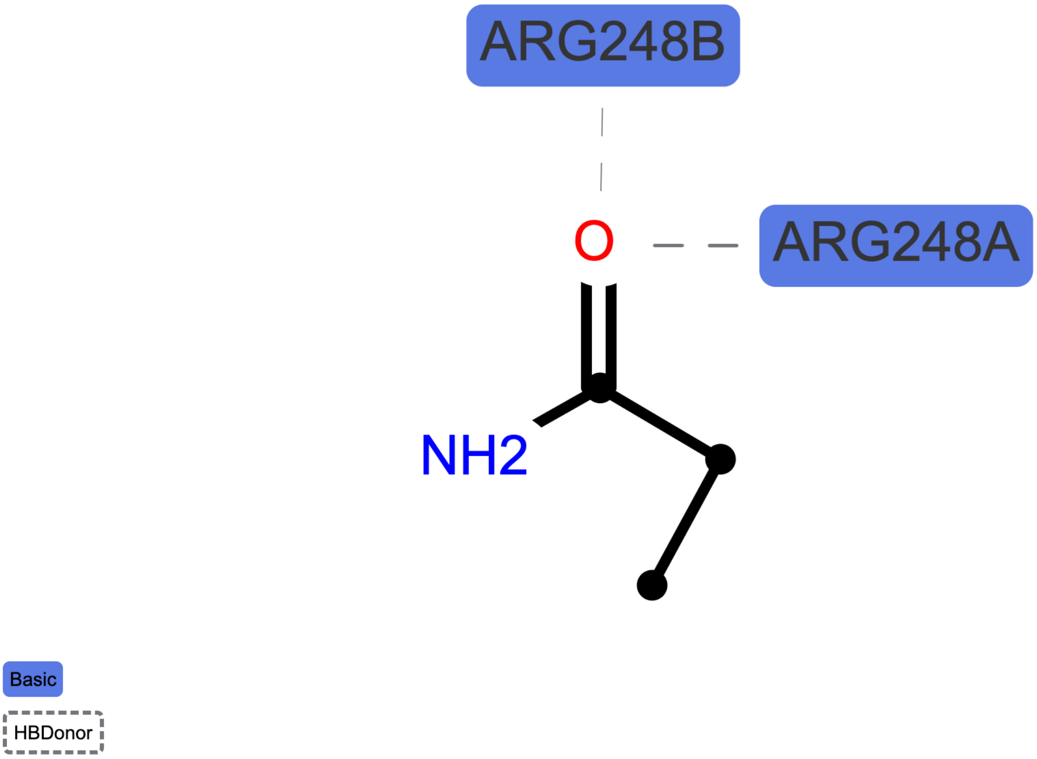

Glyceraldehyde-3-Phosphate Dehydrogenase (C247) - Cluster 1			
residue	number	interaction	occurrence
Arg	248 (chain A)	HB donor	0.21
Arg	248 (chain B)	HB acceptor	0.07

Figure S20: Modeled covalent adduct between acrylamide (ACR) and the target protein. (*Top*) Schematic representation of acrylamide and the hydrogen bonds (HBs) formed with the surrounding binding site residues. Residues are colored according to their physicochemical properties, as shown in the figure legend, and the corresponding HB frequency is represented through the width of the dashed line. (*Bottom*) Table indicating the target protein, reactive Cys and docking cluster considered, together with the list of detected protein-ligand HBs and their respective frequency.

1.3.6 Hemoglobin (Hb)

Covalent docking for Hb was performed for two cysteines. For C104 in the α subunit, the resulting poses did not exhibit a properly formed C-S bond, suggesting that adduct formation is less favorable for this cysteine. Instead, for Hb C93 (β subunit), five main clusters (numbers 1-5) were obtained with Haddock score within standard deviation of top (best scored) cluster; their protein-ligand interaction profiles are reported below.

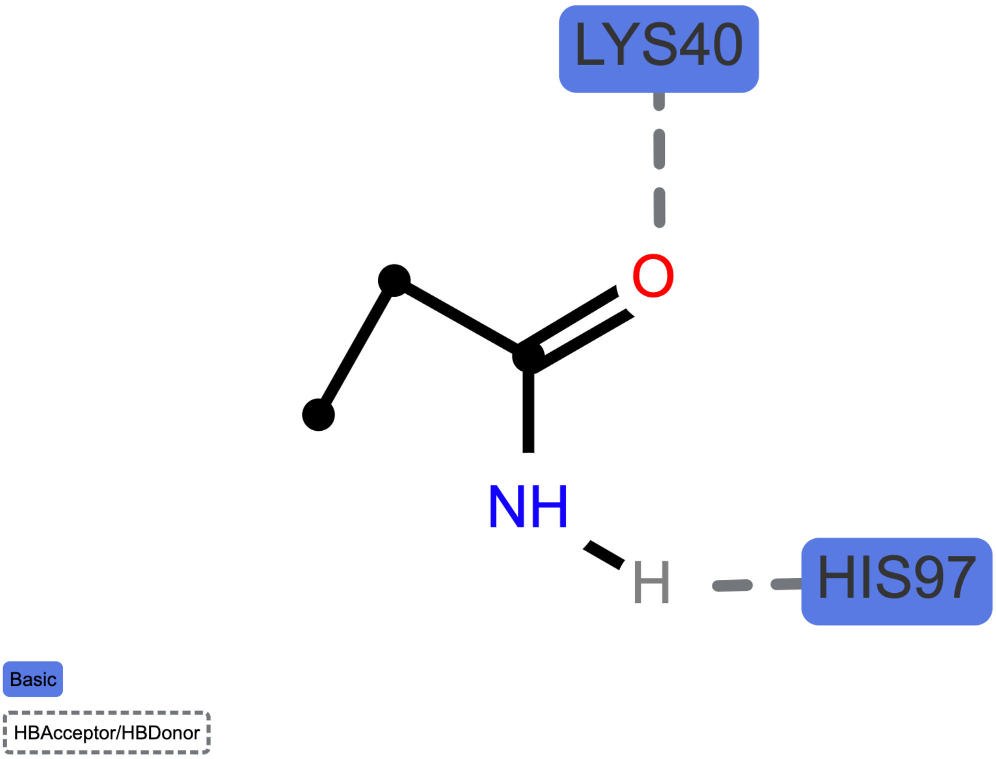

Hemoglobin (C93) - Cluster 1			
residue	number	interaction	occurrence
His	97	HB acceptor	0.77
Lys	40	HB donor	0.66

Figure S21: Modeled covalent adduct between acrylamide (ACR) and the target protein. (*Top*) Schematic representation of acrylamide and the hydrogen bonds (HBs) formed with the surrounding binding site residues. Residues are colored according to their physicochemical properties, as shown in the figure legend, and the corresponding HB frequency is represented through the width of the dashed line. (*Bottom*) Table indicating the target protein, reactive Cys and docking cluster considered, together with the list of detected protein-ligand HBs and their respective frequency.

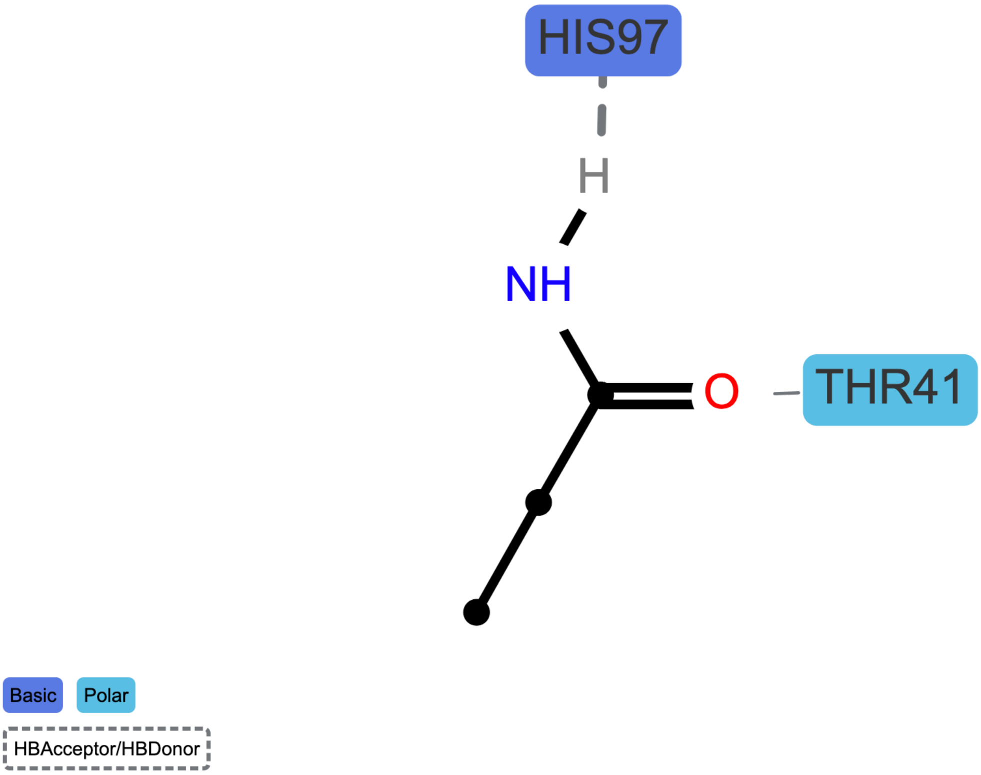

Hemoglobin (C93) - Cluster 2			
residue	number	interaction	occurrence
His	97	HB acceptor	0.90
Thr	41	HB donor	0.23

Figure S22: Modeled covalent adduct between acrylamide (ACR) and the target protein. (*Top*) Schematic representation of acrylamide and the hydrogen bonds (HBs) formed with the surrounding binding site residues. Residues are colored according to their physicochemical properties, as shown in the figure legend, and the corresponding HB frequency is represented through the width of the dashed line. (*Bottom*) Table indicating the target protein, reactive Cys and docking cluster considered, together with the list of detected protein-ligand HBs and their respective frequency.

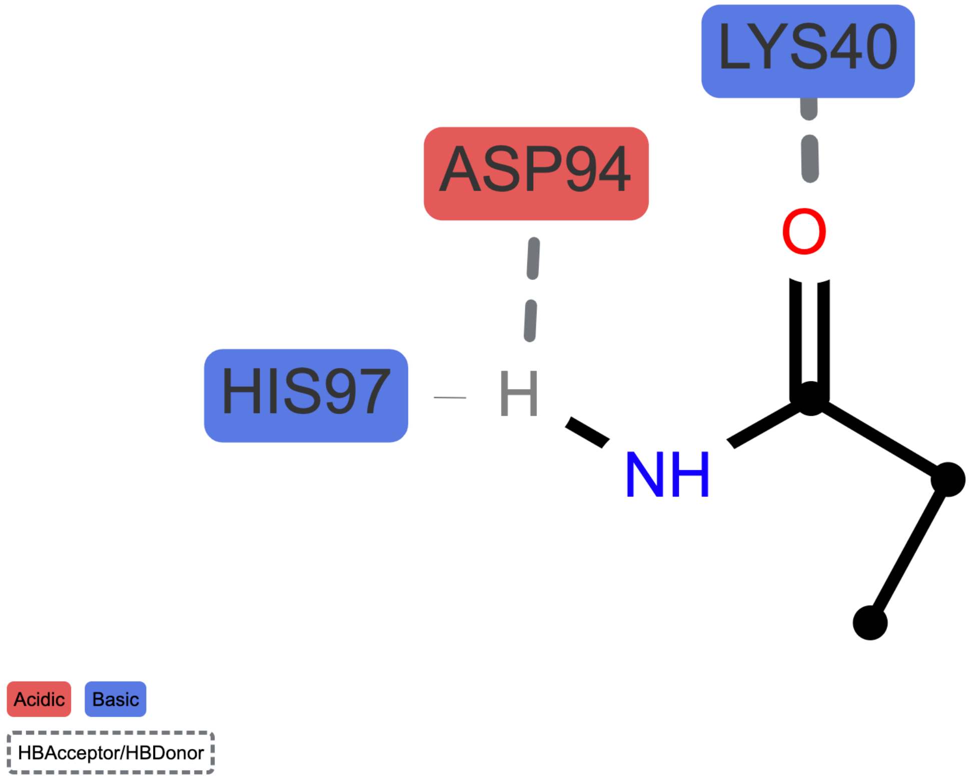

Hemoglobin (C93) - Cluster 3			
residue	number	interaction	occurrence
Asp	94	HB acceptor	0.89
His	97	HB acceptor	0.07
Lys	40	HB donor	0.93

Figure S23: Modeled covalent adduct between acrylamide (ACR) and the target protein. (*Top*) Schematic representation of acrylamide and the hydrogen bonds (HBs) formed with the surrounding binding site residues. Residues are colored according to their physicochemical properties, as shown in the figure legend, and the corresponding HB frequency is represented through the width of the dashed line. (*Bottom*) Table indicating the target protein, reactive Cys and docking cluster considered, together with the list of detected protein-ligand HBs and their respective frequency.

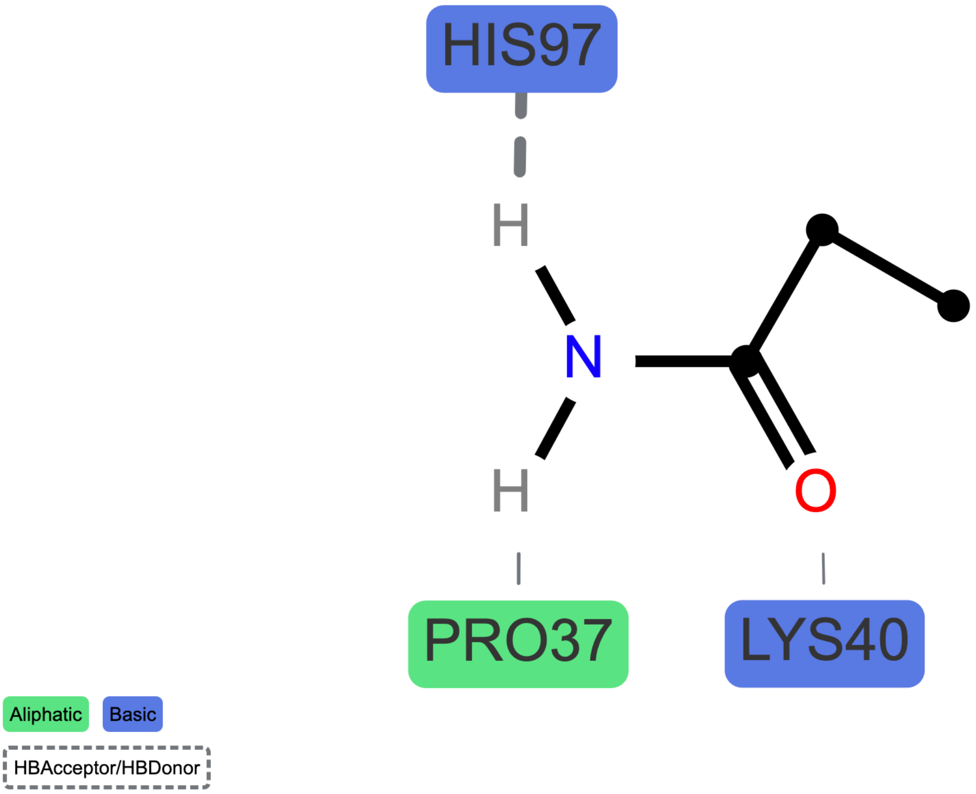

Hemoglobin (C93) - Cluster 4			
residue	number	interaction	occurrence
His	97	HB acceptor	0.68
Pro	37	HB acceptor	0.21
Lys	40	HB donor	0.16

Figure S24: Modeled covalent adduct between acrylamide (ACR) and the target protein. (*Top*) Schematic representation of acrylamide and the hydrogen bonds (HBs) formed with the surrounding binding site residues. Residues are colored according to their physicochemical properties, as shown in the figure legend, and the corresponding HB frequency is represented through the width of the dashed line. (*Bottom*) Table indicating the target protein, reactive Cys and docking cluster considered, together with the list of detected protein-ligand HBs and their respective frequency.

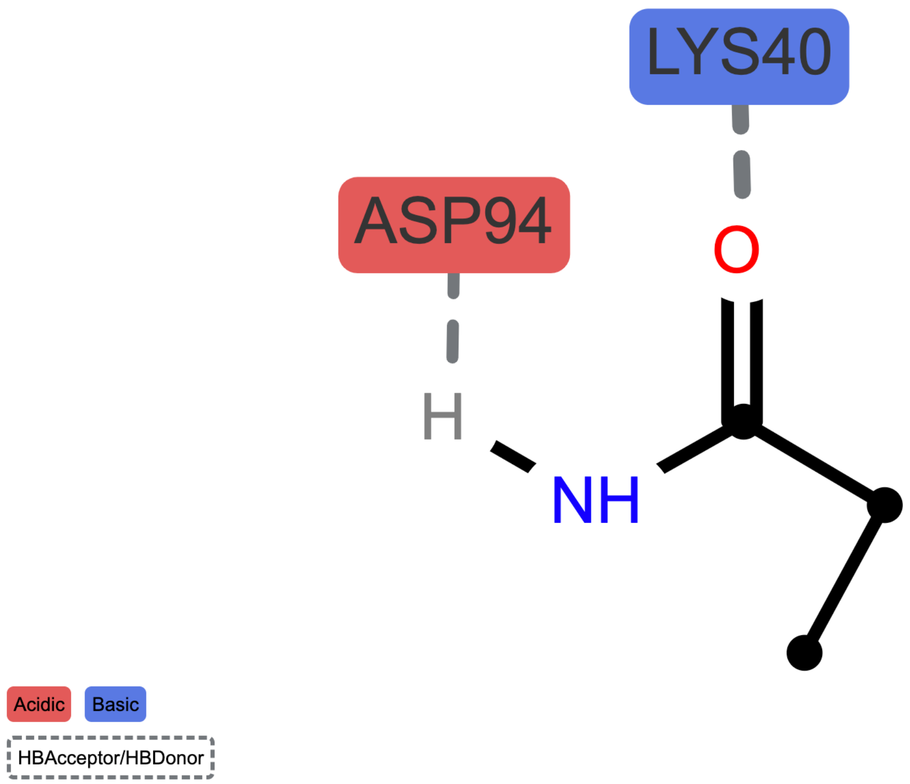

Hemoglobin (C93) - Cluster 5			
residue	number	interaction	occurrence
Asp	94	HB acceptor	0.75
Lys	40	HB donor	0.88

Figure S25: Modeled covalent adduct between acrylamide (ACR) and the target protein. (*Top*) Schematic representation of acrylamide and the hydrogen bonds (HBs) formed with the surrounding binding site residues. Residues are colored according to their physicochemical properties, as shown in the figure legend, and the corresponding HB frequency is represented through the width of the dashed line. (*Bottom*) Table indicating the target protein, reactive Cys and docking cluster considered, together with the list of detected protein-ligand HBs and their respective frequency.

1.3.7 NEM-sensitive factor (NSF)

Docking to NSF C264 rendered two main clusters (numbers 1 and 2) with Haddock scores within standard deviation of each other. Analysis of the protein-ligand interactions for cluster 1 did not identify any significant contact, whereas the interactions for cluster 2 are shown below.

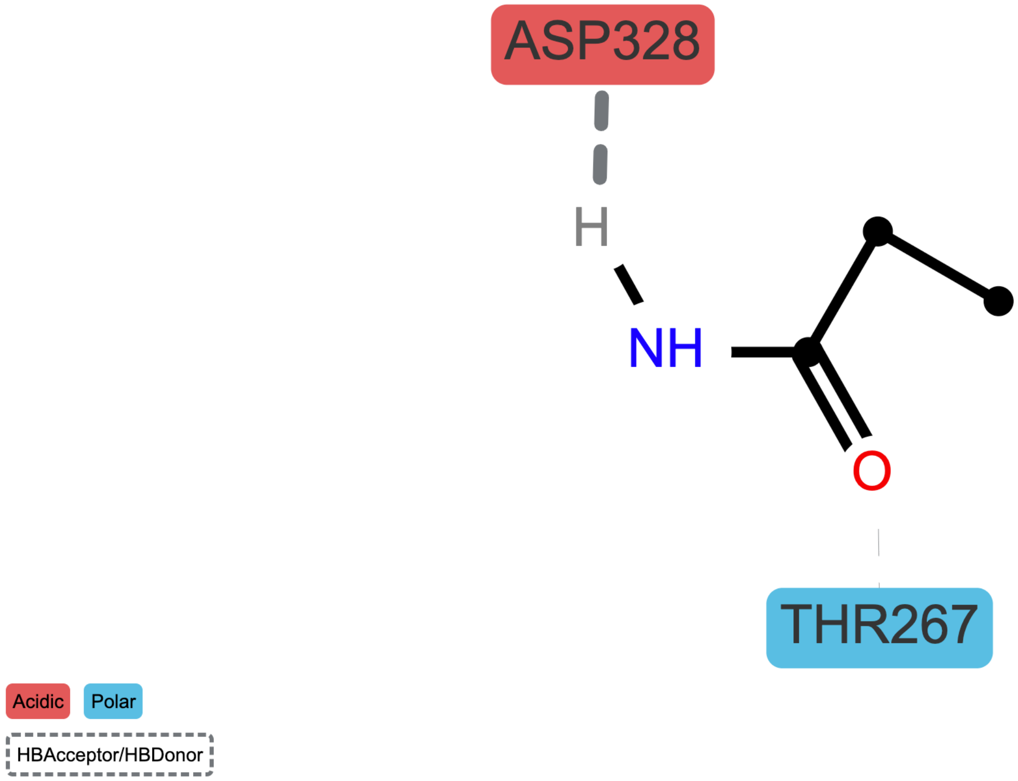

NEM-sensitive factor (C264) - Cluster 2			
residue	number	interaction	occurrence
Thr	267	HB donor	0.03
Asp	328	HB acceptor	0.88

Figure S26: Modeled covalent adduct between acrylamide (ACR) and the target protein. (*Top*) Schematic representation of acrylamide and the hydrogen bonds (HBs) formed with the surrounding binding site residues. Residues are colored according to their physicochemical properties, as shown in the figure legend, and the corresponding HB frequency is represented through the width of the dashed line. (*Bottom*) Table indicating the target protein, reactive Cys and docking cluster considered, together with the list of detected protein-ligand HBs and their respective frequency.

1.3.8 Vesicular proton ATPase (v-ATPase)

Covalent docking for C254 of v-ATPase yielded one main top cluster (number 1); the analysis of the protein-ligand interactions present in the docking poses belonging to that cluster is shown below. As mentioned in the main text, the (only slightly) favorable docking score of this top cluster (-0.1 a.u.) can be attributed to C254 being located in a loop segment of the Walker A motif (GAFG*C*GKT). This motif is involved in coordinating ATP binding and hydrolysis and hence exhibits large rearrangements during the v-ATPase conformational cycle (see Figure S26). Such structural changes cannot be sampled with covalent docking protocols, thus resulting in lower accuracy of the predicted docking poses.

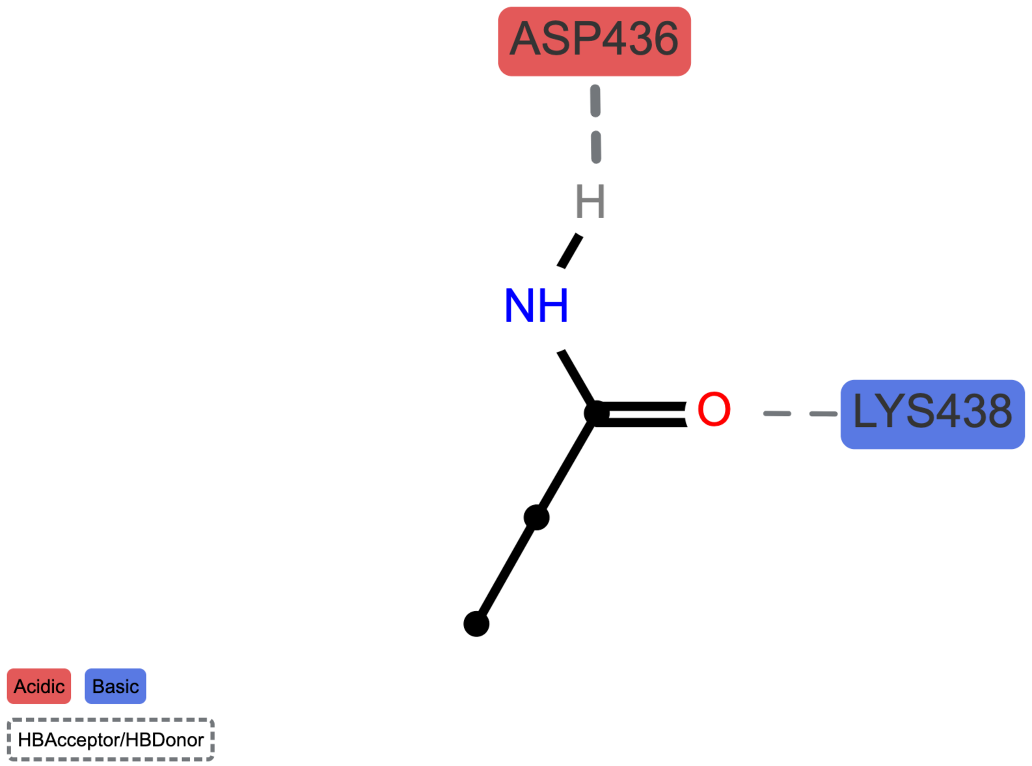

v-ATPase (C254) - Cluster 1			
residue	number	interaction	occurrence
Asp	436	HB acceptor	0.72
Lys	438	HB donor	0.36

Figure S27: Modeled covalent adduct between acrylamide (ACR) and the target protein. (*Top*) Schematic representation of acrylamide and the hydrogen bonds (HBs) formed with the surrounding binding site residues. Residues are colored according to their physicochemical properties, as shown in the figure legend, and the corresponding HB frequency is represented through the width of the dashed line. (*Bottom*) Table indicating the target protein, reactive Cys and docking cluster considered, together with the list of detected protein-ligand HBs and their respective frequency.

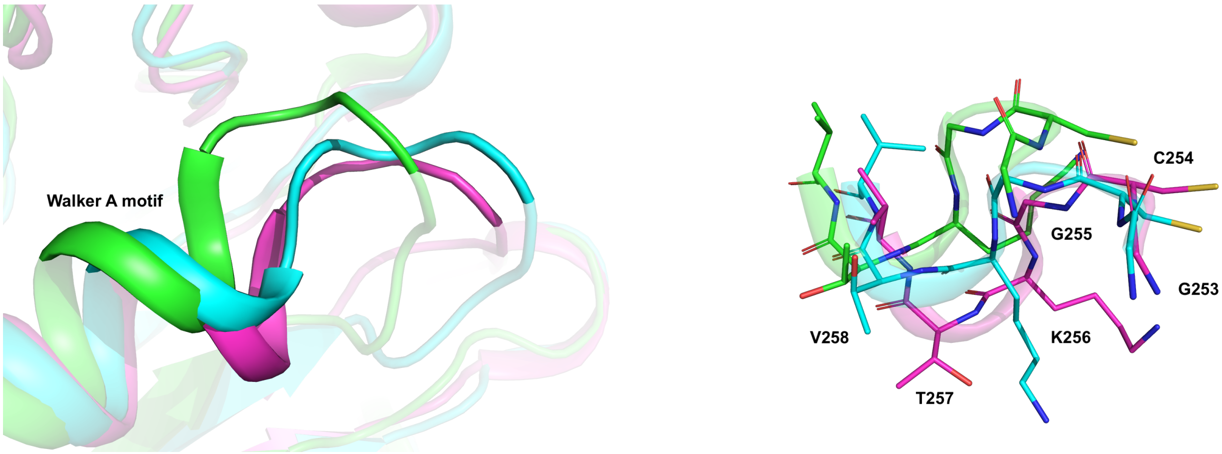

Figure S28: Flexibility of the v-ATPase Walker A motif. Structural superposition of crystallographic chains A (green), B (cyan) and C (magenta) of PDB structure 6WM2; the left and right panels display the secondary structures and the side chain positions, respectively, of the last five residues of the Walker A motif (G253-T257), including the reactive C254.

1.4 Proteins without reactive cysteine experimental information: Selection of candidate residues

Out of the 19 proteins in our dataset (Table S1), the specific Cys targeted by acrylamide is not known for eleven (see below; proteins are listed in alphabetical order). In order to narrow down the most promising candidates to be the reactive Cys, we first checked physicochemical properties and microenvironment, as well as conservation and post-translational modifications, for all the cysteines of the respective protein target (see section 2.2 in the main text). Below we present the outcome of these analyses, as well as the main results of the covalent docking calculations performed for each of the selected candidate Cys residues. For further details, we refer the reader to Supplementary Material 2, which includes the full report of the docking results. Here we show the protein-ligand interaction analysis of the docking poses of the top (best scored) cluster, as well as for additional clusters when their Haddock scores fall within standard deviation of the top cluster. If no interactions were detected by ProLIF (Bouysset and Fiorucci, 2021), no scheme is shown.

1.4.1 Alcohol Dehydrogenase (ADH)

Alcohol Dehydrogenase is an enzyme part of the ethanol metabolism that converts alcohols to aldehydes. ADH possesses multiple isoforms in humans which can be grouped into different sub-classes. The crystal structure with the highest resolution belongs to ADH1C, also known as ADH3 (see Table S1). Hence, the subsequent analyses were performed with this isoform.

An MSA of the seven human ADH sequences (Figure S29) revealed eleven conserved Cys. However, six of them are part of the two Zn^{2+} binding sites present in ADHs (Eklund et al., 1976), and thus were discarded as potential ACR binding sites. Out of the remaining five conserved cysteines, we selected C170 and C240 (ADH1C numbering) for our covalent docking tests. C170 is located near the enzyme active site and thus its modification is more likely to have an impact on the protein function. In addition, C240 in ADH appears to be the residue equivalent to C247 in GAPDH; the latter has been shown experimentally to be the target of ACR, though at high concentrations.

The results of the covalent docking for each of these two cysteines, using the aforementioned ADH structure, are reported below and in Supplementary Material 2. Based on the more favorable docking score for C240 ($-10.8\,\mathrm{a.u.}$) compared to C170 ($-4.2\,\mathrm{a.u.}$), we suggest that C240 might be the primary site of ACR modification in ADH.

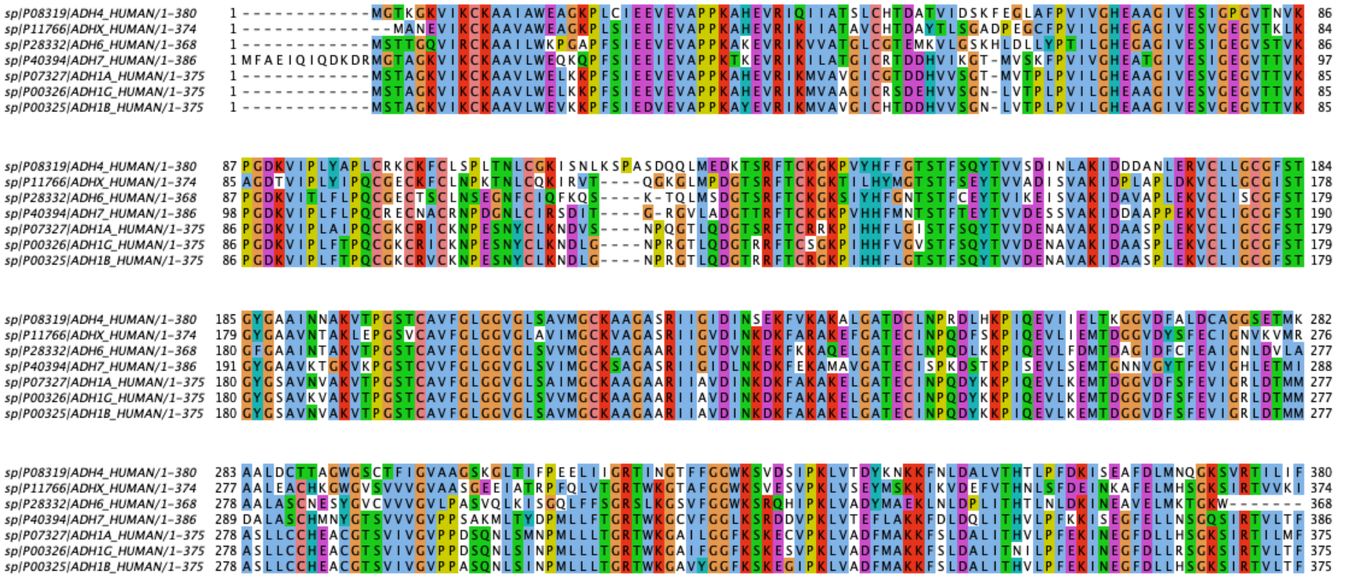

Figure S29: Multiple sequence alignment of human alcohol dehydrogenase isoforms, performed with the MAFFT webserver (Katoh et al., 2022). The Clustal color code was used, with hydrophobic residues in blue, positively charged in red, negatively charged in magenta, polar in green and aromatic in cyan. Special residues are shown in orange (glycine), yellow (proline) and pink (csyteine) and non-conserved residues are in white.

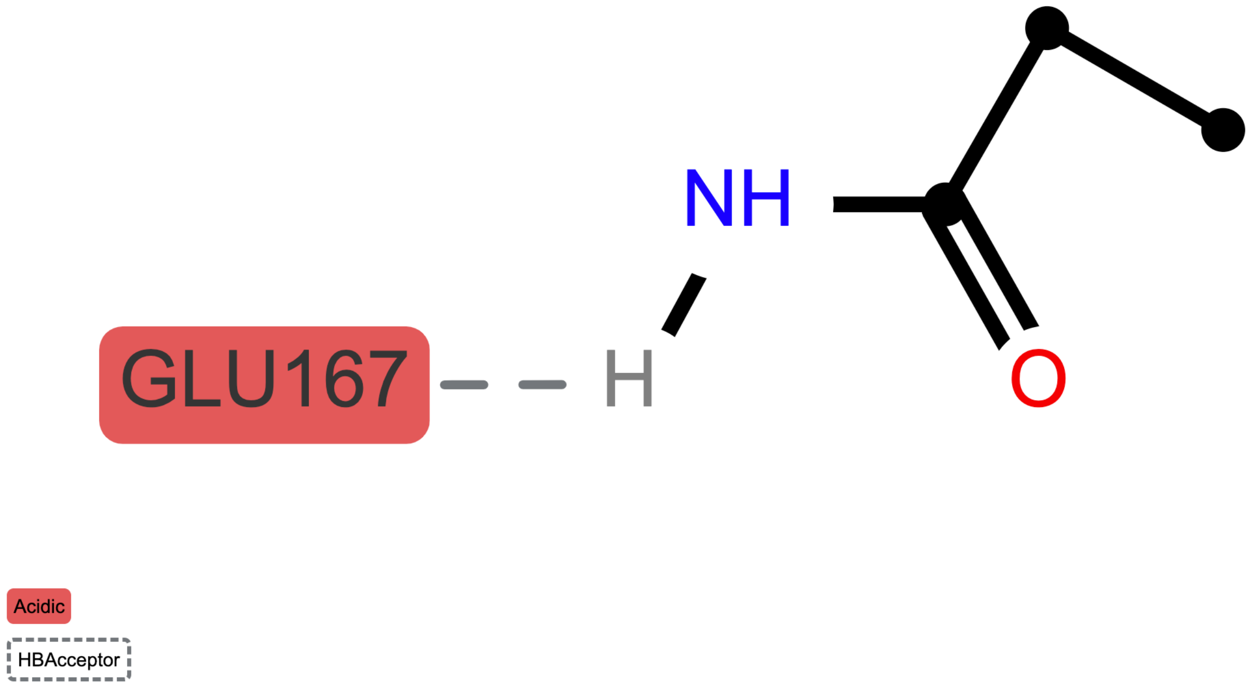

Alcohol Dehydrogenase (C170) - Cluster 1			
residue	number	interaction	occurrence
Glu	167	HB acceptor	0.44

Figure S30: Modeled covalent adduct between acrylamide (ACR) and the target protein. (*Top*) Schematic representation of acrylamide and the hydrogen bonds (HBs) formed with the surrounding binding site residues. Residues are colored according to their physicochemical properties, as shown in the figure legend, and the corresponding HB frequency is represented through the width of the dashed line. (*Bottom*) Table indicating the target protein, reactive Cys and docking cluster considered, together with the list of detected protein-ligand HBs and their respective frequency.

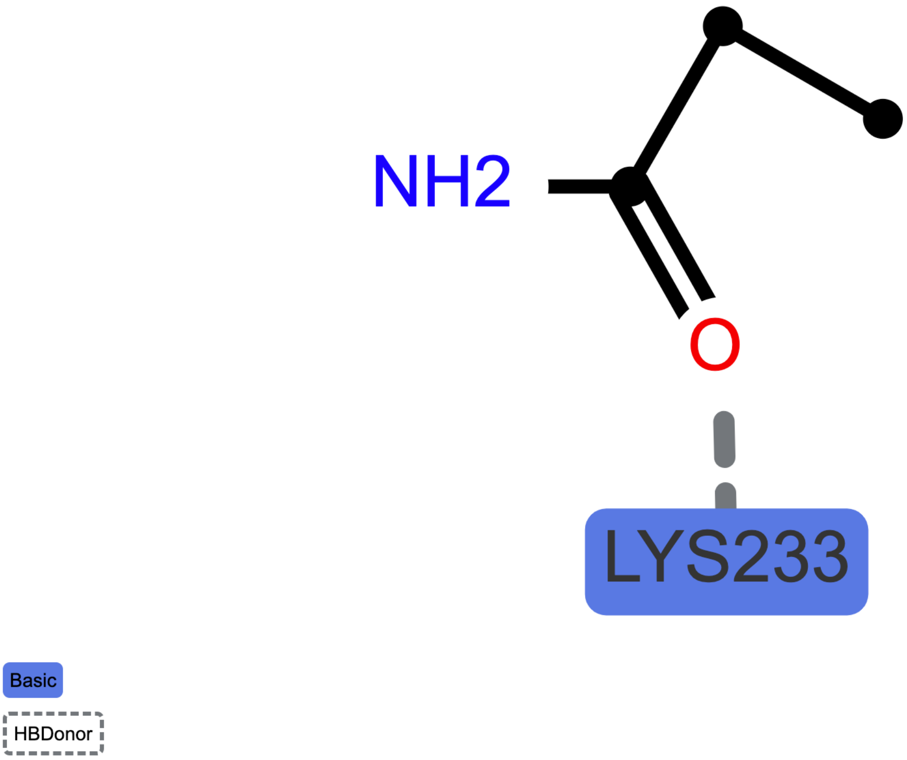

Alcohol Dehydrogenase (C240) - Cluster 1			
residue	number	interaction	occurrence
Lys	233	HB donor	1.00

Figure S31: Modeled covalent adduct between acrylamide (ACR) and the target protein. (*Top*) Schematic representation of acrylamide and the hydrogen bonds (HBs) formed with the surrounding binding site residues. Residues are colored according to their physicochemical properties, as shown in the figure legend, and the corresponding HB frequency is represented through the width of the dashed line. (*Bottom*) Table indicating the target protein, reactive Cys and docking cluster considered, together with the list of detected protein-ligand HBs and their respective frequency.

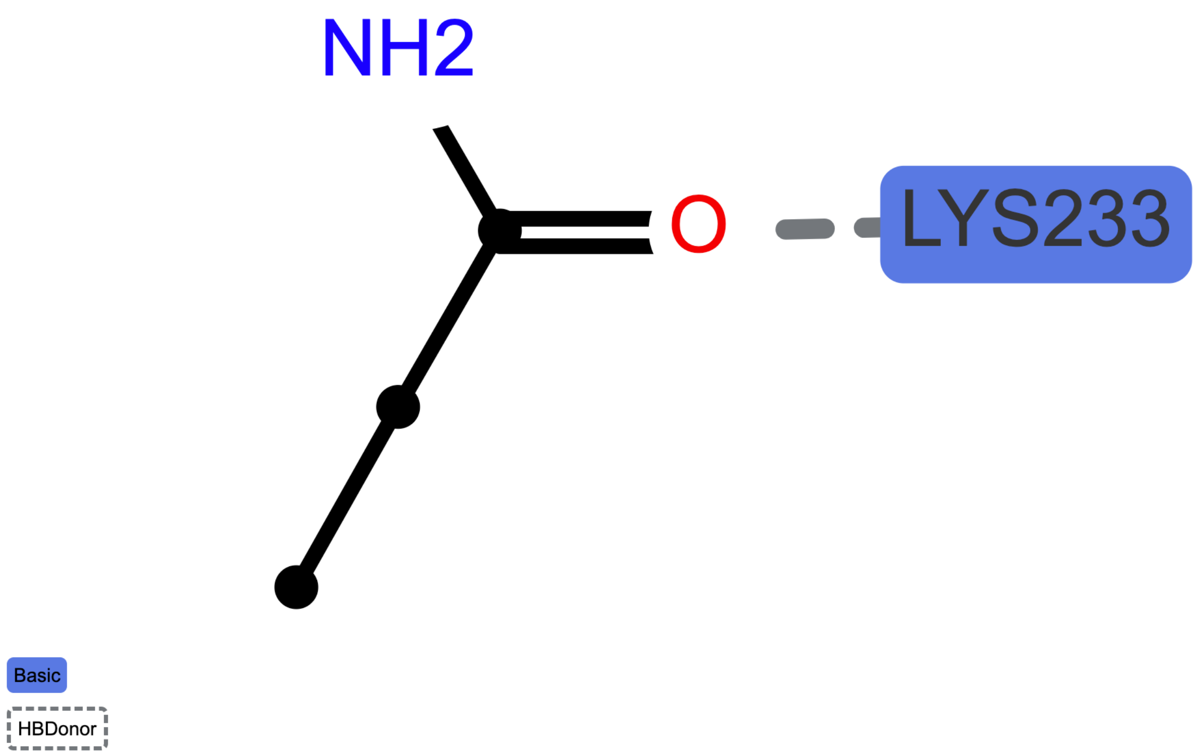

Alcohol Dehydrogenase (C240) - Cluster 2			
residue	number	interaction	occurrence
Lys	233	HB donor	0.86

Figure S32: Modeled covalent adduct between acrylamide (ACR) and the target protein. (*Top*) Schematic representation of acrylamide and the hydrogen bonds (HBs) formed with the surrounding binding site residues. Residues are colored according to their physicochemical properties, as shown in the figure legend, and the corresponding HB frequency is represented through the width of the dashed line. (*Bottom*) Table indicating the target protein, reactive Cys and docking cluster considered, together with the list of detected protein-ligand HBs and their respective frequency.

1.4.2 Aldolase

Aldolase (or fructose-bisphosphate aldolase) is a key enzyme within the glycolysis pathway. It is responsible for splitting fructose 1,6-bisphosphate into dihydroxyacetone phosphate (DHAP) and glyceraldehyde 3-phosphate (GADP). Aldolase has three isoforms: aldolase A (expressed in muscle), aldolase B (liver) and aldolase C (brain). *In vitro* experiments on rabbit muscle aldolase showed a multiphasic inactivation of aldolase by acrylamide (Dobryszycki et al., 1999a); however, there is no mutagenesis data pinpointing the Cys residues targeted by ACR. Hence, we first checked cysteine conservation by generating a multiple sequence alignment of human and rabbit aldolase isoforms (Figure S33). We found that C134, C177, C239 and C289 are conserved and thus can be potential candidates for the reactive Cys targeted by ACR and/or have functional relevance.

Next, we used the crystal structure of human liver aldolase (i.e. the highest resolution crystal structure) to perform ACR covalent dockings for each of these cysteines. As shown in Figure S33, this isoform possesses an additional cysteine residue at position 268, which is solvent exposed and thus was also submitted to our docking workflow. As mentioned above, experimental studies showed that aldolase inactivation is dependent on both ACR concentration and time of incubation (Dobryszycki et al., 1999a). This rather complex pattern of inactivation indicates that acrylamide could form multiple covalent adducts at several Cys sites before aldolase activity is completely abolished. Therefore, in this case it is particularly relevant to consider all possible Cys candidates for covalent docking.

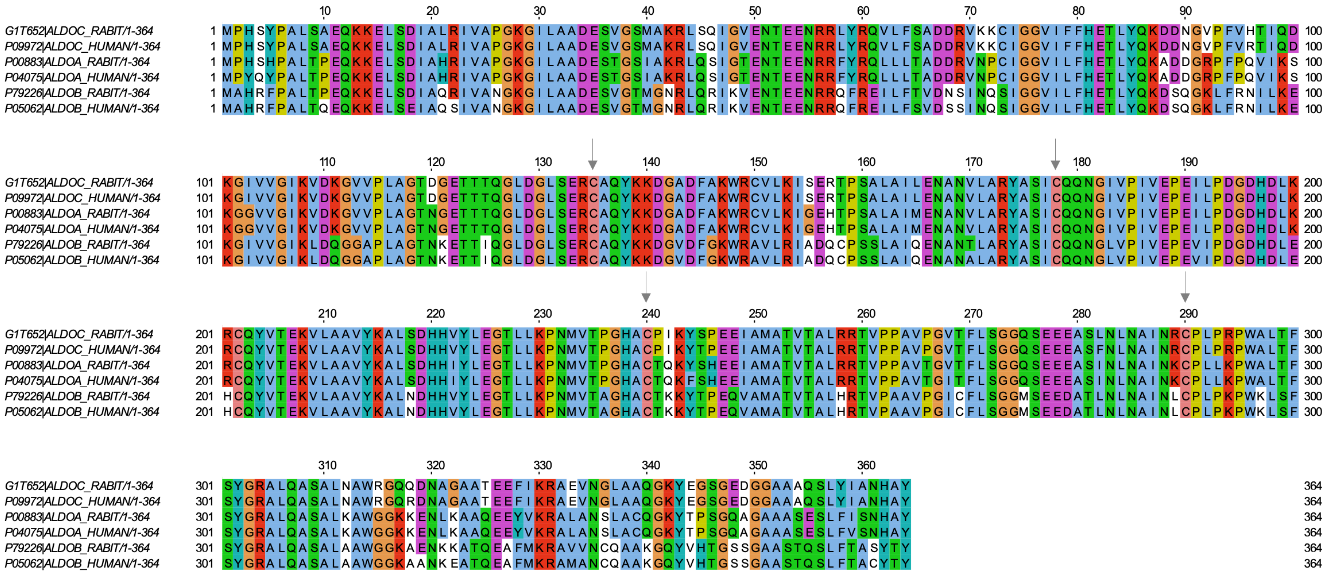

Figure S33: Multiple sequence alignment of human and rabbit aldolase isoforms, performed with the MAFFT webserver (Katoh et al., 2022). Aldolase isoforms A (muscle), B (liver) and C (brain) were included. The same color code as in Figure S29 was used.

Our computational results showed that C134, C239, C268 and C289 exhibit similar favorable docking scores for their top clusters, i.e. -26.1 a.u., -23.1 a.u., -29.4 a.u. and -24.1 a.u., respectively (see Supplementary Material 2). Below we report the protein-ligand interactions for each of the Cys-ACR covalent adducts. In contrast, the less favorable docking score for C177 (-9.7 a.u.), as well as the lack of a properly formed S-C bond between C177 and ACR in our models, suggest that modification of this particular residue is less likely.

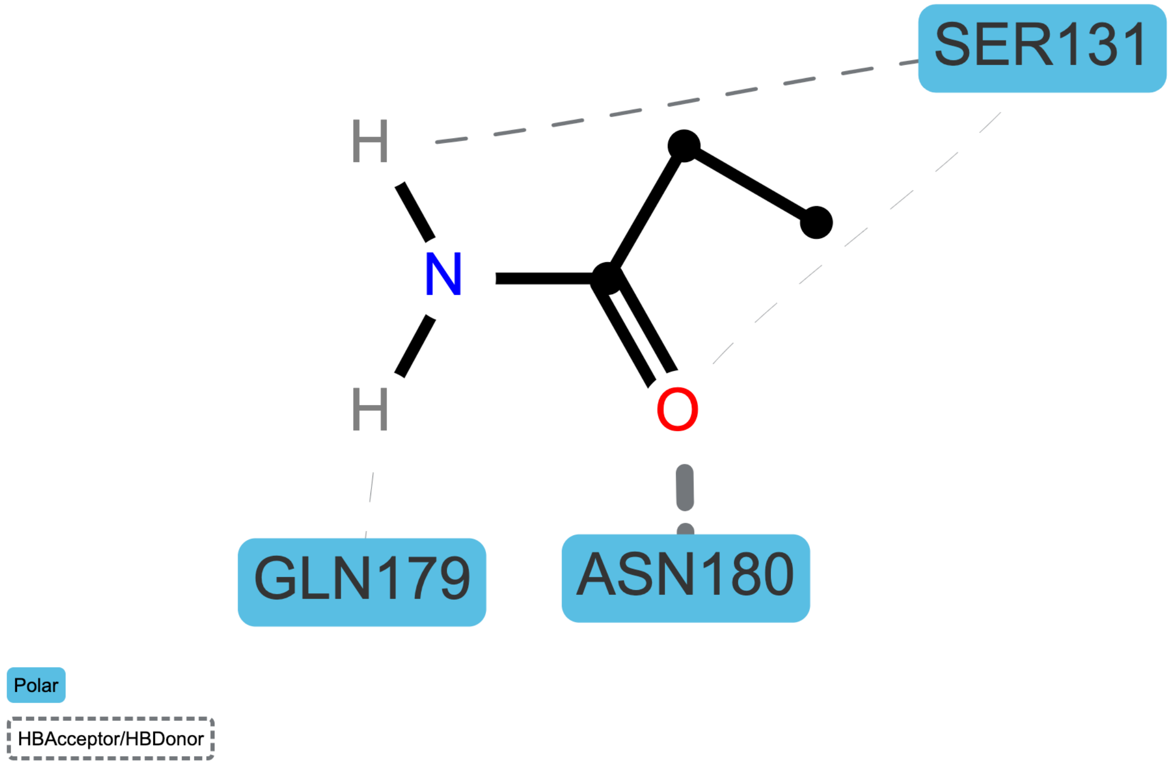

Aldolase (C134) - Cluster 1			
residue	number	interaction	occurrence
Ser	131	HB donor	0.03
Ser	131	HB acceptor	0.21
Gln	179	HB acceptor	0.05
Asn	180	HB donor	1.00

Figure S34: Modeled covalent adduct between acrylamide (ACR) and the target protein. (*Top*) Schematic representation of acrylamide and the hydrogen bonds (HBs) formed with the surrounding binding site residues. Residues are colored according to their physicochemical properties, as shown in the figure legend, and the corresponding HB frequency is represented through the width of the dashed line. (*Bottom*) Table indicating the target protein, reactive Cys and docking cluster considered, together with the list of detected protein-ligand HBs and their respective frequency.

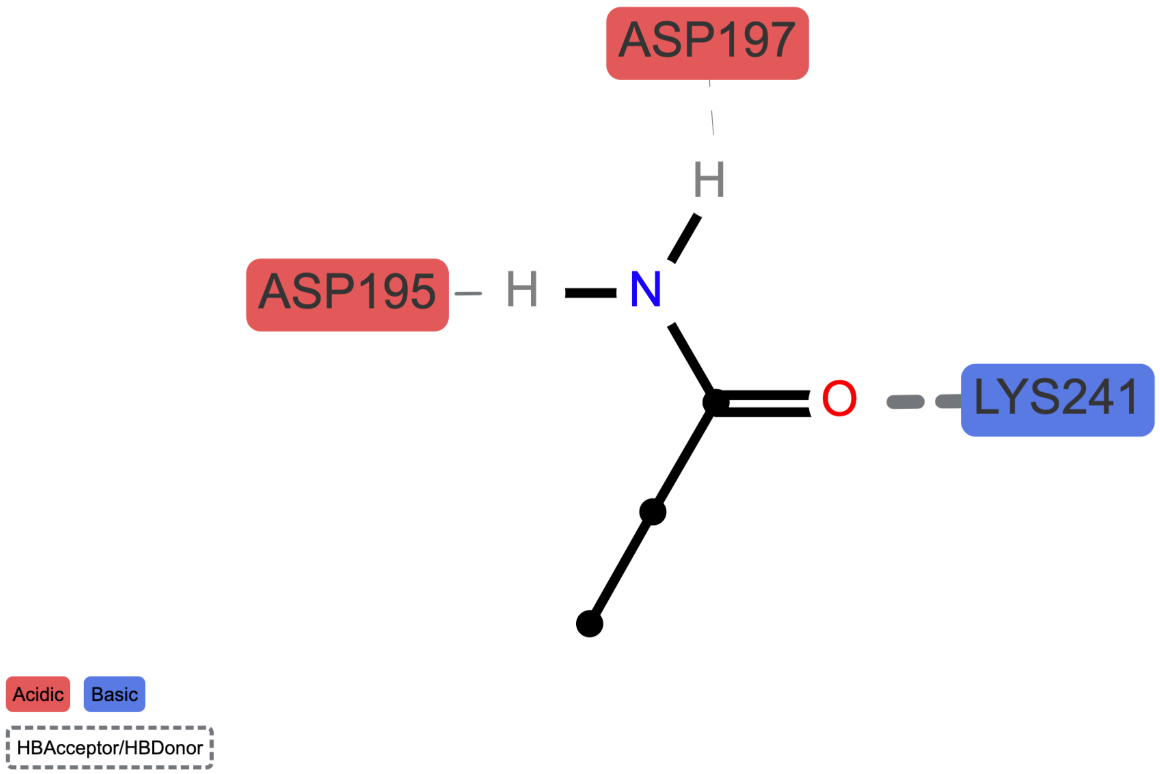

Aldolase (C239) - Cluster 1			
residue	number	interaction	occurrence
Asp	195	HB acceptor	0.24
Asp	197	HB acceptor	0.06
Lys	241	HB donor	0.96

Figure S35: Modeled covalent adduct between acrylamide (ACR) and the target protein. (*Top*) Schematic representation of acrylamide and the hydrogen bonds (HBs) formed with the surrounding binding site residues. Residues are colored according to their physicochemical properties, as shown in the figure legend, and the corresponding HB frequency is represented through the width of the dashed line. (*Bottom*) Table indicating the target protein, reactive Cys and docking cluster considered, together with the list of detected protein-ligand HBs and their respective frequency.

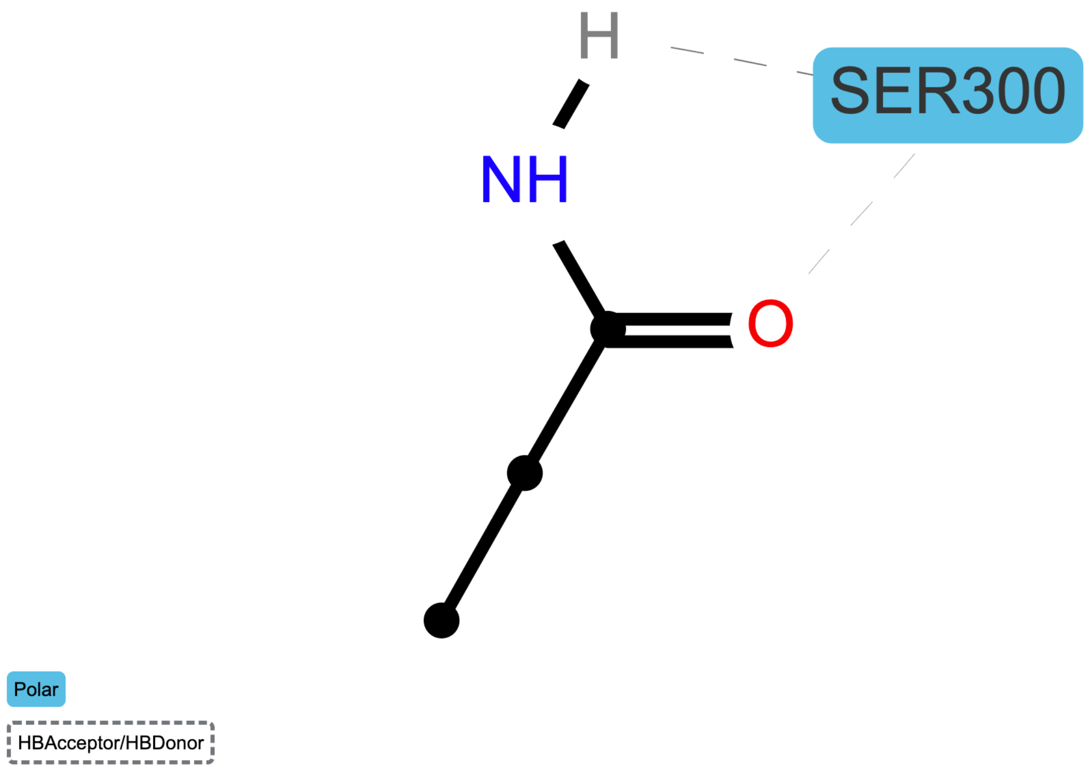

Aldolase (C268) - Cluster 1			
residue	number	interaction	occurrence
Ser	300	HB donor	0.03
Ser	300	HB acceptor	0.09

Figure S36: Modeled covalent adduct between acrylamide (ACR) and the target protein. (*Top*) Schematic representation of acrylamide and the hydrogen bonds (HBs) formed with the surrounding binding site residues. Residues are colored according to their physicochemical properties, as shown in the figure legend, and the corresponding HB frequency is represented through the width of the dashed line. (*Bottom*) Table indicating the target protein, reactive Cys and docking cluster considered, together with the list of detected protein-ligand HBs and their respective frequency.

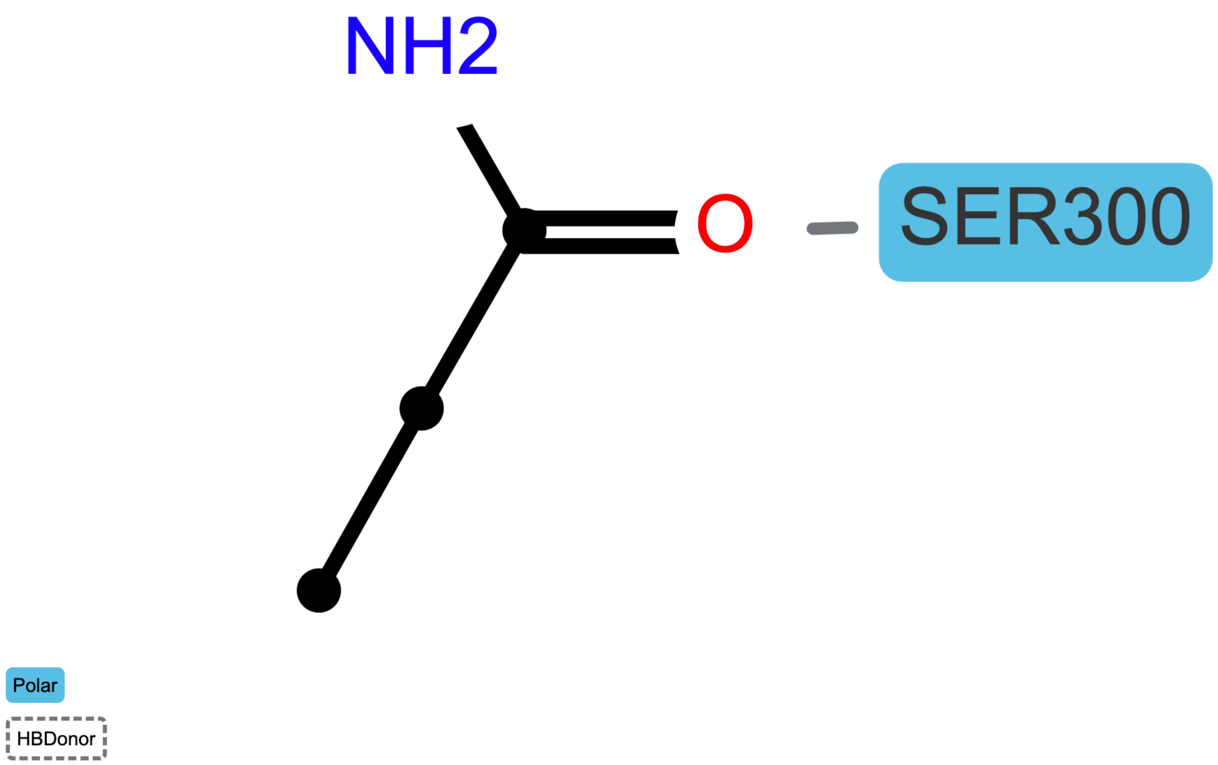

Aldolase (C268) - Cluster 2			
residue	number	interaction	occurrence
Ser	300	HB donor	0.52

Figure S37: Modeled covalent adduct between acrylamide (ACR) and the target protein. (*Top*) Schematic representation of acrylamide and the hydrogen bonds (HBs) formed with the surrounding binding site residues. Residues are colored according to their physicochemical properties, as shown in the figure legend, and the corresponding HB frequency is represented through the width of the dashed line. (*Bottom*) Table indicating the target protein, reactive Cys and docking cluster considered, together with the list of detected protein-ligand HBs and their respective frequency.

1.4.3 Enolase

Like aldolase and GAPDH, enolase is an enzyme part of the glycolysis pathway and is responsible for the conversion of 2-phosphoglycerate to phosphoenolpyruvate. Different isoforms of enolase exist, assembled as either homo- or heterodimers.

In order to pinpoint possible candidates for subsequent covalent docking, we first identified solvent exposed Cys residues. Out of the six conserved cysteines present in enolase (C118, C336, C356, C338, C356, C388 and C398, see Figure S38), only the last two are significantly solvent exposed and thus accessible to acrylamide. Hence, C388 and C398 were considered for the subsequent covalent docking calculations.

Based on the docking scores obtained (-36.3 and -39.0 a.u.) for C388 and C398, respectively), we suggest that both cysteines can potentially be targeted by acrylamide. Modification of C388 by ACR is supported by experimental studies showing that this cysteine undergoes modification under electrophile-induced oxidative stress, resulting in enzyme inactivation (Ishii and Uchida, 2004). In addition, ACR modification of C398 could further contribute to the experimentally observed protein inhibition (Howland et al., 1980) by altering the quaternary structure of the enzyme, as C398 is located at the interface between two enolase subunits. In this regard, this example highlights the importance to map the location of the candidate reactive Cys on the protein 3D structure in order to establish a connection between ACR modification and its impact on protein function.

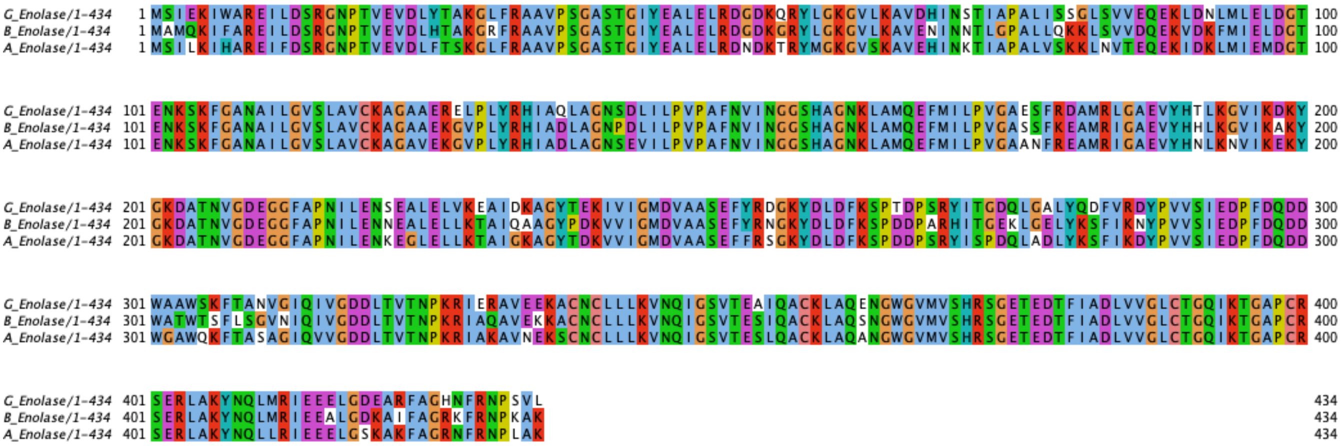

Figure S38: Multiple sequence alignment of human enolase isoforms (α, β and γ) generated using the MAFFT webserver (Katoh et al., 2022). The same color code as in Figure S29 was used.

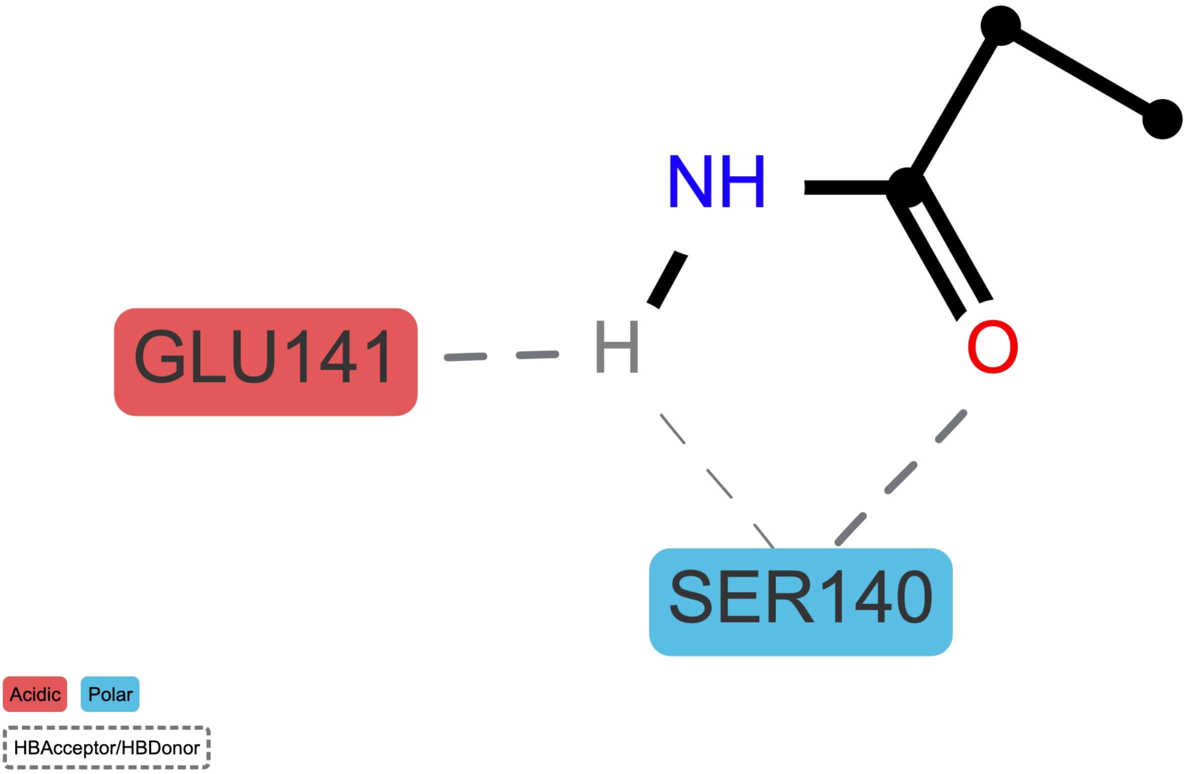

Enolase (C388) - Cluster 1			
residue	number	interaction	occurrence
Ser	140	HB donor	0.36
Ser	140	HB acceptor	0.13
Glu	141	HB acceptor	0.39

Figure S39: Modeled covalent adduct between acrylamide (ACR) and the target protein. (*Top*) Schematic representation of acrylamide and the hydrogen bonds (HBs) formed with the surrounding binding site residues. Residues are colored according to their physicochemical properties, as shown in the figure legend, and the corresponding HB frequency is represented through the width of the dashed line. (*Bottom*) Table indicating the target protein, reactive Cys and docking cluster considered, together with the list of detected protein-ligand HBs and their respective frequency.

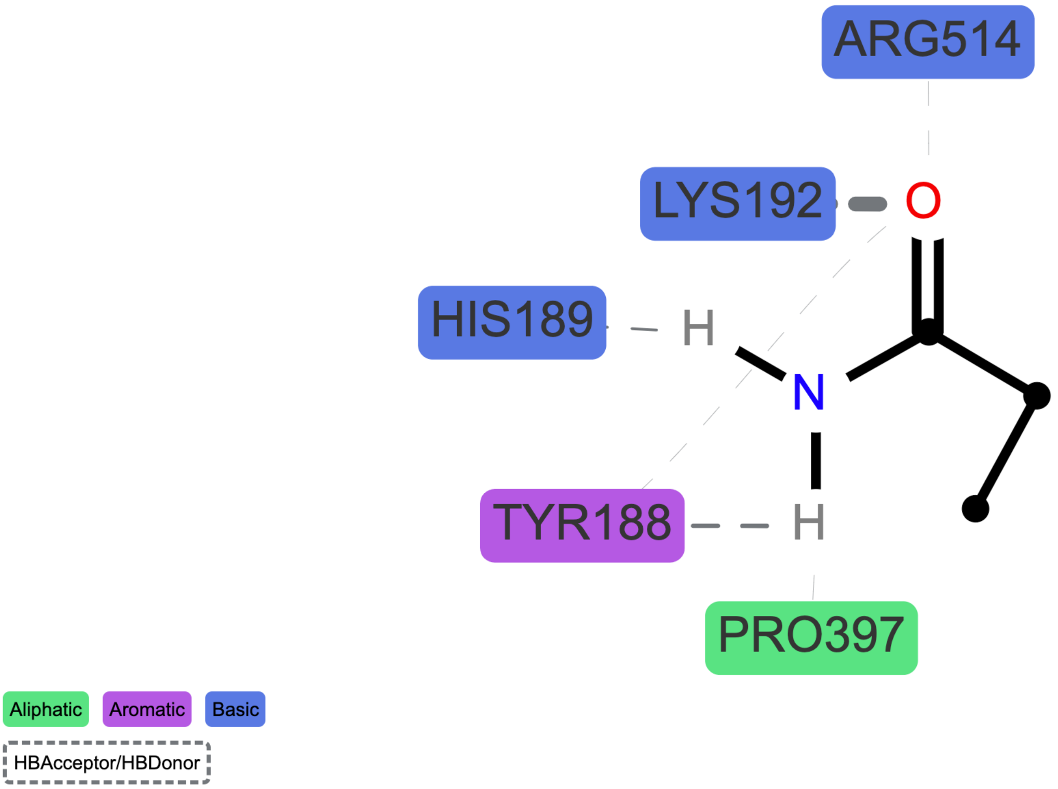

Enolase (C398) - Cluster 1			
residue	number	interaction	occurrence
Tyr	188	HB donor	0.01
Tyr	188	HB acceptor	0.31
His	189	HB acceptor	0.19
Lys	192	HB donor	1.00
Pro	397	HB acceptor	0.01
Arg	514	HB donor	0.03

Figure S40: Modeled covalent adduct between acrylamide (ACR) and the target protein. (*Top*) Schematic representation of acrylamide and the hydrogen bonds (HBs) formed with the surrounding binding site residues. Residues are colored according to their physicochemical properties, as shown in the figure legend, and the corresponding HB frequency is represented through the width of the dashed line. (*Bottom*) Table indicating the target protein, reactive Cys and docking cluster considered, together with the list of detected protein-ligand HBs and their respective frequency.

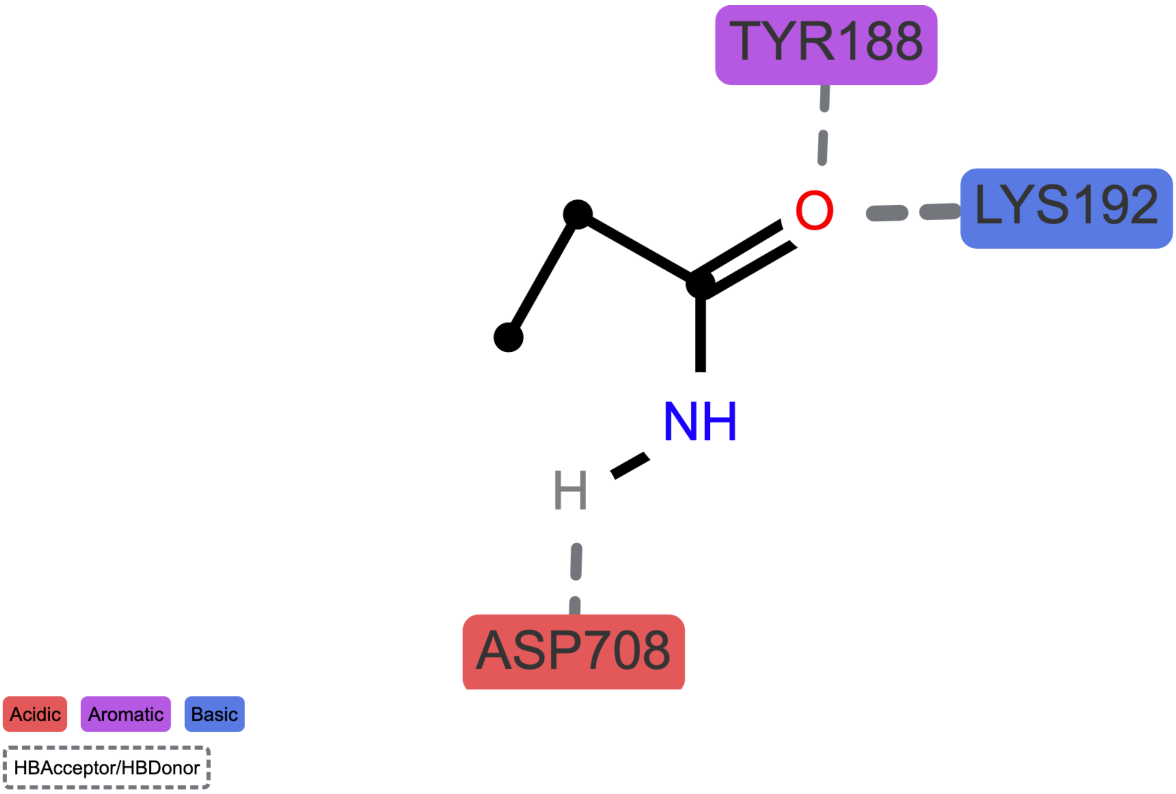

Enolase (C398) - Cluster 2			
residue	number	interaction	occurrence
Tyr	188	HB donor	0.60
Lys	192	HB donor	1.00
Asp	708	HB acceptor	0.79

Figure S41: Modeled covalent adduct between acrylamide (ACR) and the target protein. (*Top*) Schematic representation of acrylamide and the hydrogen bonds (HBs) formed with the surrounding binding site residues. Residues are colored according to their physicochemical properties, as shown in the figure legend, and the corresponding HB frequency is represented through the width of the dashed line. (*Bottom*) Table indicating the target protein, reactive Cys and docking cluster considered, together with the list of detected protein-ligand HBs and their respective frequency.

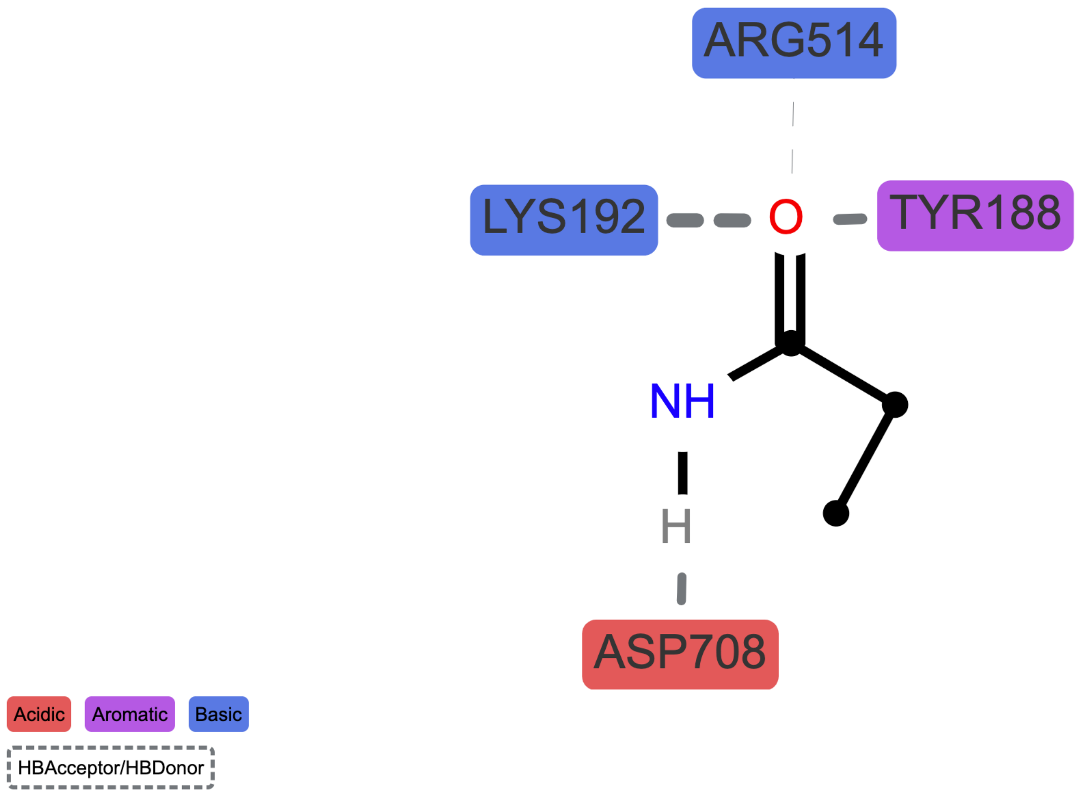

Enolase (C398) - Cluster 3			
residue	number	interaction	occurrence
Tyr	188	HB donor	0.76
Lys	192	HB donor	1.00
Arg	514	HB donor	0.05
Asp	708	HB acceptor	0.70

Figure S42: Modeled covalent adduct between acrylamide (ACR) and the target protein. (*Top*) Schematic representation of acrylamide and the hydrogen bonds (HBs) formed with the surrounding binding site residues. Residues are colored according to their physicochemical properties, as shown in the figure legend, and the corresponding HB frequency is represented through the width of the dashed line. (*Bottom*) Table indicating the target protein, reactive Cys and docking cluster considered, together with the list of detected protein-ligand HBs and their respective frequency.

1.4.4 Estrogen Receptor

Estrogen receptors are nuclear receptors initially identified as binding female steroids of the estrogen group and regulating pathways related to sexual maturity. Alterations of this tightly regulated hormonal balance can lead to pathological side effects, such as tumor formation.

Estrogen receptors have been proposed as potential targets of acrylamide based on the experimental observation that acrylamide induces misregulation of the hormone balance (Hogervorst et al., 2009). Moreover, human estrogen receptors contain conserved cysteine-rich binding domains that can act as reactive sites for ACR. In this regard, mutagenesis data has shown C381 and C530 to be involved in covalent hormone binding (Aliau et al., 1999) and thus these two cysteines could potentially form covalent adducts also with acrylamide.

Both residues showed favorable docking scores for their top cluster, -36.0 and $-22.6\,\mathrm{a.u.}$ for C381 and C530, respectively. Considering the more favorable docking score for C381, the presence of more protein-ligand HBs and a slightly lower pK_a value of 9.9 (compared to >12 of C530), it is reasonable to assume that C381 is the primary site of ACR modification. However, we cannot discard that C530 could still be modified by acrylamide at higher concentrations.

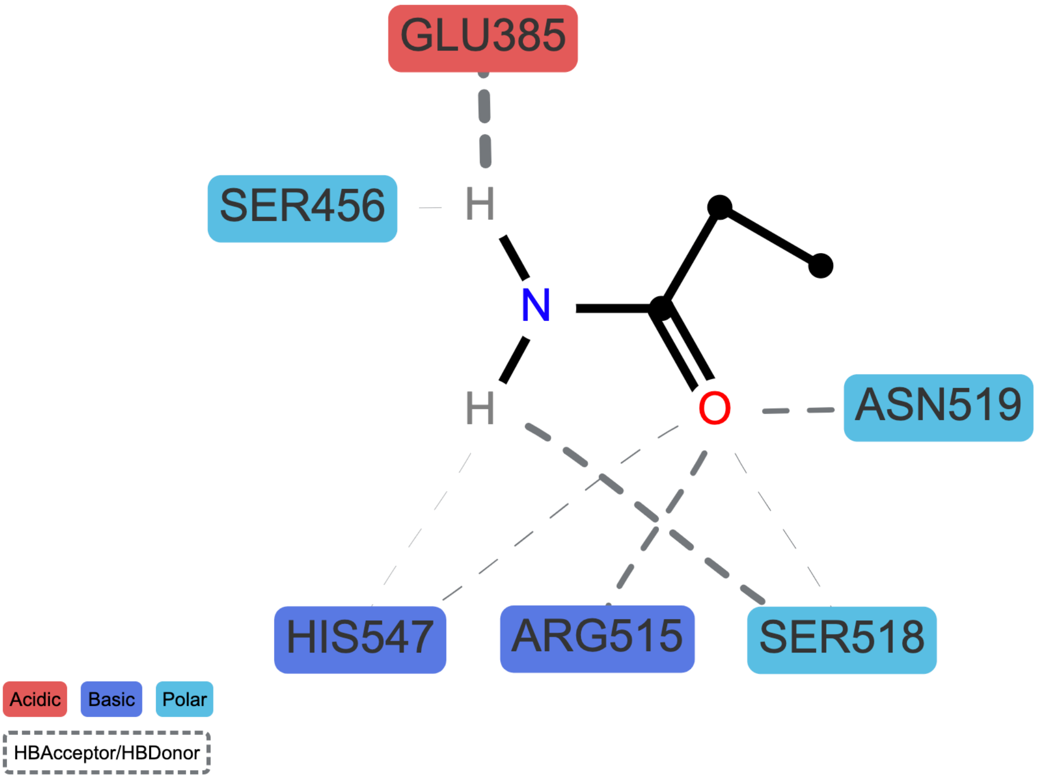

PDB ID: 1ERE - Estrogen Receptor (C381) - Cluster 1			
residue	number	interaction	occurrence
Glu	385	HB acceptor	0.94
Ser	456	HB acceptor	0.02
Arg	515	HB donor	0.44
Ser	518	HB donor	0.06
Ser	518	HB acceptor	0.54
Asn	519	HB donor	0.39
His	547	HB donor	0.11
His	547	HB acceptor	0.02

Figure S43: Modeled covalent adduct between acrylamide (ACR) and the target protein. (*Top*) Schematic representation of acrylamide and the hydrogen bonds (HBs) formed with the surrounding binding site residues. Residues are colored according to their physicochemical properties, as shown in the figure legend, and the corresponding HB frequency is represented through the width of the dashed line. (*Bottom*) Table indicating the target protein, reactive Cys and docking cluster considered, together with the list of detected protein-ligand HBs and their respective frequency.

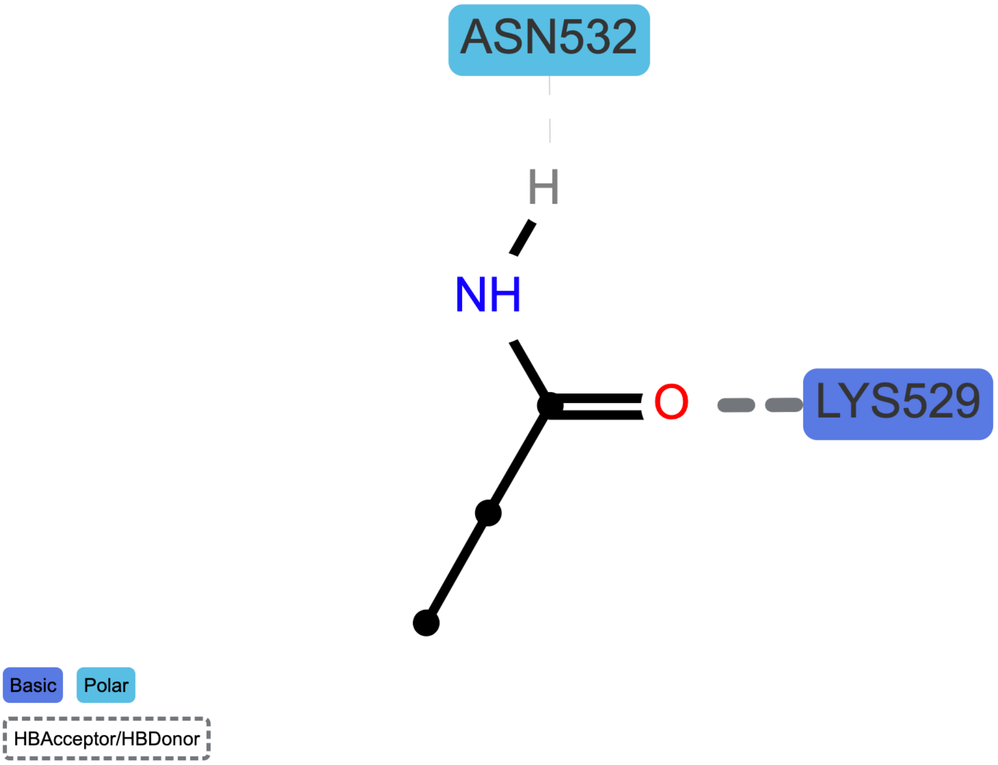

[PDB ID: 1ERE] Estrogen Receptor (C530) - Cluster 1			
residue	number	interaction	occurrence
Lys	529	HB donor	0.99
Asn	532	HB acceptor	0.02

Figure S44: Modeled covalent adduct between acrylamide (ACR) and the target protein. (*Top*) Schematic representation of acrylamide and the hydrogen bonds (HBs) formed with the surrounding binding site residues. Residues are colored according to their physicochemical properties, as shown in the figure legend, and the corresponding HB frequency is represented through the width of the dashed line. (*Bottom*) Table indicating the target protein, reactive Cys and docking cluster considered, together with the list of detected protein-ligand HBs and their respective frequency.

1.4.5 Immunoglobulins (Igs) G1 H Nie and kappa light chain

Acrylamide modification of two immunoglobulins was identified through nano liquid chromatography combined with tandem mass spectrometry (Feng and Lu, 2011b). Mapping of the modified peptides against the corresponding full protein sequences pinpointed C395 (immunoglobulin G1 H Nie) and C134 (immunoglobulin kappa light chain). Inspection of the respective protein structures (see Table S1) showed that both residues are involved in disulfide bridges. However, free thiols and chemical modification of disulfide bridges have been detected in other Igs (Liu and May, 2012). Therefore, we performed covalent dockings for each of these cysteines, assuming that either they are reversibly reduced under certain conditions or acrylamide is able to break and modify the corresponding disulfide bridge.

As shown in Supplementary Material 2, docking to C395 of immunoglobulin G1 H Nie showed one main cluster (number 1), whereas docking to C134 of the immunoglobulin kappa light chain rendered 4 clusters with Haddock scores within standard deviation of top (best scored) cluster. Below we report the protein-ligand interactions of those clusters for which HBs were detected. We speculate that ACR modification of the aforementioned cysteines will affect protein structure and stability of these two Igs, since it removes disulfide bridges.

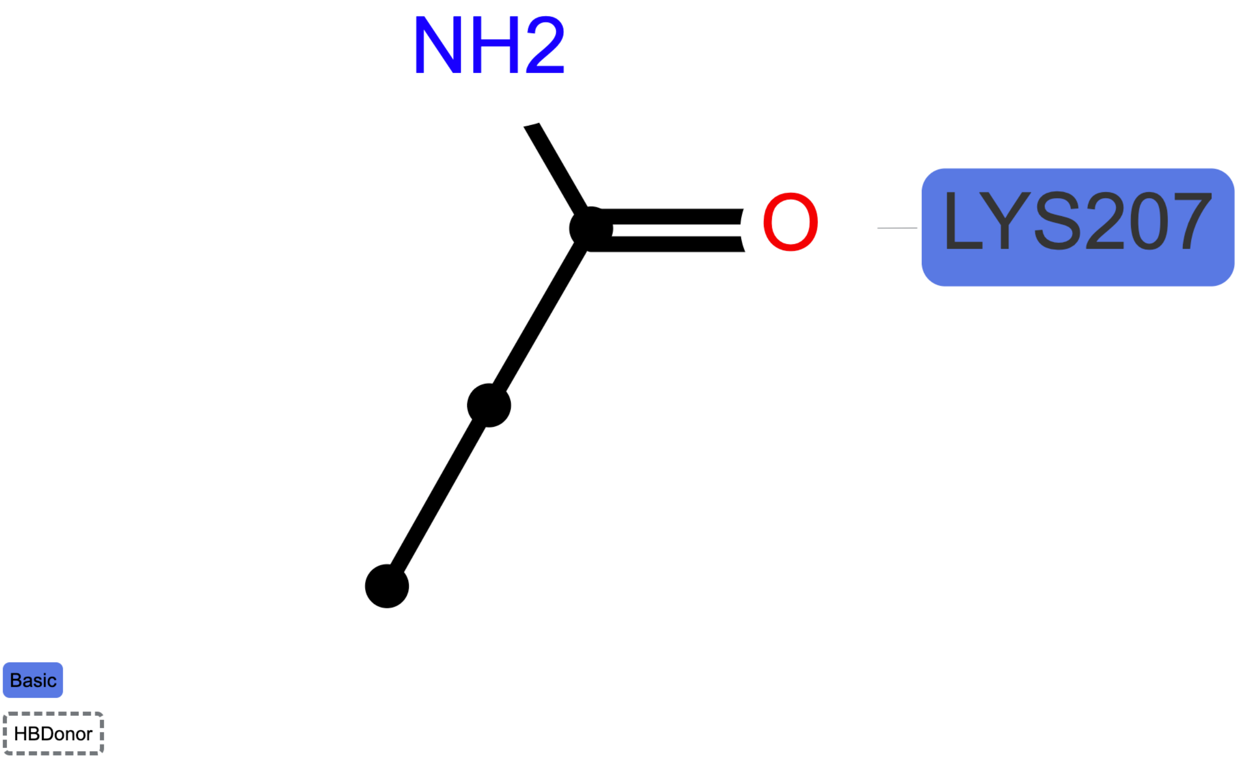

Immunoglobin Kappa Light Chain (C134) - Cluster 1			
residue	number	interaction	occurrence
Lys	207	HB donor	0.03

Figure S45: Modeled covalent adduct between acrylamide (ACR) and the target protein. (*Top*) Schematic representation of acrylamide and the hydrogen bonds (HBs) formed with the surrounding binding site residues. Residues are colored according to their physicochemical properties, as shown in the figure legend, and the corresponding HB frequency is represented through the width of the dashed line. (*Bottom*) Table indicating the target protein, reactive Cys and docking cluster considered, together with the list of detected protein-ligand HBs and their respective frequency.

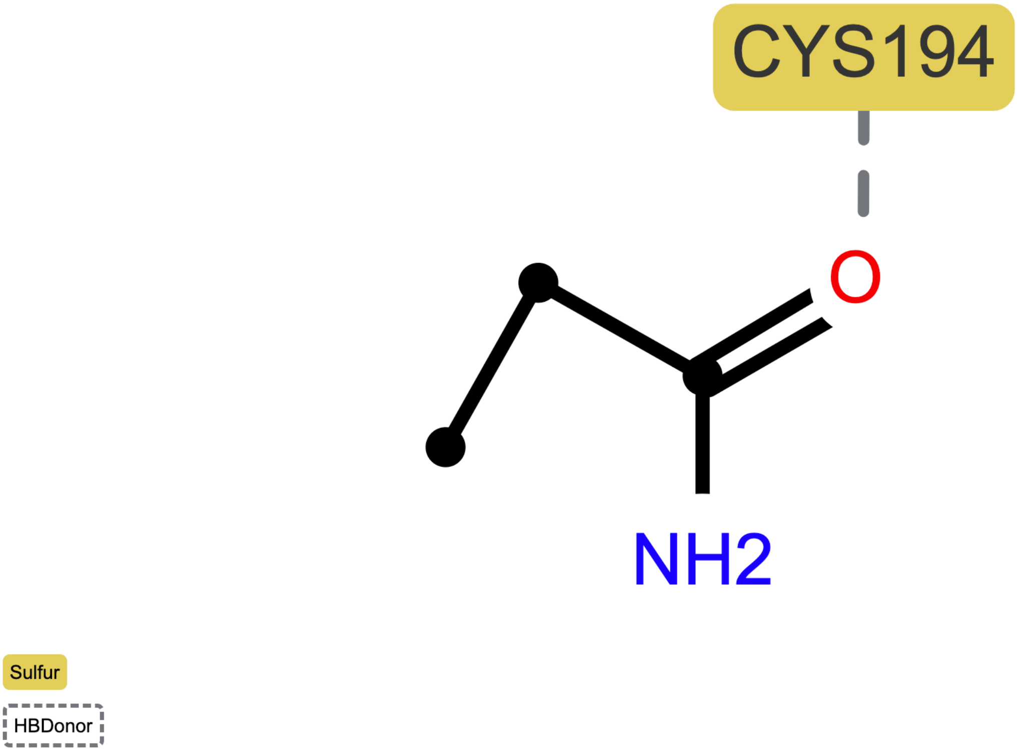

Immunoglobin Kappa Light Chain (C134) - Cluster 4			
residue	number	interaction	occurrence
Cys	194	HB donor	0.54

Figure S46: Modeled covalent adduct between acrylamide (ACR) and the target protein. (*Top*) Schematic representation of acrylamide and the hydrogen bonds (HBs) formed with the surrounding binding site residues. Residues are colored according to their physicochemical properties, as shown in the figure legend, and the corresponding HB frequency is represented through the width of the dashed line. (*Bottom*) Table indicating the target protein, reactive Cys and docking cluster considered, together with the list of detected protein-ligand HBs and their respective frequency.

1.4.6 Kinesins KIFC1 and KIF2C

Kinesins are motor proteins essential to transport cellular cargos along microtubules, powered by ATP hydrolysis. ACR-mediated inhibition of kinesins has been proposed as the molecular mechanism by which acrylamide impairs fast anterograde and retrograde axonal transport (Erkekoglu and Baydar, 2014). Indeed, experimental studies have confirmed that acrylamide is able to inhibit two kinesin motor proteins (Sickles et al., 1996, 2007; Friedman et al., 2008), namely KIFC5A and KRP2 (later renamed as KIFC1/HSET and KIF2C) (Miki et al., 2001). In order to pinpoint candidate reactive Cys, we first generated a multiple sequence alignment of kinesin sequences homologous to KIFC1/HSET and KIF2C, including human and rat isoforms belonging to kinesin families 14 and 13, respectively (Figure S47). Noteworthily, the sensitivity to acrylamide is much higher for KIFC5A/KIF2C than for KRP2/KIFC1/HSET (Friedman et al., 2008). Therefore, in this case we focused on cysteine residues that are conserved in one kinesin family but not the other.

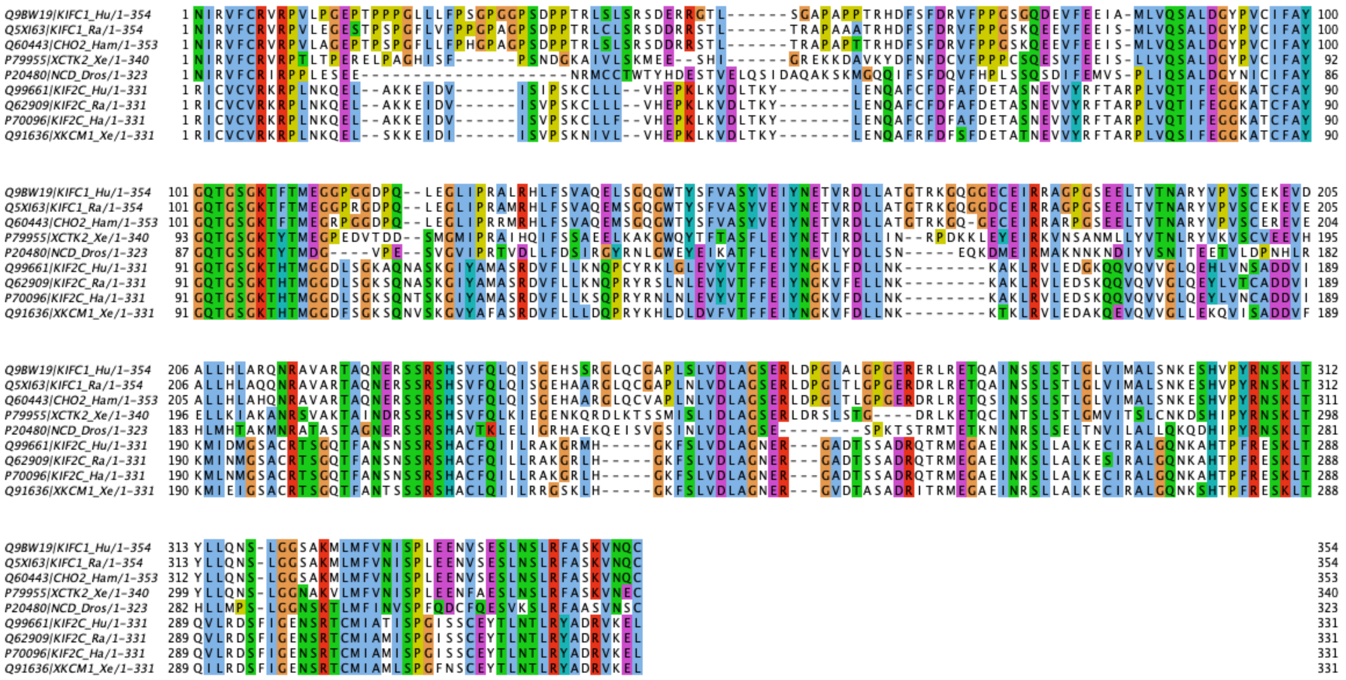

Figure S47: Multiple sequence alignment rat and human isoforms homologous to the kinesins KIFC1/HSET and KIF2C studied here, belongingto the kinesin 14 and 13 families, respectively. The MSA was generated using the MAFFT webserver (Katoh et al., 2022) and the same color code as in Figure S29 was used.

For KIF2C, we focused on the kinesin ATPase motor domain, as ACR-mediated inhibition of such catalytic domain has been observed for other neuronal proteins, such as NSF or v-ATPase (see main text). Among the six Cys conserved within the kinesin 13 family, integration of structural data revealed that C260 and C287 are more likely to play a functional role. In particular, a study performed for MACK (Talapatra et al., 2015), a hamster homolog of KIF2C, showed that ATPase activity requires dimerization of the motor domain and C260 and C287 are located at this dimeric interface. Moreover, dimerization is promoted by interaction with the C-terminal (CT) domain and C287 is located in the vicinity of CT (Talapatra et al., 2015). Indeed, introduction of a Cys mutation in the CT domain resulted in formation of a disulfide bond with C287 (Talapatra et al., 2015). Therefore, C260 and C287 were selected for covalent docking to KIF2C. Favorable docking scores were obtained for both C260 and C287

(-35.3 and -34.1 a.u., respectively). We surmise that attachment of ACR to either C260 or C287 in KIF2C could interfere with motor domain dimerization and/or CT domain binding.

For KIFC1/HSET, we again relied on experimental data to identify the best candidate residues among the conserved cysteines within the kinesin 14 family. In particular, a previous study identified C663 as an attachment site for covalent inhibitors (Förster et al., 2019). Therefore, we selected C663 for the covalent docking of acrylamide to KIFC1, resulting in a favorable docking score of -14.0 a.u.. We speculate that binding of acrylamide to C663 could impair ATPase activity of KIFC1/HSET based on its structural position. This cysteine is at the C-terminal end of KIFC1 and is part of the so-called $\alpha6$ helix, which together with $\alpha4$ forms a cleft known to bind inhibitors (Park et al., 2017). Moreover, the $\alpha4$-$\alpha46$ cleft is located adjacent to the P-loop (or walker A motif) responsible for ATP binding and hydrolysis. Thus, ACR modification of C663 could allosterically trigger rearrangements of the P-loop through either helix $\alpha4$ or $\alpha6$.

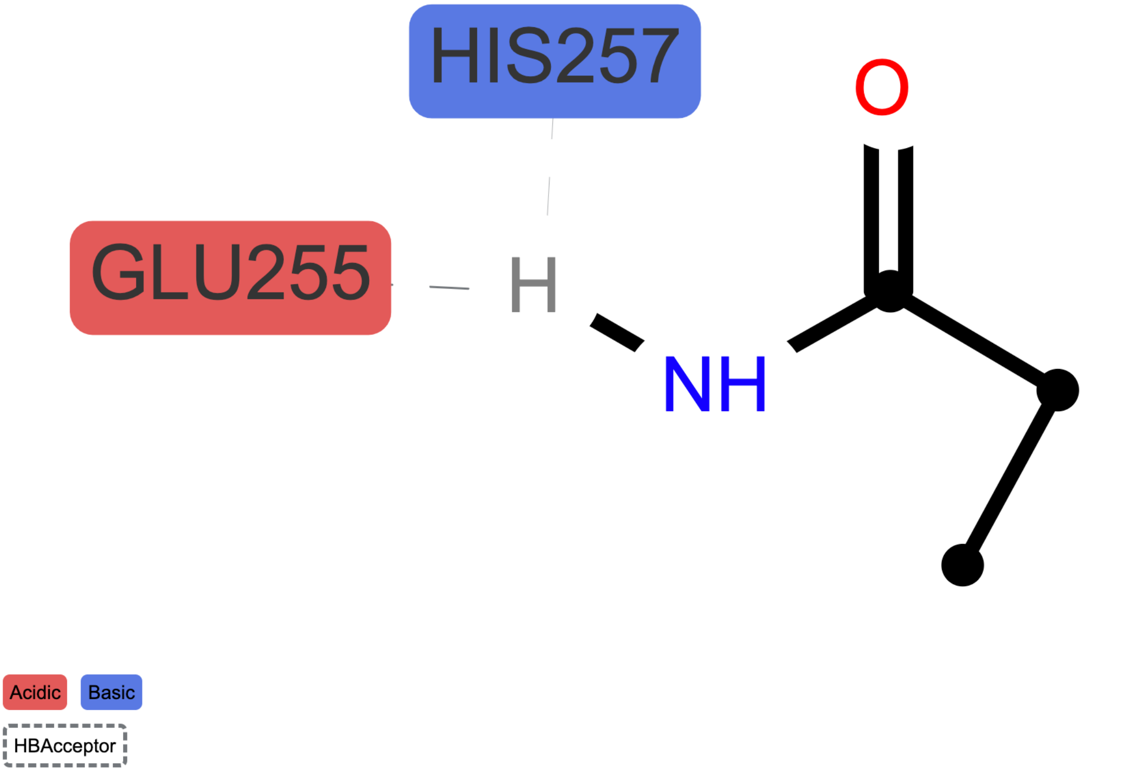

Kinesin KIF2C (C260) - Cluster 1			
residue	number	interaction	occurrence
Glu	255	HB acceptor	0.09
His	257	HB acceptor	0.01

Figure S48: Modeled covalent adduct between acrylamide (ACR) and the target protein. (*Top*) Schematic representation of acrylamide and the hydrogen bonds (HBs) formed with the surrounding binding site residues. Residues are colored according to their physicochemical properties, as shown in the figure legend, and the corresponding HB frequency is represented through the width of the dashed line. (*Bottom*) Table indicating the target protein, reactive Cys and docking cluster considered, together with the list of detected protein-ligand HBs and their respective frequency.

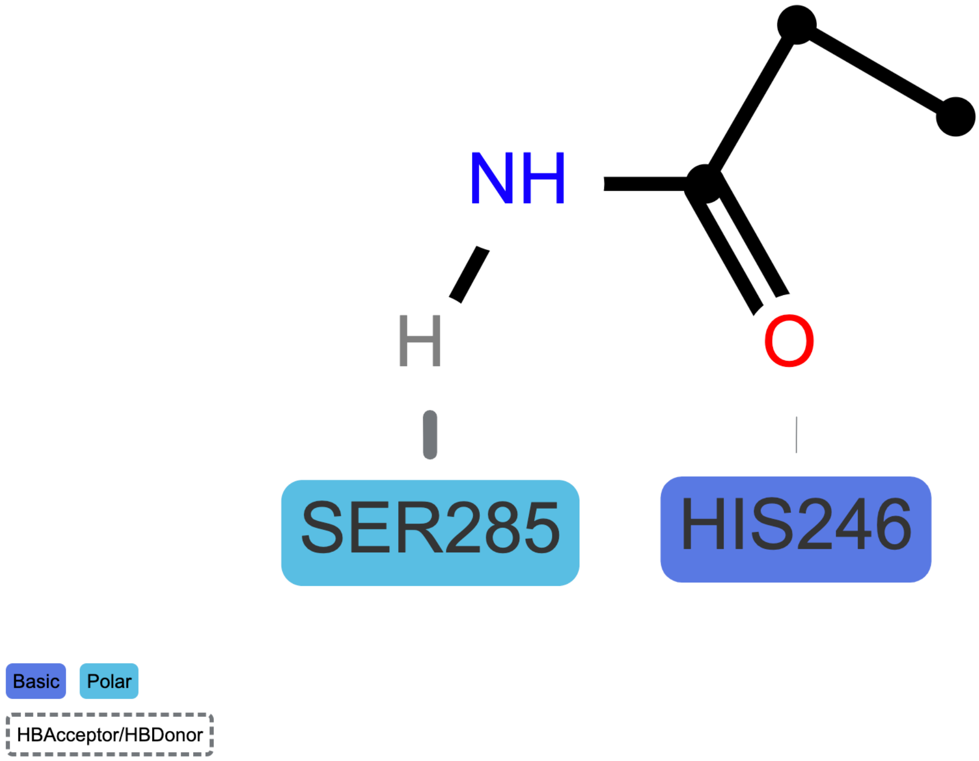

Kinesin KIF2C (C287) - Cluster 1			
residue	number	interaction	occurrence
His	246	HB donor	0.05
Ser	285	HB acceptor	0.66

Figure S49: Modeled covalent adduct between acrylamide (ACR) and the target protein. (*Top*) Schematic representation of acrylamide and the hydrogen bonds (HBs) formed with the surrounding binding site residues. Residues are colored according to their physicochemical properties, as shown in the figure legend, and the corresponding HB frequency is represented through the width of the dashed line. (*Bottom*) Table indicating the target protein, reactive Cys and docking cluster considered, together with the list of detected protein-ligand HBs and their respective frequency.

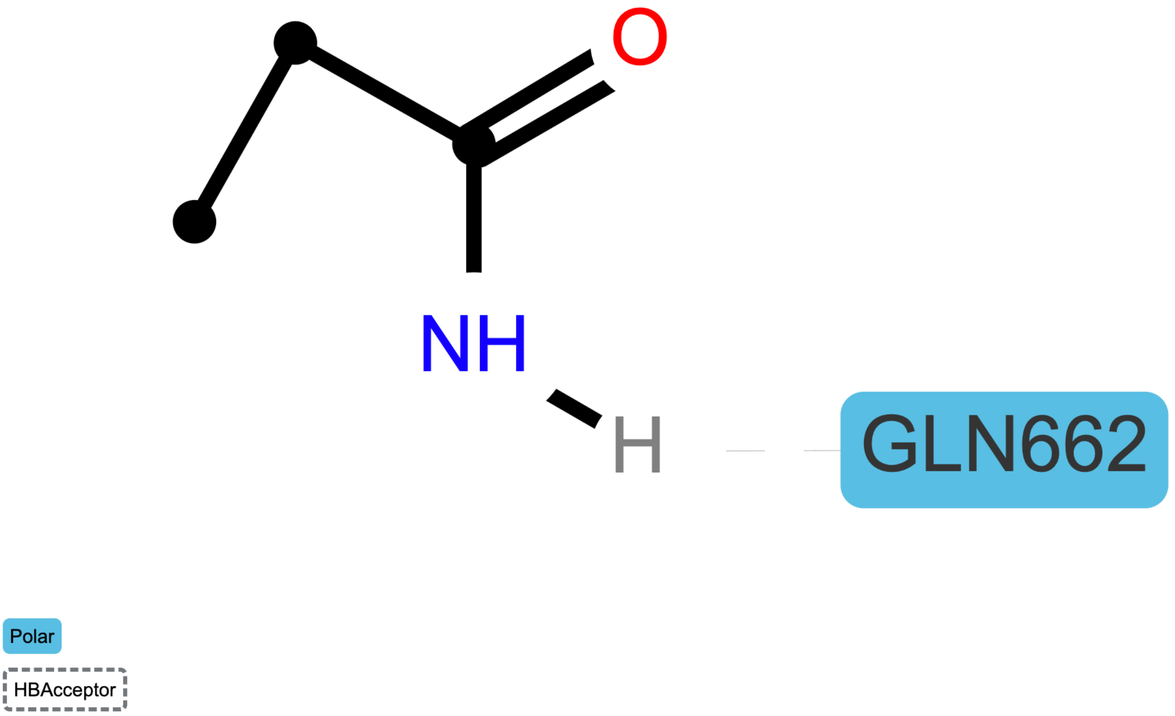

Kinesin KIF1C (C663) - Cluster 1			
residue	number	interaction	occurrence
Gln	662	HB acceptor	0.01

Figure S50: Modeled covalent adduct between acrylamide (ACR) and the target protein. (*Top*) Schematic representation of acrylamide and the hydrogen bonds (HBs) formed with the surrounding binding site residues. Residues are colored according to their physicochemical properties, as shown in the figure legend, and the corresponding HB frequency is represented through the width of the dashed line. (*Bottom*) Table indicating the target protein, reactive Cys and docking cluster considered, together with the list of detected protein-ligand HBs and their respective frequency.

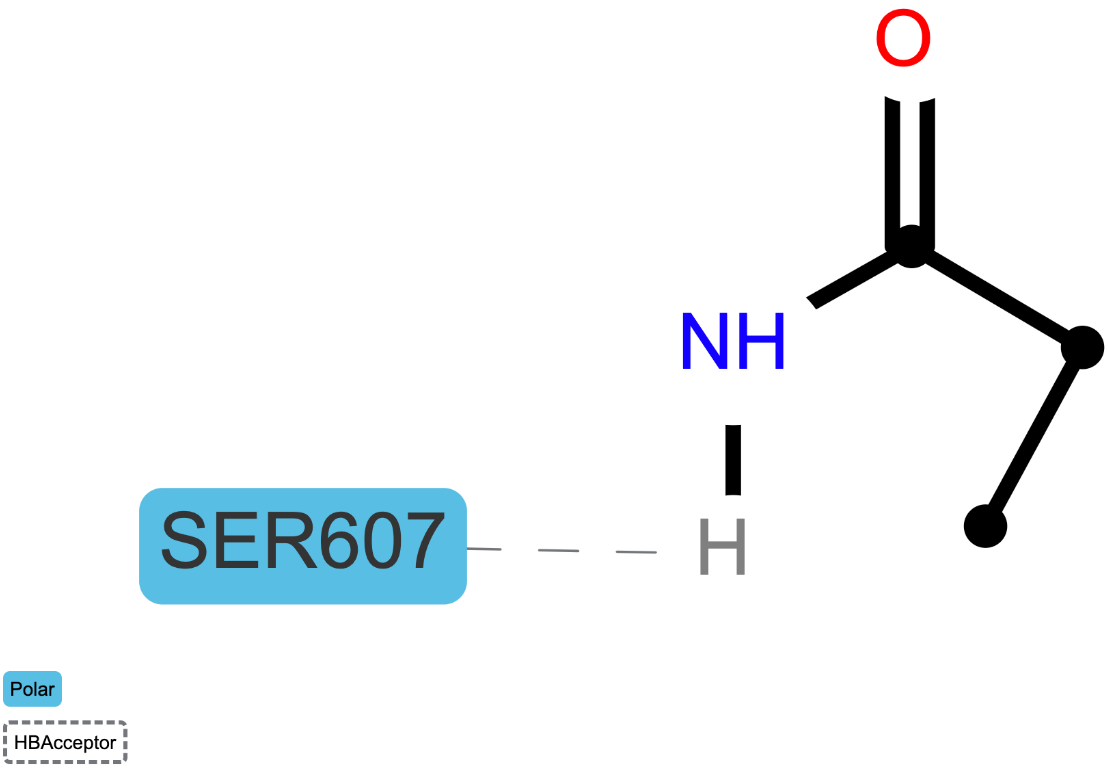

Kinesin KIF1C (C663) - Cluster 3			
residue	number	interaction	occurrence
Ser	607	HB acceptor	0.11

Figure S51: Modeled covalent adduct between acrylamide (ACR) and the target protein. (*Top*) Schematic representation of acrylamide and the hydrogen bonds (HBs) formed with the surrounding binding site residues. Residues are colored according to their physicochemical properties, as shown in the figure legend, and the corresponding HB frequency is represented through the width of the dashed line. (*Bottom*) Table indicating the target protein, reactive Cys and docking cluster considered, together with the list of detected protein-ligand HBs and their respective frequency.

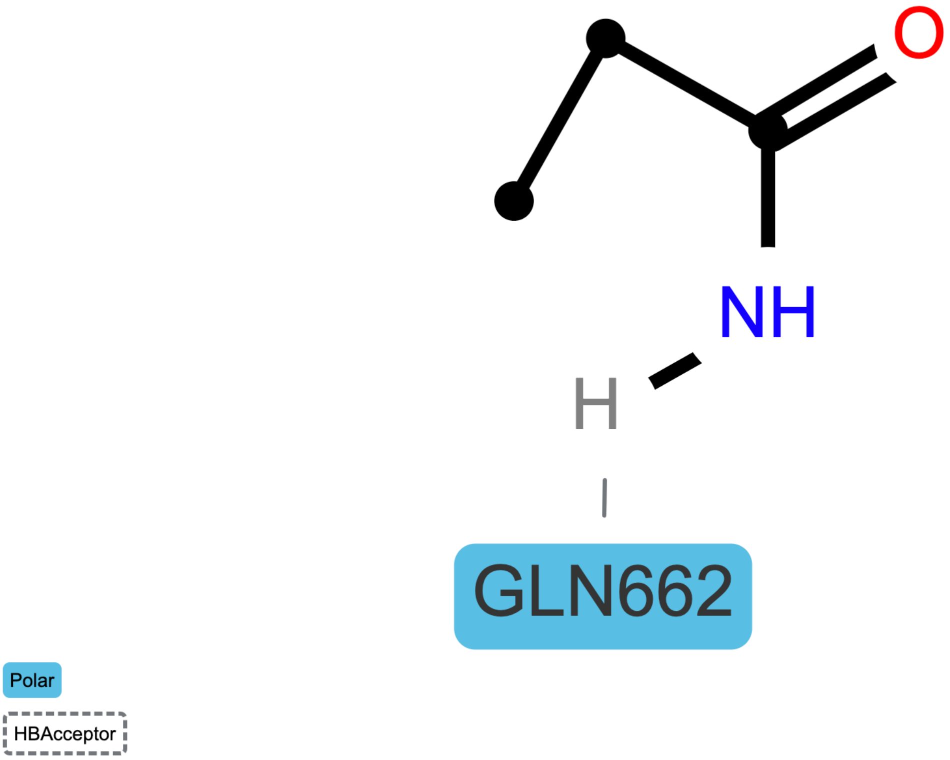

Kinesin KIF1C (C663) - Cluster 6			
residue	number	interaction	occurrence
Gln	662	HB acceptor	0.20

Figure S52: Modeled covalent adduct between acrylamide (ACR) and the target protein. (*Top*) Schematic representation of acrylamide and the hydrogen bonds (HBs) formed with the surrounding binding site residues. Residues are colored according to their physicochemical properties, as shown in the figure legend, and the corresponding HB frequency is represented through the width of the dashed line. (*Bottom*) Table indicating the target protein, reactive Cys and docking cluster considered, together with the list of detected protein-ligand HBs and their respective frequency.

1.4.7 Sex Hormone-Binding Globulin (SHBG)

Sex hormone-binding globulin is a glycoprotein that binds steroid hormones to regulate the amount of free steroid molecules in plasma. SHBG contains cysteine-rich regions essential for ligand binding. Indeed, truncated mutants of rabbit SHBG, which did not contain the disulfide bond formed between C164 and C188, were not able to bind androgens anymore (Wong et al., 2001). Moreover, these two cysteines are highly conserved among G-domains containing proteins, such as rabbit SHBG, and have been shown to contribute to protein stability (Wong et al., 2001). Therefore, we selected C164 and C188 as potential ACR binding sites to perform the subsequent covalent docking calculations. Nonetheless, we would like to note that these two cysteines are involved in a disulfide bridge and thus prior reduction or chemical modification of the disulfide bridge would be needed to react with acrylamide, as discussed in section 1.4.5.

The docking results show favorable docking scores for both candidate cysteines, $-23.0\,\mathrm{a.u.}$ for C164 and $-16.6\,\mathrm{a.u.}$ for C188, as well as stabilizing protein-ligand interactions (see figures below). Considering the aforementioned changes in ligand binding and protein stability upon mutation or truncation of these cysteines, it is tempting to speculate that ACR modification of C164 and C188 will have a similar effect.

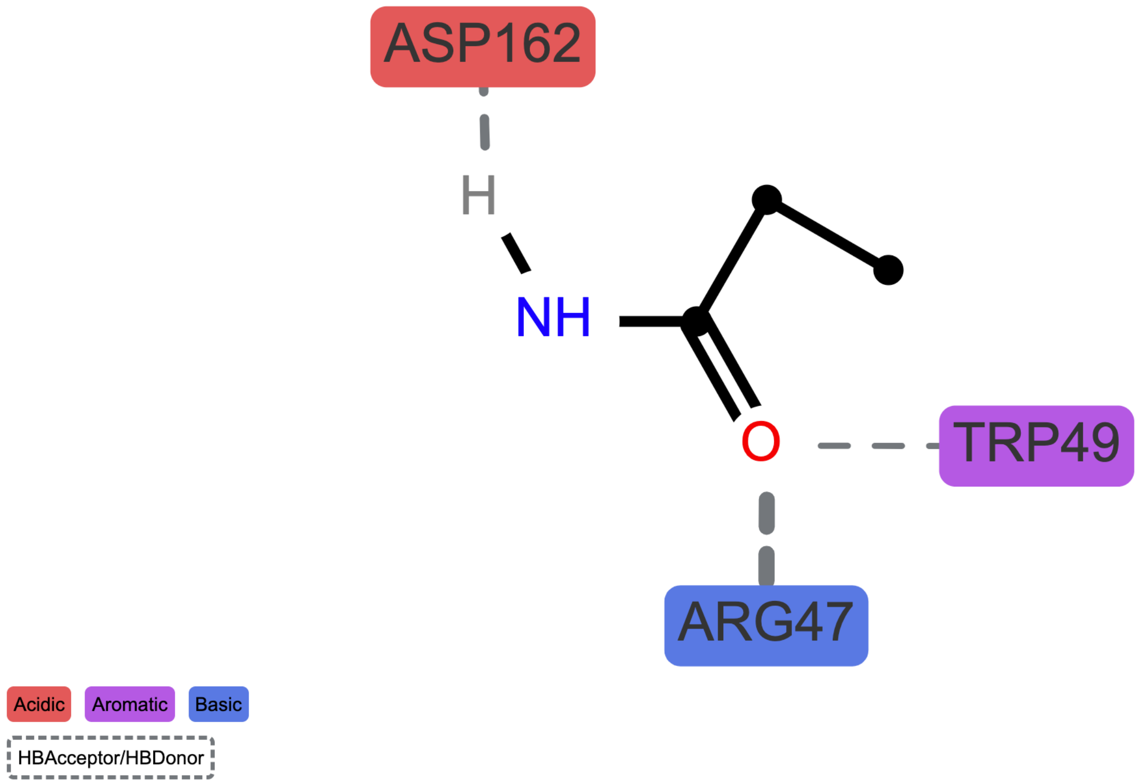

Sex Hormone Binding Globulin (C164) - Cluster 1			
residue	number	interaction	occurrence
Arg	47	HB donor	1.00
Trp	49	HB donor	0.38
Asp	162	HB acceptor	0.59

Figure S53: Modeled covalent adduct between acrylamide (ACR) and the target protein. (*Top*) Schematic representation of acrylamide and the hydrogen bonds (HBs) formed with the surrounding binding site residues. Residues are colored according to their physicochemical properties, as shown in the figure legend, and the corresponding HB frequency is represented through the width of the dashed line. (*Bottom*) Table indicating the target protein, reactive Cys and docking cluster considered, together with the list of detected protein-ligand HBs and their respective frequency.

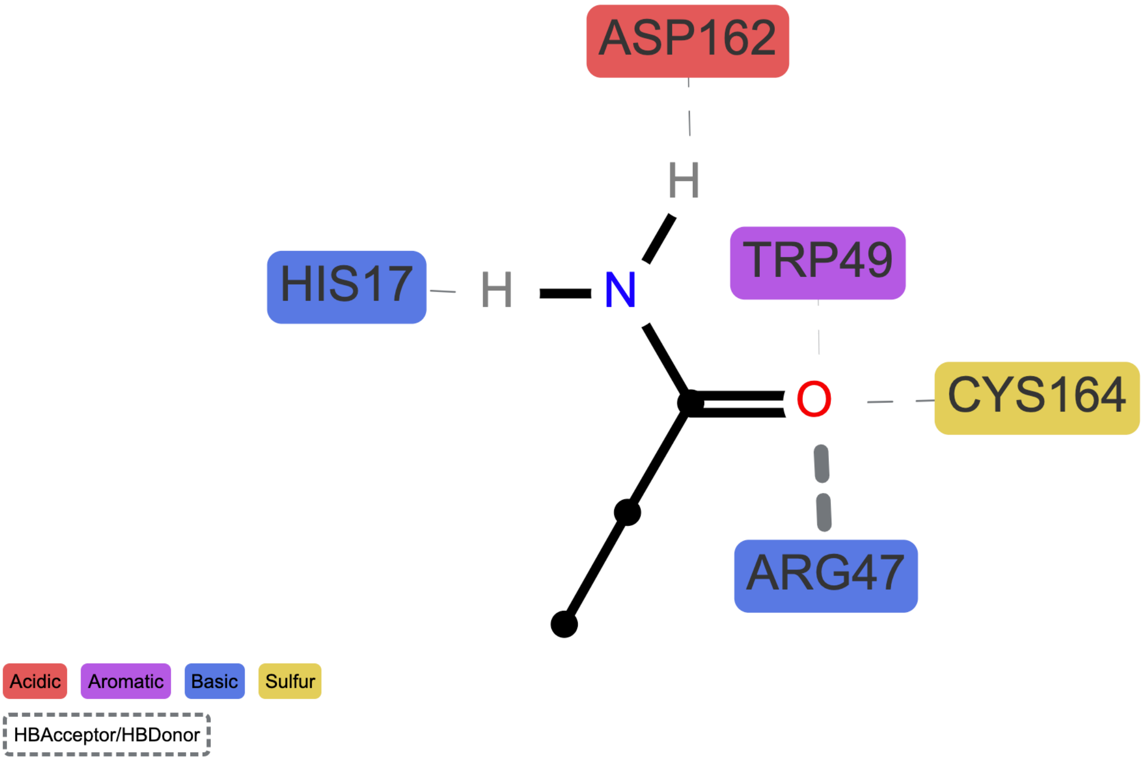

Sex Hormone Binding Globulin (C188) - Cluster 1			
residue	number	interaction	occurrence
His	17	HB acceptor	0.11
Arg	47	HB donor	1.00
Trp	49	HB donor	0.03
Asp	162	HB acceptor	0.09
Cys	164	HB donor	0.13

Figure S54: Modeled covalent adduct between acrylamide (ACR) and the target protein. (*Top*) Schematic representation of acrylamide and the hydrogen bonds (HBs) formed with the surrounding binding site residues. Residues are colored according to their physicochemical properties, as shown in the figure legend, and the corresponding HB frequency is represented through the width of the dashed line. (*Bottom*) Table indicating the target protein, reactive Cys and docking cluster considered, together with the list of detected protein-ligand HBs and their respective frequency.

1.4.8 Topoisomerase IIa

Topoisomerases are highly conserved enzymes involved in multiple processes taking place at the cell nucleus, such DNA replication, chromosome condensation and chromosome segregation. Acrylamide has been shown to inhibit topoisomerase II activity in nuclear extracts (Sciandrello et al., 2010). However, the molecular mechanism of such inhibition is unclear, as acrylamide does not induce DNA cleavage, unlike other sulfhydryl-reactive agents modifying topoisomerase II. Therefore, we inspected the whole protein structure to pinpoint possible Cys sites targeted by ACR and selected two candidates, C170 and C997. C170 is located near the active site of the ATPase domain and thus its modification by ACR could hinder ATP binding, as shown for other ATPases in this study. Instead, C997 belongs to the Toprim (topoisomerase-primase) domain, the catalytic domain involved in DNA strand cleavage and religation. The location of C997 at the protein-DNA interface suggests that its modification by ACR could interfere with Toprim catalytic activity.

Covalent docking to C170 showed that this cysteine is too buried inside the protein to allow formation of the adduct. In contrast, docking to C997 showed a favorable docking score of $-8.4\,\mathrm{a.u.}$ for the top (best scored) cluster, thus supporting this cysteine as the primary site of ACR modification. This is in line with acrylamide being described as a catalytic inhibitor that reduces the amount of catalytically competent enzyme sensitive to the topoisomerase II poison etoposide (Sciandrello et al., 2010).

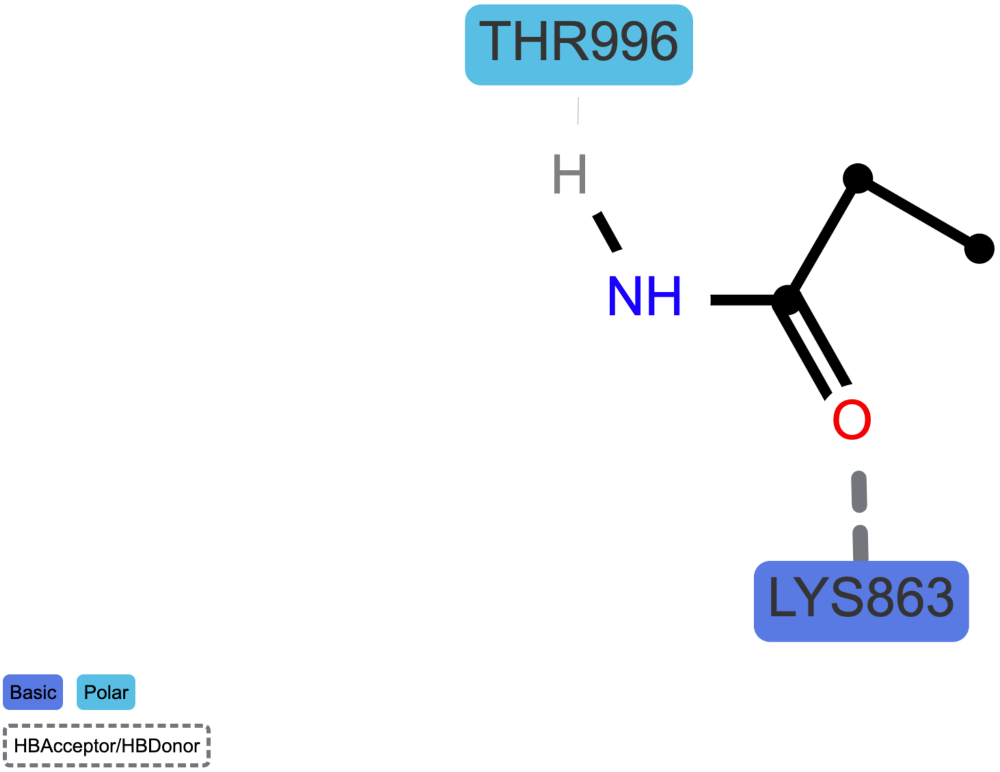

Topoisomerase II (C997) - Cluster 1			
residue	number	interaction	occurrence
Lys	863	HB donor	0.95
Thr	996	HB acceptor	0.03

Figure S55: Modeled covalent adduct between acrylamide (ACR) and the target protein. (*Top*) Schematic representation of acrylamide and the hydrogen bonds (HBs) formed with the surrounding binding site residues. Residues are colored according to their physicochemical properties, as shown in the figure legend, and the corresponding HB frequency is represented through the width of the dashed line. (*Bottom*) Table indicating the target protein, reactive Cys and docking cluster considered, together with the list of detected protein-ligand HBs and their respective frequency.

1.5 Analysis of hydrophobic interactions

Besides the H-bonds described in the main text (see section 3.4), we also analyzed the hydrophobic interactions between the two methylene carbon atoms of the ACR adduct and nearby protein residues, as explained in the Methods (section 2.4 in the main text). The results are presented in Table S5. No clear preference for aliphatic/aromatic or small/branched amino acids was identified. We speculate that this is due to the small size and flexibility of the ACR adduct, which allow the ligand to form diverse (and non-directional) hydrophobic interactions.

Table S5: Hydrophobic interactions

Protein name	Cysteine	Residue	Number	Atom	Occurence
Albumin	34	Val	77	CG2	7.58
		Val	77	CG1	92.42
		Leu	31	CD1	64.39
		Leu	31	CD2	3.03
		Leu	41	CD1	39.39
		Asp	38	CB	57.58
Alcohol Dehydrogenase	170	Leu	166	CB	100.0
		Val	41	CG1	25.64
		Val	41	CB	69.23
		Ala	42	CB	100.00
		Ala	70	CB	46.15
Alcohol Dehydrogenase	240	Lys	233	CG	12.50
		Phe	229	CB	61.11
		Phe	229	CD2	37.50
Aldolase	134	Ile	176	CG2	97.32
		Ala	135	CB	97.32
		Gln	179	CB	64.29
		Asn	180	CB	100.00
	239	Asp	197	CB	64.91
		Lys	241	CB	9.36
		Lys	241	CG	7.60
		Lys	241	CD	76.60
	268	Ile	29	CD1	94.20
		Leu	227	CD2	3.62
		Leu	227	CD1	15.22
		Leu	227	CB	81.16
		Val	23	CG1	91.30
		Val	23	CG2	1.45
	289	Glu	246	CB	18.84
		Ala	249	CB	97.10
		Ala	285	CB	11.59
		Met	250	CB	37.68
Creatine Kinase	141	His	145	CD2	96.83
		His	145	CG	1.59
	283	Val	75	CG1	60.66
		Val	72	CG1	77.05
		Val	72	CG2	9.84
		Leu	201	CD2	3.28

Protein name	Cysteine	Residue	Number	Atom	Occurence
Dopamine Receptor	114	Phe	418	CZ	100.0
		Phe	417	CE2	85.44
		Val	120	CG1	1.94
		Trp	414	CZ2	0.97
		Trp	414	CZ3	98.06
Dopamine Transporter (inward)	342	Tyr	115	CE2	12.08
		Tyr	115	CE1	55.71
		Ala	119	CB	58.39
		Gln	122	CB	12.08
		Gln	122	CG	23.49
		Leu	118	CB	59.73
Dopamine Transporter (outward)	342	Ala	119	CB	1.19
		Gln	122	CB	2.38
		Met	511	CB	96.43
		Ile	508	CB	3.57
		Thr	512	CG	38.10
Enolase	388	Ile	143	CG1	0.83
		Leu	431	CD2	50.41
		Asn	139	CB	90.91
		Glu	141	CB	57.85
	398	Arg	514	CG	2.40
		Arg	514	CB	20.96
		Phe	212	CE2	2.99
		Val	206	CG1	71.86
		Tyr	188	CE2	41.32
Estrogen Receptor	381	His	247	CD2	7.41
	530	Tyr	226	CE2	31.68
		Tyr	226	CE1	43.56
		Lys	229	CD	2.97
		Lys	229	CB	0.99
Glyceraldehyde-3-phosphate dehydrogenase	152	Tyr	320	CD2	34.97
		Tyr	320	CG	0.61
		Tyr	320	CE2	5.52
		Asn	316	CB	13.50
		Ile	14	CD1	4.29
Hemoglobin	93	Thr	41	CG2	12.08
		Tyr	145	CD2	76.58
		Asp	94	CB	0.63
		His	146	CD2	30.38
		Lys	40	CB	0.63
Immunoglobulin G1 H Nie	395	Pro	378	CB	64.62
		Lys	464	CG	92.31
		Lys	464	CB	1.54
		Val	466	CG2	100.00
		Val	376	CG1	18.46

Protein name	Cysteine	Residue	Number	Atom	Occurence
Immunoglobin kappa light chain	134	Lys	207	CB	28.68
		Ile	117	CB	63.24
		Ile	117	CD1	19.12
		Leu	136	CD1	2.21
		Val	115	CG1	44.12
		Val	115	CB	41.91
		Val	196	CG2	4.41
Kinesin KIFC1	663	Met	317	CB	0.93
		Lys	372	CB	2.78
		Ile	316	CG2	4.63
Kinesin KIF2C	260	Glu	49	CB	11.24
		Glu	49	CG	1.12
		Phe	107	CD2	6.74
		Phe	107	CE2	89.89
		Asp	106	CB	97.75
		His	51	CB	88.76
		His	51	CD2	5.62
	287	Lys	80	CB	1.681
		Lys	80	CG	53.782
		Leu	82	CD1	2.521
NEM-sensitive factor	264	Lys	266	CB	0.86
		Ala	439	CB	2.56
Sex Hormone-Binding Globulin	164	His	17	CG	3.01
		His	17	CD2	46.62
	188	His	17	CD2	10.00
Topoisomerase IIa (Toprim domain)	997	Tyr	907	CE2	0.971
		Leu	995	CB	8.74
		Leu	995	CD1	87.38
		Pro	865	CG	14.56
		Lys	863	CG	99.03
		Lys	863	CB	0.97
Vesicular proton ATPase	254	Ala	251	CB	99.09
		Lys	437	CB	70.91
		Lys	437	CG	1.82
		Pro	249	CB	0.91

1.6 Dependence of the covalent docking results on the input structure

As mentioned in the main text (see section 4), the docking approach is fully flexible, so that the protein structure can adapt to the ACR covalent adduct. Nonetheless, we checked whether the results were robust with respect to the initial protein structure. As a test case, we chose GAPDH because (i) there are several experimental structures available and (ii) it contains three Cys residues that have been shown experimentally to be modified by ACR (C152, C156 and C247). Besides the protein structure reported in the main text (PDB code 4WNC), we tested two more (PDB codes 1U8F and 6YND), solved at different resolution (see Table S6). Regardless of the input structure, the primary ACR binding site C152 still exhibits a better docking score than the two Cys residues modified only at high ACR concentrations (C156 and 247). Therefore, this test case further supports that covalent docking can be used to pinpoint the most reactive Cys site within a given protein.

Table S6. Covalent docking results for a subset of GAPDH crystal structures. For each considered Cys, the Haddock score of the top docking cluster is shown.

Protein name	PDB code	Resolution (Å)	C152	C156	C247
Glyceraldehyde-3-phosphate dehydrogenase	4WNC	1.99	-12.3	46.1	9.9
	1U8F	1.75	-16.8	-3.1	0.3
	6YND	1.52	-9.5	8.2	7.8

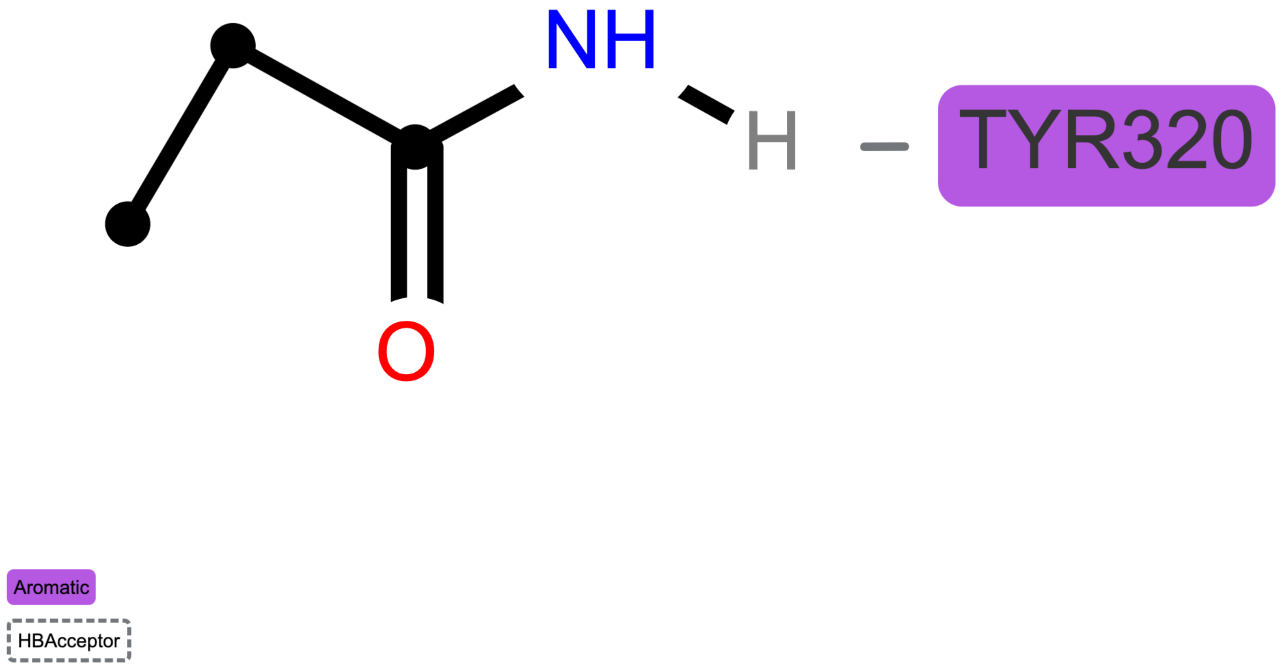

Glyceraldehyde-3-Phosphate Dehydrogenase (C152) - PDB 1U8F - Cluster 1			
residue	number	interaction	occurrence
Tyr	320	HB acceptor	0.39

Figure S56: Modeled covalent adduct between acrylamide (ACR) and the target protein. (*Top*) Schematic representation of acrylamide and the hydrogen bonds (HBs) formed with the surrounding binding site residues. Residues are colored according to their physicochemical properties, as shown in the figure legend, and the corresponding HB frequency is represented through the width of the dashed line. (*Bottom*) Table indicating the target protein, reactive Cys and docking cluster considered, together with the list of detected protein-ligand HBs and their respective frequency.

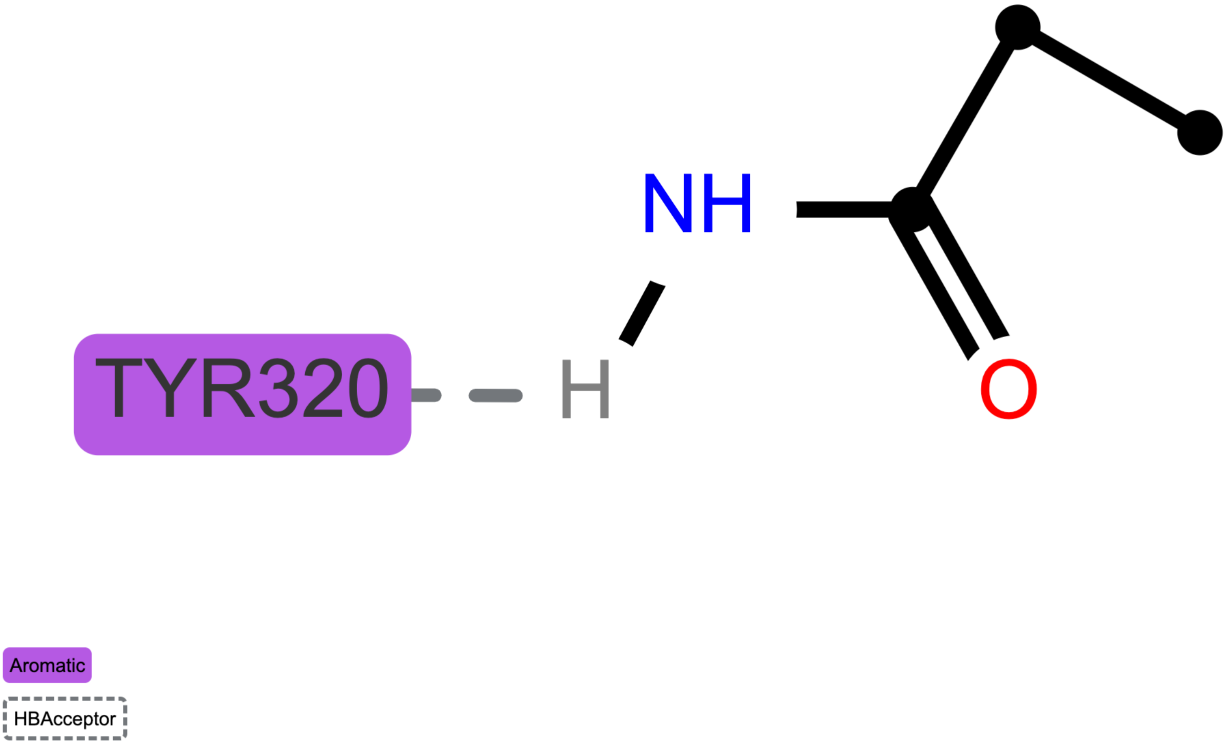

Glyceraldehyde-3-Phosphate Dehydrogenase (C152) - PDB 6YND - Cluster 1			
residue	number	interaction	occurrence
Tyr	320	HB acceptor	0.60

Figure S57: Modeled covalent adduct between acrylamide (ACR) and the target protein. (*Top*) Schematic representation of acrylamide and the hydrogen bonds (HBs) formed with the surrounding binding site residues. Residues are colored according to their physicochemical properties, as shown in the figure legend, and the corresponding HB frequency is represented through the width of the dashed line. (*Bottom*) Table indicating the target protein, reactive Cys and docking cluster considered, together with the list of detected protein-ligand HBs and their respective frequency.

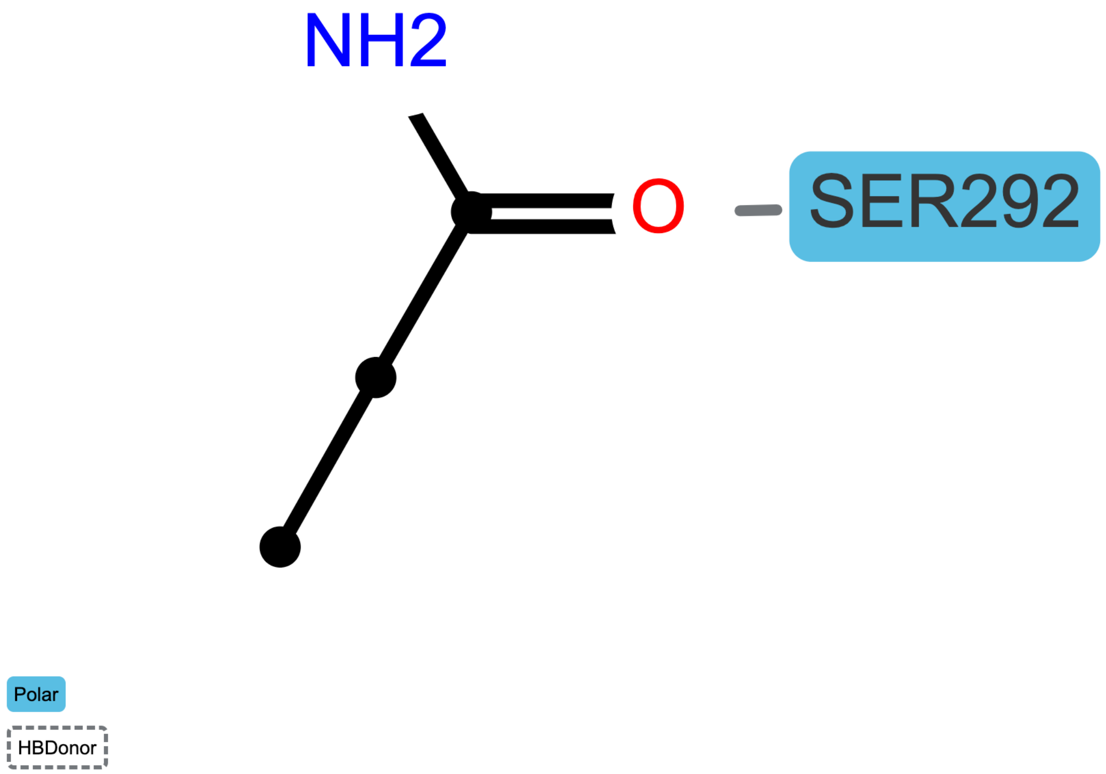

Glyceraldehyde-3-Phosphate Dehydrogenase (C156) - PDB 6YND - Cluster 1			
residue	number	interaction	occurrence
Ser	292	HB acceptor	0.50

Figure S58: Modeled covalent adduct between acrylamide (ACR) and the target protein. (*Top*) Schematic representation of acrylamide and the hydrogen bonds (HBs) formed with the surrounding binding site residues. Residues are colored according to their physicochemical properties, as shown in the figure legend, and the corresponding HB frequency is represented through the width of the dashed line. (*Bottom*) Table indicating the target protein, reactive Cys and docking cluster considered, together with the list of detected protein-ligand HBs and their respective frequency.

REFERENCES

Aliau, S., Mettras, H., Richard, E., and Borgna, J.-L. (1999). Cysteine 530 of the Human Estrogen Receptor α Is the Main Covalent Attachment Site of 11β-(Aziridinylalkoxyphenyl)estradiols. *Biochemistry* 38, 14752–14762

Awoonor-Williams, E. and Rowley, C. N. (2016). Evaluation of methods for the calculation of the pK_a of cysteine residues in proteins. *J. Chem. Theory Comput.* 12, 4662–4673

Baler, K., Martin, O. A., Carignano, M. A., Ameer, G. A., Vila, J. A., , et al. (2014). Electrostatic Unfolding and Interactions of Albumin Driven by pH Changes: A Molecular Dynamics Study. *J. Phys. Chem. B* 118, 921–930

Basile, A., Ferranti, P., Moccaldi, R., Spagnoli, G., and Sannolo, N. (2008). Proteomic approach for the analysis of acrylamide–hemoglobin adducts: Perspectives for biological monitoring. *J. Chromatogr. A* 1215, 74–81

Berfield, J. L., Wang, L. C., and Reith, M. E. (1999). Which form of dopamine is the substrate for the human dopamine transporter: the cationic or the uncharged species? *J. Biol. Chem.* 274, 4876–4882

Berman, H. M., Westbrook, J., Feng, Z., Gilliland, G., Bhat, T., Weissig, H., et al. (2000). The Protein Data Bank. *Nucleic Acids Res.* 28, 235–242

Bouysset, C. and Fiorucci, S. (2021). ProLIF: a library to encode molecular interactions as fingerprints. *J. Cheminform.* 13, 1–9

Burley, S. K., Bhikadiya, C., Bi, C., Bittrich, S., Chen, L., Crichlow, G. V., et al. (2021). RCSB Protein Data Bank: Powerful new tools for exploring 3D structures of biological macromolecules for basic and applied research and education in fundamental biology, biomedicine, biotechnology, bioengineering and energy sciences. *Nucleic Acids Res.* 49, D437–D451

de Oliveira, V. M., Dias, M. M., Thayná M Avelino, N. B. V., Fernando B da Silva, T. R. D., Whitford, P. C., Leite, V. B., et al. (2022). pH and the breast cancer recurrent mutation D538G affect the process of activation of estrogen receptor α. *Biochemistry* 65, 455–463

De Vries, S. J., Van Dijk, M., and Bonvin, A. M. (2010). The HADDOCK web server for data-driven biomolecular docking. *Nat. Protoc.* 5, 883–897

Dixit, R., Mukhtar, H., and Seth, P. K. (1981). *In vitro* inhibition of alcohol dehydrogenase by acrylamide: interaction with enzyme-SH groups. *Toxicol. Lett.* 7, 487–492

Dobryszycki, P., Rymarczuk, M., Bulaj, G., and Marian, K. (1999a). Effect of acrylamide on aldolase structure. I. Induction of intermediate states. *Biochem. Biophys. Acta* 1431, 338–350

Dobryszycki, P., Rymarczuk, M., Gapiński, J., and Marian, K. (1999b). Effect of acrylamide on aldolase structure. II. Characterization of aldolase unfolding intermediates. *Biochem. Biophys. Acta* 1431, 351–362

Eagles, P. A. M. and Iqbal, M. (1973). A Comparative Study of Aldolase from Human Muscle and Liver. *Biochem. J.* 113, 429–439

Eklund, H., Nordström, B., Zeppezauer, E., Söderlund, G., Ohlsson, I., Boiwe, T., et al. (1976). Three-dimensional structure of horse liver alcohol dehydrogenase at 2.4Å resolution. *J. Mol. Biol.* 120, 27–59

Erkekoglu, P. and Baydar, T. (2014). Acrylamide neurotoxicity. *Nutr. Neurosci.* 17, 49–57

Feng, C.-H. and Lu, C.-Y. (2011a). Modification of major plasma proteins by acrylamide and glycidamide: Preliminary screening by nano liquid chromatography with tandem mass spectrometry. *Anal. Chim. Acta* 687, 89–95

Feng, C.-H. and Lu, C.-Y. (2011b). Modification of major plasma proteins by acrylamide and glycidamide: Preliminary screening by nano liquid chromatography with tandem mass spectrometry. *Anal. Chim. Acta* 684, 89–95

Ferguson, S. A., Garey, J., Smith, M. E., Twaddle, N. C., and Doerge, M. G., Daniel R. Paule (2010). Preweaning behaviors, developmental landmarks, and acrylamide and glycidamide levels after pre- and postnatal acrylamide treatment in rats. *Neurotoxicol. Teratol.* 32, 373–381

Friedman, M. A., Zeiger, E., Marroni, D. E., and Sickles, D. W. (2008). Inhibition of rat testicular nuclear kinesins (krp2; KIFC5A) by acrylamide as a basis for establishing a genotoxicity threshold. *J. Agric. Food Chem.* 15, 6024–6030

Förster, T., Shang, E., Shimizu, K., Sanada, E., Schölermann, B., Huebecker, M., et al. (2019). 2-Sulfonylpyrimidines target the kinesin HSET via cysteine alkylation. *Eur. J. Org. Chem.* 2019, 5486–5496

Gardiner, L. P., Roper, D. I., Hammonds, T. R., and Maxwell, A. (1998). The N-terminal domain of human topoisomerase IIα is a DNA-dependent ATPase. *Biochemistry* 37, 16997–17004

Groftehauge, M., Truan, D., Vasil, A., Denny, P., Vasil, M., and Pohl, E. (2015). Crystal structure of a hidden protein, YcaC, a putative cysteine hydrolase from *Pseudomonas aeruginosa*, with and without an acrylamide adduct. *Int. J. Mol. Sci.* 16, 15971–15984

HADDOCK developer team (2018). HADDOCK covalent docking tutorial https://www.bonvinlab.org/education/biomolecular-simulations-2018/HADDOCK_tutorial/ [Accessed: December 6th, 2022]

Hansch, C., Klein, T., McClarin, J., Langridge, R., and Cornell, N. W. (1986). A quantitative structure-activity relationship and molecular graphics analysis of hydrophobic effects in the interactions of inhibitors with alcohol dehydrogenase. *J. Med. Chem.* 29, 615–620

Heinz, F. and Freimüller, B. (1982). Glyceraldehyde-3-phosphate Dehydrogenase from Human Tissues. *Methods Enzymol.* 89, 301–305

Hogervorst, J. G. F., Baars, B.-J., Schouten, L. J., Konings, E. J. M., Goldbohm, R. A., and van den Brandt, P. A. (2009). The carcinogenicity of dietary acrylamide intake: A comparative discussion of epidemiological and experimental animal research. *Crit. Rev. Toxicol.* 40, 485–512

Howland, R. D., Vyas, I. L., Lowndes, H. E., and Argentieri, T. M. (1980). The etiology of toxic peripheral neuropathies: *In vitro* effects of acrylamide and 2,5-hexanedione on brain enolase and other glycolytic enzymes. *Brain Res.* 202, 131–142

Imai, K. and Yonetani, T. (1975). pH dependence of the Adair constants of human hemoglobin. Nonuniform contribution of successive oxygen bindings to the alkaline Bohr effect. *J. Biol. Chem.* 250, 2227–2231

Ishii, T. and Uchida, K. (2004). Induction of reversible cysteine-targeted protein oxidation by an endogenous electrophile 15-deoxy-$\triangle^{12,14}$-prostaglandin j2. *Chem. Res. Toxicol.* 17, 1313–1322

Kapolka, N. J., Rowe, J. B., Taghon, G. J., and Isom, D. G. (2021). Proton-gated coincidence detection is a common feature of GPCR signaling. *Proc. Natl. Acad. Sci. USA* 118, e2100171118

Katoh, K., Misawa, K., Kei-ichi, K., and Miyata, T. (2022). MAFFT: a novel method for rapid multiple sequence alignment based on fast Fourier transform. *Nucleic Acids Res.* 30, 3059–3066

Lin, L., Perryman, B., Friedman, D., Roberts, R., and Ma, T. S. (1994). Determination of the catalytic site of creatine kinase by site-directed mutagenesis. *Biochem. Biophys. Acta* 1206, 97–104

Liu, H. and May, K. (2012). Disulfide bond structures of IgG molecules: structural variations, chemical modifications and possible impacts to stability and biological function. *mAbs* 4, 17–23

LoPachin, R. M. and Barber, D. S. (2006). Synaptic cysteine sulfhydryl groups as targets of electrophilic neurotoxicants. *Toxicol. Sci.* 94, 240–255

Lutolf, M., Tirelli, N., Cerritelli, S., Cavalli, L., and Hubbell, J. (2001). Systematic modulation of Michael-type reactivity of thiols through the use of charged amino acids. *Bioconj. Chem.* 12, 1051–1056

Martyniuk, C. J., Fang, B., Koomen, J. M., Gavin, T., Zhang, L., Barber, D. S., et al. (2011). Molecular mechanism of glyceraldehyde-3-phosphate dehydrogenase inactivation by α,β-unsaturated carbonyl derivatives. *Chem. Res. Toxicol.* 24, 2301–2311

Matsuoka, M., Matsumura, H., and Igisu, H. (1996). Creatine kinase activities in brain and blood: possible neurotoxic indicator of acrylamide intoxication. *J. Occup. Environ. Med.* 53, 468–471

Meng, F.-G., Zhou, H.-W., and Zhou, H.-M. (2001). Effects of acrylamide on creatine kinase from rabbit muscle. *Int. J. Biochem. Cell Biol.* 33, 1064–1070

Miki, H., Setou, M., Kenshiro, K., and Hirokawa, N. (2001). All kinesin superfamily protein, KIF, genes in mouse and human. *Proc. Natl. Acad. Sci. USA* 98, 7004–7011

Nair, D. P., Podgorski, M., Chatani, S., Gong, T., Xi, W., Fenoli, C. R., et al. (2014). The thiol-Michael addition click reaction: A powerful and widely used tool in materials chemistry. *Chem. Mat.* 26, 724–744

Neto, S. A., Forti, J. C., Zucolotto, V., Ciancaglini, P., and Andrade, A. R. D. (2011). The kinetic behavior of dehydrogenase enzymes in solution and immobilized onto nanostructured carbon platforms. *Process Biochem.* 46, 2347–2352

Noort, D. and Hulst, A. (2003). Modification of human serum albumin by acrylamide at cysteine-34: A basis for a rapid biomonitoring procedure. *Arch. Toxicol.* 77, 543–545

Osheroff, N., Shelton, E., and Brutlag, D. (1981). Properties of H^+-translocating adenosine triphosphatase in vacuolar membranes of *Saccharomyces cerevisiae*. *J. Biol. Chem.* 256, 10859–10863

Osheroff, N., Shelton, E., and Brutlag, D. (1983). DNA topoisomerase II from *Drosophila melanogaster*: Relaxation of supercoiled DNA. *J. Biol. Chem.* 258, 9536–9543

Park, H.-W., Ma, Z., Jiang, S., Robinson, R. C., and Endow, S. A. (2017). Structural basis of small molecule ATPase inhibition of a human mitotic kinesin motor protein. *Nature* 7, 1–12

Raj, H., Szymanski, W., Villiers, J. D., Rozeboom, H., Veetil, V., Reis, C., et al. (2012). Engineering methylaspartate ammonia lyase for the asymmetric synthesis of unnatural amino acids. *Nat. Chem.* 4, 478–484

RCSB PDB Ligand Summary ROP (2022). Propionamide https://www.rcsb.org/ligand/ROP [Accessed: January 5th, 2023]

Riera, F. and Álvarez, A. (2014). Influence of temperature and pH on the antigen-binding capacity of immunoglobulin G in cheese whey derived from hyper-immune milk. *Int. Dairy J.* 37, 111–116

Roos, G., Foloppe, N., and Messens, J. (2013). Understanding the pk_a of redox cysteines: The key role of hydrogen bonding. *Antioxid. Redox Signal.* 18, 94–127

Sciandrello, G., Mauro, M., Caradonna, F., Catazaro, I., Marghereth, S., and Giusi, B. (2010). Acrylamide catalytically inhibits topoisomerase II in V79 cells. *Toxicol. In Vitro* 24, 830–834

Selby, C. (1990). Sex hormone binding globulin: origin, function and clinical significance. *Ann. Clin. Biochem.* 27, 532–541

Sheng, Q., Zou, H.-C., Lü, Z.-R., Park, Y.-D., and Yao, S.-J. (2009). Effects of acrylamide on the activity and structure of human brain creatine kinase. *Int. J. Mol. Sci.* 10, 4210–4222

Shimizu, A., Suzuki, F., and Kato, K. (1983). Characterization of $\alpha\alpha$, $\beta\beta$, $\gamma\gamma$ and $\alpha\gamma$ human enolase isozymes, and preparation of hybrid enolases ($\alpha\gamma$, $\beta\gamma$ and $\alpha\beta$) from homodimeric forms. *Biochem. Biophys. Acta* 748, 278–284

Sickles, D., Brady, S., Testino, A., Friedman, M., and Wrenn, R. (1996). Direct effect of the neurotoxicant acrylamide on kinesin-based microtubule motility. *J. Neurosci. Res.* 46, 7–17

Sickles, D., Sperry, A., Testino, A., and Friedman, M. (2007). Acrylamide effects on kinesin-related proteins of the mitotic/meiotic spindle. *Toxicol. Appl. Pharmacol.* 222, 111–121

Studer, G., Biasini, M., and Schwede, T. (2014). Assessing the local structural quality of transmembrane protein models using statistical potentials (QMEANBrane). *Bioinformatics* 30, i505–i511

Studer, G., Rempfer, C., Waterhouse, A. M., Gumienny, R., Haas, J., and Schwede, T. (2020). GQMEANDisCo – distance constraints applied on model quality estimation. *Bioinformatics* 36, 1765–1771

Szasz, G., Gruber, W., and Bernt, E. (1976). Creatine Kinase in Serum: 1. Determination of Optimum Reaction Conditions. *Clin. Chem.* 22, 650–656

Talapatra, S., Harker, B., and Welburn, J. P. (2015). The C-terminal region of the motor protein MCAK controls its structure and activity through a conformational switch. *eLife* 4:e06421, 1–21

Tong, G. C., Cornwell, W. K., and Means, G. E. (2004). Reactions of acrylamide with glutathione and serum albumin. *Toxicol. Lett.* 147, 127–131

van Zundert, G., Rodrigues, J., Trellet, M., Schmitz, C., Kastritis, P., Karaca, E., et al. (2016). The HADDOCK2.2 web server: User-friendly integrative modeling of biomolecular complexes. *J. Mol. Biol.* 428, 720–725

Verhey, K. J., Lizotte, D. L., Abramson, T., Barenboim, L., Schnapp, B. J., and Rapoport, T. A. (1998). Light chain–dependent regulation of kinesin's interaction with microtubules. *J. Cell Biol.* 143, 1053–1066

Weber, B., SW, S. K., Varsani, A., Cowan, D., Hunter, R., Venter, G., et al. (2013). The mechanism of the amidases: mutating the glutamate adjacent to the catalytic triad inactivates the enzyme due to substrate mispositioning. *J. Biol. Chem.* 288, 28514–28523

Whiteheart, S., Rossnagel, K., Buhrow, S., Brunner, M., Jaenicke, R., and Rothman, J. (1994). N-ethylmaleimide-sensitive fusion protein: A trimeric ATPase whose hydrolysis of ATP is required for membrane fusion. *J. Cell Biol.* 126, 945–954

Wong, A. S., Lui, W.-Y., Hui, I. T., and Lee, W. M. (2001). Rabbit sex hormone-binding globulin: expression in the liver and testis during postnatal development and structural characterization by truncated proteins. *Int. J. Androl.* 24, 165–174
